# An Extensive Quality Control and Quality Assurance (QC/QA) Program Significantly Improves Inter-Laboratory Concordance Rates of Flow-Cytometric Minimal Residual Disease Assessment in Acute Lymphoblastic Leukemia: An I-BFM-FLOW-Network Report

**DOI:** 10.3390/cancers13236148

**Published:** 2021-12-06

**Authors:** Margarita Maurer-Granofszky, Angela Schumich, Barbara Buldini, Giuseppe Gaipa, Janos Kappelmayer, Ester Mejstrikova, Leonid Karawajew, Jorge Rossi, Adın Çınar Suzan, Evangelina Agriello, Theodora Anastasiou-Grenzelia, Virna Barcala, Gábor Barna, Drago Batinić, Jean-Pierre Bourquin, Monika Brüggemann, Karolina Bukowska-Strakova, Hasan Burnusuzov, Daniela Carelli, Günnur Deniz, Klara Dubravčić, Tamar Feuerstein, Marie Isabel Gaillard, Adriana Galeano, Hugo Giordano, Alejandro Gonzalez, Stefanie Groeneveld-Krentz, Zsuzsanna Hevessy, Ondrej Hrusak, Maria Belen Iarossi, Pál Jáksó, Veronika Kloboves Prevodnik, Saskia Kohlscheen, Elena Kreminska, Oscar Maglia, Cecilia Malusardi, Neda Marinov, Bibiana Maria Martin, Claudia Möller, Sergey Nikulshin, Jorge Palazzi, Georgios Paterakis, Alexander Popov, Richard Ratei, Cecilia Rodríguez, Elisa Olga Sajaroff, Simona Sala, Gordana Samardzija, Mary Sartor, Pamela Scarparo, Łukasz Sędek, Bojana Slavkovic, Liliana Solari, Peter Svec, Tomasz Szczepanski, Anna Taparkou, Montserrat Torrebadell, Marianna Tzanoudaki, Elena Varotto, Helly Vernitsky, Andishe Attarbaschi, Martin Schrappe, Valentino Conter, Andrea Biondi, Marisa Felice, Myriam Campbell, Csongor Kiss, Giuseppe Basso, Michael N. Dworzak, on behalf of I-BFM-FLOW-Network

**Affiliations:** 1 Children’s Cancer Research Institute, Medical University of Vienna, 1090 Vienna, Austria; margarita.maurer@ccri.at (M.M.-G.); angela.schumich@ccri.at (A.S.); 2 Pediatric Hematology, Oncology and Stem Cell Transplant Division, Maternal and Child Health Department, University of Padova, 35122 Padova, Italy; barbara.buldini@unipd.it (B.B.); pamela.scarparo@unipd.it (P.S.); elena.varottox@gmail.com (E.V.); giuseppe.basso@unipd.it (G.B.); 3 M. Tettamanti Foundation Research Center, Department of Pediatrics, University of Milano-Bicocca, 20900 Monza, Italy; g.gaipa@asst-monza.it (G.G.); o.maglia@asst-monza.it (O.M.); simona.sala@hsgerardo.org (S.S.); 4 Department of Laboratory Medicine, University of Debrecen, 4032 Debrecen, Hungary; kappelmayer@med.unideb.hu (J.K.); hevessy@med.unideb.hu (Z.H.); 5 Department of Paediatric Haematology and Oncology, University Hospital Motol, 150 06 Prague, Czech Republic; ester.mejstrikova@lfmotol.cuni.cz (E.M.); ondrej.hrusak@lfmotol.cuni.cz (O.H.); 6 Department of Pediatric Oncology and Hematology, Charité Berlin, 10117 Berlin, Germany; leonid.karawajew@charite.de (L.K.); stefanie.krentz@charite.de (S.G.-K.); 7 Cellular Immunology Laboratory, Hospital de Pediatria “Dr. Juan P. Garrahan”, Buenos Aires C1245, Argentina; jorro98@yahoo.com.ar (J.R.); sajaroffelisa@yahoo.com (E.O.S.); 8 Department of Immunology, Aziz Sancar Institute of Experimental Medicine, Istanbul University, 34452 Istanbul, Turkey; suzancinar@yahoo.com (A.Ç.S.); gdeniz@istanbul.edu.tr (G.D.); 9 LEB Laboratorio, Servicio de Hematologia Hospital Penna, Bahia Blanca B8000, Argentina; evangeagriello@hotmail.com; 10 P.&A. Kyriakou Children’s Hospital of Athens, 115 27 Athens, Greece; tanastasiou@yahoo.gr; 11 Laboratory—Flow Cytometry, Citomlab, Buenos Aires C1406AWK, Argentina; virnab1@gmail.com; 12 1st Department of Pathology and Experimental Cancer Research, Semmelweis University, 1085 Budapest, Hungary; barna.gabor@med.semmelweis-univ.hu; 13 Division of Laboratory Immunology, Department of Laboratory Diagnostics, University Hospital Centre Zagreb & School of Medicine, 10000 Zagreb, Croatia; drago.batinic@gmail.com (D.B.); klara.dubravcic@zg.t-com.hr (K.D.); 14 Department of Oncology and Children’s Cancer Research Center, University Children’s Hospital, 8032 Zurich, Switzerland; Jean-Pierre.Bourquin@kispi.uzh.ch (J.-P.B.); Claudia.Moeller@kispi.uzh.ch (C.M.); 15 Department of Hematology, University Hospital Schleswig-Holstein, 24105 Kiel, Germany; m.brueggemann@med2.uni-kiel.de (M.B.); Saskia.Kohlscheen@uksh.de (S.K.); 16 Department of Clinical Immunology, Institute of Pediatrics, Jagiellonian University Medical College, 31-008 Krakow, Poland; k.bukowska-strakova@uj.edu.pl; 17 Center of Competence “PERIMED”, Department of Pediatrics, Department of Microbiology and Clinical Immunology, Medical University Plovdiv, 4002 Plovdiv, Bulgaria; hassan_md@abv.bg; 18 Hospital Guillermo Rawson, San Juan J5400, Argentina; citometriahgr@gmail.com; 19 The Rina Zaizov Division of Pediatric Hematology-Oncology, Schneider’s Children’s Medical Center, Petah Tikva 4920235, Israel; TamarPo1@clalit.org.il; 20 Bioquimica, Inmunologia, Hospital de Ninos Rocardo Gutierrez, Buenos Aires C1425EFD, Argentina; migaillard@yahoo.com; 21 Flow Cytometry Laboratory, FUNDALEU, Buenos Aires C1114, Argentina; galeanoadriana@hotmail.com; 22 Fundación Pérez Scremini, Pediatric Hematology-Oncology Service, Pereira Rossell Hospital, Montevideo 11600, Uruguay; giordanohugo8@gmail.com; 23 PAPSI Laboratorio Mendoza, Rosario S2000, Argentina; papsi.labo.mendoza@gmail.com; 24 Flow Cytometry Laboratory, Provincial Histocompatibility Reference Centre, CUCAIBA, Buenos Aires C1114, Argentina; citometriacucaiba@gmail.com; 25 Flow Cytometry Laboratory, Department of Pathology, Clinical Centre, University of Pécs, 7622 Pécs, Hungary; jakso.pal@pte.hu; 26 Department of Cytopathology, Institute of Oncology, 1000 Ljubljana, Slovenia; vkloboves@onko-i.si; 27 Faculty of Medicine, University of Ljubljana, 1000 Ljubljana, Slovenia; 28 Clinical Laboratory Diagnostics and Metrology of NCSH “OHMATDYT”, Ministry of Heath of Ukraine, 01601 Kiev, Ukraine; elena_kreminska@ukr.net; 29 Hospital de Clinica Jose de San Martin, Buenos Aires C1120, Argentina; chechimalu@gmail.com; 30 PINDA, Chilean National Pediatric Oncology Group, Hospital Roberto del Rio, Universidad de Chile, Santiago 8380418, Chile; nedamarinov@yahoo.com (N.M.); myriam.campbell@redsalud.gov.cl (M.C.); 31 Laboratorio CEBAC SRL, Posadas N3300, Argentina; bibiana_cebac@hotmail.com; 32 Hematopathology and Flow Cytometry Division, Children’s Clinical University Hospital, LV-1004 Riga, Latvia; nikulshin@gmail.com; 33 IICT Labs, Rosario S2000, Argentina; jpalazzi@iictlabs.com; 34 Georgios Gennimatas General Hospital of Athens, 115 27 Athens, Greece; genpater@gmail.com; 35 Laboratory of Leukemia Immunophenotyping, Dmitry Rogachev National Medical Research Center of Pediatric Hematology, Oncology and Immunology, 117997 Moscow, Russia; uralcytometry@gmail.com; 36 Clinic for Hematology and Tumor Immunology, HELIOS Klinikum Berlin-Buch, 13125 Berlin, Germany; Richard.Ratei@helios-gesundheit.de; 37 Hospital Nacional de Clínicas, Universidad Nacional de Córdoba, Cordoba X5000HUA, Argentina; cecirodr@hotmail.com; 38 Laboratory for Flow Cytometry and Immunology, Institute for Health and Protection of Mother and Child of Serbia, 11070 Belgrade, Serbia; goca.samardzija@gmail.com (G.S.); bojana.slavkovic@gmail.com (B.S.); 39 The Children’s Hospital at Westmead, Sydney, NSW 2145, Australia; mary.sartor@health.nsw.gov.au; 40 Department of Microbiology and Immunology, Medical University of Silesia, 40-055 Katowice, Poland; lsedek@sum.edu.pl; 41 Servicio de Bioquimica, Hospital Posadas, Buenos Aires B1684, Argentina; lilianasolari@intramed.net; 42 National Institute of Children’s Diseases, 831 01 Bratislava, Slovakia; peter.svec@gmail.com; 43 Department of Pediatric Hematology and Oncology, Zabrze, Medical University of Silesia, 40-055 Katowice, Poland; szczep57@poczta.onet.pl; 44 Department of Pediatric Oncology Hippokration General Hospital, 546 42 Thessaloniki, Greece; nprintza@in.gr; 45 Pediatric Hematology and Oncology, Hospital Sant Joan de Deu, 08950 Barcelona, Spain; montserrat.torrebadell@sjd.es; 46 Department of Immunology & Histocompatibility, “Agia Sophia” Children’s Hospital, 115 27 Athens, Greece; emainasgr@yahoo.gr; 47 Hematology Lab, Sheba Medical Center, Ramat Gan 52621, Israel; Helly.Vernitsky@sheba.health.gov.il; 48 St. Anna Children’s Hospital, Department of Pediatrics, Medical University of Vienna, 1090 Vienna, Austria; andishe.attarbaschi@stanna.at; 49 Department of Pediatrics, University Medical Center SchleswigHolstein, Christian-Albrechts-University of Kiel, 24118 Kiel, Germany; m.schrappe@pediatrics.uni-kiel.de; 50 Clinica Pediatrica University degli Studi di Milano Biococca, Fondazione MBBM, 20900 Monza, Italy; v.conter@asst-monza.it (V.C.); a.biondi@asst-monza.it (A.B.); 51 Department of Hematology and Oncology, Hospital de Pediatria “Dr. Juan P. Garrahan”, Buenos Aires C1245, Argentina; marisa.felice2013@gmail.com; 52 Division of Pediatric Hematology-Oncology, Department of Pediatrics, Faculty of Medicine, University of Debrecen, 4032 Debrecen, Hungary; kisscs@med.unideb.hu

**Keywords:** acute lymphoblastic leukemia, minimal residual disease, flow cytometry, quality control program

## Abstract

**Simple Summary:**

Standardization of flow-cytometric assessment of minimal residual disease in acute lymphoid leukemia (ALL) is necessary to allow concordant multicentric application of the methodology. This is a prerequisite for internationally collaborative trials, such as the AIEOP-BFM-ALL and the ALL IC-BFM trial. We developed and applied a comprehensive training and quality control program involving a large number of international laboratories within the I-BFM consortium to complement standardization of the methodology with an educational component as well as with persistent quality control measures to allow large ALL treatment trials which use multi-laboratory FCM-MRD assessments for risk stratification of pediatric patients with ALL.

**Abstract:**

Monitoring of minimal residual disease (MRD) by flow cytometry (FCM) is a powerful prognostic tool for predicting outcomes in acute lymphoblastic leukemia (ALL). To apply FCM-MRD in large, collaborative trials, dedicated laboratory staff must be educated to concordantly high levels of expertise and their performance quality should be continuously monitored. We sought to install a unique and comprehensive training and quality control (QC) program involving a large number of reference laboratories within the international Berlin-Frankfurt-Münster (I-BFM) consortium, in order to complement the standardization of the methodology with an educational component and persistent quality control measures. Our QC and quality assurance (QA) program is based on four major cornerstones: (i) a twinning maturation program, (ii) obligatory participation in external QA programs (spiked sample send around, United Kingdom National External Quality Assessment Service (UK NEQAS)), (iii) regular participation in list-mode-data (LMD) file ring trials (FCM data file send arounds), and (iv) surveys of independent data derived from trial results. We demonstrate that the training of laboratories using experienced twinning partners, along with continuous educational feedback significantly improves the performance of laboratories in detecting and quantifying MRD in pediatric ALL patients. Overall, our extensive education and quality control program improved inter-laboratory concordance rates of FCM-MRD assessments and ultimately led to a very high conformity of risk estimates in independent patient cohorts.

## 1. Introduction

The application of flow cytometric minimal residual disease measurement (FCM-MRD) has become a routine clinical practice in most current study protocols worldwide, in order to assign patients to different risk groups and to tailor treatment intensity [1,2,3,4,5,6]. FCM-MRD is applicable in almost all (>98%) patients with lymphoblastic leukemia and can reach sensitivities similar to a quantitative real-time polymerase chain reaction (RQ-PCR) (≥10^−5^) if it is ensured that the laboratories involved in MRD testing are able to reliably detect and quantify leukemic cells, even in the low MRD range [1]. Several national centers and international consortia previously worked on standardizing the FCM-MRD methodology in multi-centric settings to improve inter-laboratory concordance rates, and to assure sensitive and accurate MRD assessments [5,7,8,9,10,11]. The allied Associazione Italiana di Ematologia Oncologia Pediatrica (AIEOP) and the Berlin-Frankfurt-Münster (BFM) FCM-MRD study groups, which have collaborated since May 2000, initiated a standardization program based on education of personnel, technical alignment, and on continued quality control (QC) [9]. This study, involving four experienced national reference laboratories, proved that FCM-MRD can be standardized for multi-centric application in large trials. Such a multi-facetted QC/quality assurance (QA) program included the standardization of the methodology, continuous staff training, performance reviews through sample and list-mode data (LMD) exchange, as well as the assessment of concordance of “on trial” MRD results from independent patient cohorts [9]. Similarly, an educational component complementing the standardization of the FCM-MRD methodology was necessary to warrant concordant quality of MRD assessment in a study involving eight experienced centers in North America [12].

In the current study, we performed a unique and comprehensive training and QC/QA program involving a total of 41 laboratories collaborating in the international (I)-BFM consortium. Of note, many associated laboratories of the network were inexperienced when entering the program. Our approach consists of four major building blocks: (i) a twinning maturation program, where well-trained expert laboratories work closely with more inexperienced laboratories to improve their performance in individual feedback sessions; (ii) the obligatory participation in external QA programs (spiked sample send around), e.g., United Kingdom National External Quality Assessment Service (UK NEQAS); (iii) regular participation in the analysis of LMD file ring trials (data file send arounds) with an educational component, where difficult and discordant cases are jointly discussed; and (iv) surveys of independent data derived from trial results, where the agreement of different centers, with respect to MRD results from their locally recruited patient cohorts, were tested.

We demonstrate that the training of laboratories using experienced twinning partners along with continuous educational feedback, significantly improves the performance of laboratories to detect and quantify MRD in pediatric ALL patients. In addition to a standardized methodology, educational feedback and quality oversight by a coordinating structure is required to ensure the sufficient quality of MRD assessment for clinical routine.

## 2. Materials and Methods

### 2.1. Samples

MRD investigations were approved as part of the international trial by the institutional ethical committees and were conducted according to informed consent guidelines. For list-mode file ring trials, we used bone marrow (BM) samples from pediatric patients (0–≤18 years of age) recruited in the AIEOP-BFM-ALL clinical trial in Vienna (AUT).

In total, data from 101 BM samples from regenerative post-induction time-points of pediatric patients with B-ALL were used for the LMD file ring trials (“B-cell precursor (BCP) Regeneration”) during 2016–2020. These regenerative bone marrows are usually characterized by the appearance of BCP subsets at different stages of (BCP 1–3) [13].

Of the 101 BM samples, 53 were FCM-MRD negative, and 48 samples were FCM-MRD positive (range 0.004–18.16%). If available, RT-PCR data served as an independent confirmation of the FCM-MRD target votes. The target MRD values were calculated as a median from six experienced and matured AIEOP-BFM reference laboratories (Vienna, Monza, Padova, Prague, Kiel and Berlin).

#### Sample Preparation

Detailed standard operating procedures (SOP) from the AIEOP-BFM (Supplementary Materials File S1 “SOP FLOW-MRD 1.5 AEIOP-BFM”) as well as from the intercontinental ALL- IC reference centers can be found in the Supplementary Materials (Supplementary Materials File S2 and S3 “SOP ALL-IC 2009” and “Amendment to SOP ALL-IC-BFM_2014”). Samples for the generation of LMD files were prepared at the coordinating center in Vienna briefly as follows: whole BM samples were stained either with a two-tube approach (7- and 8-color panel, 2016–2017, Table S1) or with a single, dried, 10-color format tube (ReALB, DuraClone^TM^, Beckman Coulter, Brea, CA, USA), Table S1) (2018–2020) for 15′ and lysed with a fixing lysis reagent (BD Lyse, Becton Dickinson, Franklin Lanes, Evansville, EL, USA) for another 15′. Samples were washed once with 1× phosphate-buffered saline (PBS, Gibco, Thermo Fisher Scientific Inc., Waltham, MA, USA), resuspended in 200 µL 1×PBS and measured immediately. Samples were acquired using an LSRII flow cytometer (Becton Dickinson) and analyzed at the reference center using DIVA software (Becton Dickinson). Syto41 staining was used to exclude non-nucleated events such as erythrocytes, platelets, and debris. For detailed information on the gating strategy, see Supplementary Materials S1–S3. MRD was reported as a percentage of Syto41-positive cells with exclusion of doublets using FSC-H/FSC-A gating. Minimal residual disease positivity was defined as an accumulation of ≥10 cells with a leukemic phenotype [9].

## 2.2. I-BFM-FLOW Twinning and Maturation Program

A total number of 29 laboratories participated in the twinning and maturation program of which 22 were ALL IC-BFM (Acute Lymphoblastic Leukemia Intercontinental Berlin-Frankfurt-Münster) trainee laboratories. Seven experienced and already matured AIEOP-BFM laboratories served as twinning and training partners. “Maturation” is granted to a center according to specific criteria and is defined as the permission to release MRD results for clinical use without further control by the partner laboratory (“twinning partner”). “Maturation” can be granted for either B-lineage ALL-MRD at day 15/d33 or at regenerative time-points, and for T-ALL-MRD (independent of time-point). “Maturation” is granted after completion of a series of at least 25 different locally and consecutively recruited patient sample pairs (diagnosis and MRD time-points) per lineage, the analysis of which, along with the results, was promptly submitted to the twinning partner for review. Harmonization and technical alignment of the FCM-MRD technology including machine settings, panel composition, sample preparation as well as staining was an essential part of the twinning maturation program and took place before the test series was started. During the initial phase of the program, an intensive interaction and discussion between the trainee laboratory and its twinning partner following each case ensured a rapid and effective training progress. Therefore, the first half of the samples serves, for the most part, an intensive training, the immediate success of which should and must be proven with the second half of the samples.

In the second half of the sample series, each gross error then requires a further three MRD assessments of consecutive cases without errors to achieve maturation. Gross errors are defined as inadequacy of staining and analysis, errors in blast population, or gross misinterpretation or misdocumentation on a predefined report sheet. Provided that no more gross errors in identifying and/or quantifying MRD are detected in the most recent half (i.e., 12 patients) of the sample series, maturation is granted. For a detailed description of the guidelines, see Supplementary Materials Files S4 and S5 “I-BFM FLOW Twinning Maturation” and “Maturation Certificate”. Feedback by training laboratories was mainly provided via email and/or phone or during training visits and included recommendations regarding further optimization of technical issues (e.g., compensation, etc.) as well as the gating strategy or explanations of phenotypic properties of normal and aberrant cells, respectively.

## 2.3. External Quality Assessment (EQA)

UK NEQAS (https://ukneqas.org.uk/ (accessed on 30 November 2021) and [14]) provides external quality assessment/proficiency testing for all major aspects of clinical laboratory testing including “Minimal Residual Disease for ALL by flow cytometry”. UK NEQAS ships stabilized blood samples spiked with B-ALL blasts to participating centers. Individual results are compared to the robust mean of all the submitted values. UK NEQAS sends out two samples with different MRD values per trial and usually issues four wet trials per year. This program was used by the I-BFM network for EQA. The local results of all I-BFM centers submitting results were evaluated in a centralized manner by the I-BFM coordinating center in Vienna.

## 2.4. List-Mode-Data File Ring Trials (LMD Ring Trial)

Anonymized list-mode files were prepared and sent out by the I-BFM FLOW coordinating center in Vienna. A set of at least 20 files containing data of regenerating bone marrow samples with either negative MRD or different positive levels of MRD was sent to the participating laboratories once a year for blinded LMD file interpretation. The participating laboratories were members of the AIEOP-BFM or ALL-IC BFM consortia or cooperating laboratories. Reported MRD values that were either more than three times or less than one-third of the target MRD value (concordance margin, see [9]) were considered as false assessments. The target MRD value was calculated as a median from six experienced and matured AIEOP-BFM reference laboratories (Vienna, Monza (ITA), Padova (ITA), Prague (CZE), Kiel (GER) and Berlin (GER)). Laboratories, which reported false MRD values in >10%–≤25% of cases were flagged with a warning and reports with >25% false MRD values were considered to be critical.

## 2.5. Independent Data Survey

We conducted a survey among eight AIEOP-BFM reference laboratories to collect FCM-MRD-based risk classification data within the AIEOP-BFM-ALL 2009 trial. The following reference laboratories participated in this survey: Berlin, Monza, Padova, Prague, Vienna, Zurich (CH), Sydney (AUS) and the two reference laboratories of Israel (ISR). Details of all requested data can be found in Supplementary Materials File S6, “Data query”. According to the AIEOP-BFM ALL 2009 study protocol, the criteria for assignment of patients to either flow low risk (FLR), flow medium risk (FMR) or flow high risk (FHR) were defined as follows: percentage of blasts in the BM at day 15 of induction therapy ≤0.1% FLR, >0.1% <10% FMR, ≥10% FHR.

## 2.6. Statistical Analysis

The concordance of independent flow-risk estimate data was assessed using GraphPad Prism ver. 8.3.0 (GraphPad Software, San Diego, CA, USA) and Two-Way ANOVA (Tukey test for multiple comparison correction). We used http://www.analyticscalculators.com (accessed on 30 November 2021) for computation of exact test probabilities for 2 × 2 and 2 × 3 Contingency Tables [15,16].

## 3. Results

### 3.1. Twinning Maturation Program

In total, 682 samples were processed and analyzed by the 22 trainee laboratories using diagnostic methodology based on central standard operating procedures (SOPs) (see Supplementary Materials File S1–S3), and the data were sent to the training laboratories for review (Table 1).

**Figure 1:**
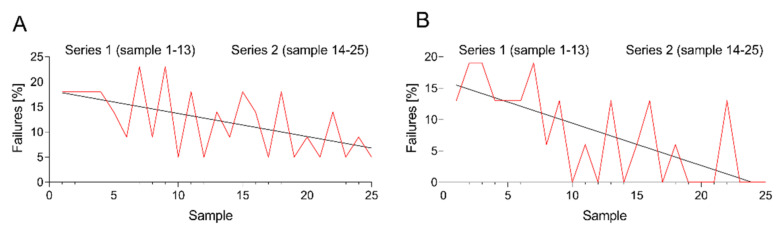
Twinning maturation program. (**A**) Number of failures by all 22 laboratories in the main sample set (samples 1–25) (**B**) Number of failures of the 16 laboratories that finished within a range of a maximum of 31 samples (31 samples = mean number of samples needed for maturation).

According to the guidelines, a minimum of 25 locally and consecutively recruited samples per lineage (B- or T-ALL, Table S1) had to be assessed by the trainees without gross errors in the second half of the samples (i.e., in the last 12 samples). For each gross error, three additional samples had to be processed, analyzed and sent again for review. Overall, out of the 682 analyzed B-ALL samples, MRD assessment was erroneous in 11.6% of samples (Table 2). In total, laboratories required a median number of 28 (range 25–57) and a mean number of 31 ± 8.8 samples to reach maturation status (Table 1). The difference in the number or errors in the first (Series 1: sample 1–13) and second half (Series 2: sample 14–25) of the sample set was marginally not statistically significant (14.7% vs. 9.5%), but reached significance when considering the entire sample set (Series 1: sample 1–13 vs. Series 3: sample 14-pX) (14.7% vs. 9.3%; *p* < 0.05) (Table 2 and Figure 1). Sixteen laboratories achieved maturation status within thirty-one samples (which is the mean number of samples needed for maturation). In those 16 laboratories, there was a significant performance improvement between sample series 1 and 2 (11.1% vs. 3.1%, *p* < 0.01; Table S2 and Figure 1). Overall, the initial performance of the training laboratories was variable, and six laboratories required more than 31 samples to achieve maturation status. The continuous educational feedback during the twinning maturation process significantly improved the ability of the trainee laboratories to correctly detect and quantify MRD in B- (Table 2) and T-ALL (data not shown) samples.

### 3.2. External Quality Assessment

The United Kingdom National External Quality Assessment Scheme (UK NEQAS) for leukocyte immunophenotyping provides external quality assessment (EQA)/proficiency testing (PT) programs for minimal residual disease for ALL by FCM.

**Figure 2:**
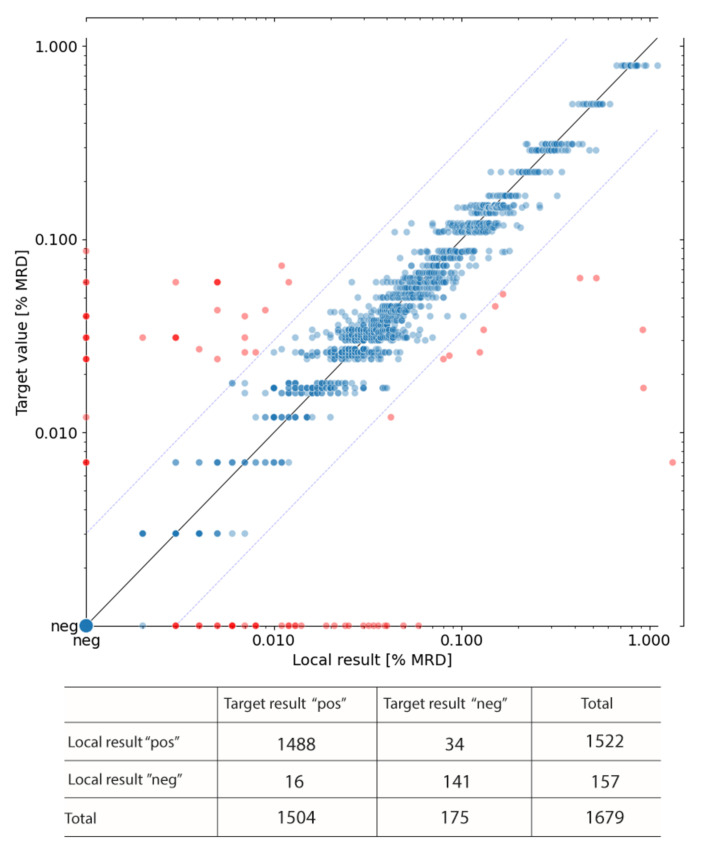
Correlation of the local MRD estimates of UK NEQAS samples analyzed by I-BFM-Flow laboratories. The target value represents the median of all submitted results. Red dots indicate outliers (i.e., MRD results outside the concordance margin, *n* = 78). Blue dots indicate concordant results (*n* = 1601). Positivity was defined as ≥10 MRD events among 3 × 10^5^ nucleated events, i.e., >0.003% (pos, positive; neg, negative).

We collected performance data from 29 issued UK NEQAS ring trials (containing 2 samples/ring trial) from up to 38 participating I-BFM-FLOW laboratories, and monitored their performance over time by correlating local results of I-BFM centers with target votes (Figure 2). Target votes were defined as the median of all submitted I-BFM center results. Reported MRD values within the concordance margin were considered as concordant (i.e., either more than three times or less than 1/3 of the target MRD value [9]), whereas MRD values outside the concordance margin were defined as outliers. In total, results of 1679 samples have been submitted and compared, of which 487 samples were submitted by AIEOP-BFM laboratories (*n* = 9) and 1192 by ALL IC-BFM and other associated laboratories (*n* = 29). Generally, AIEOP-BFM laboratories had a significantly lower proportion of outliers (2.3%) than ALL IC-BFM/other associated laboratories (5.6%) (*p* = 0.0043), which is in concordance with the higher number of long-term experienced centers within the AIEOP-BFM group. The overall discordance rate was 4.6% (Table 3 and Figure 2). We also assessed the concordance of local versus target votes regarding MRD positivity or negativity, where positivity was defined as ≥10 MRD events among 3 × 10^5^ nucleated events. Here, the concordance rate was 97%, specificity was 81% (141/175) and sensitivity was 99% (1488/1504) (Figure 2).

### 3.3. List Mode Data File Ring Trials (LMD Ring Trial)

For further continuous quality assessment and improvement, the performance of the I-BFM-FLOW laboratories was monitored regularly via LMD ring trials consisting of data files with either negative or varying levels of MRD from B- or T-ALL cases, or of data files from diagnosis for testing the ability of the reference laboratories for the correct lineage assessment of AL by immunophenotyping (see Table S3). The staining panel setup is shown in Table S1 (B- and T-ALL).

Since the detection and quantification of MRD by FCM is particularly challenging in B-ALL in periods of heavy hematopoietic regeneration, while it is relatively straight forward at early timepoints of therapy (i.e., d15), a special focus was on MRD training at regenerative timepoints.

**Figure 3:**
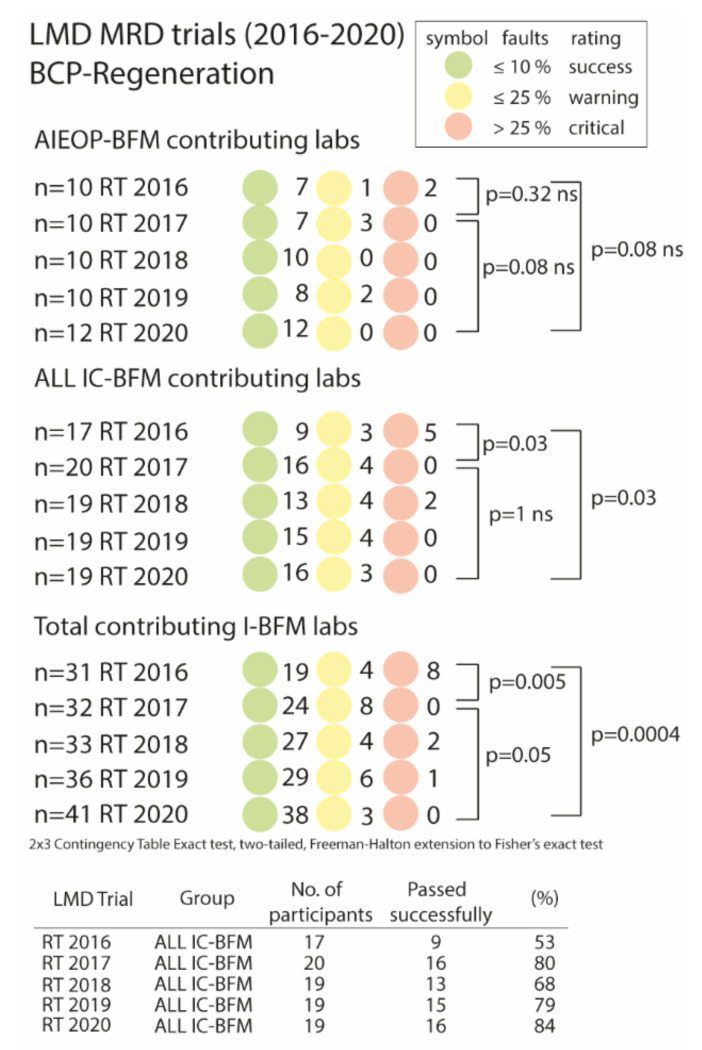
Regular ring trial tests using the list-mode file sent around reveal a significant performance improvement. LMD Files from 20 B-ALL cases at day 78 or after relapse therapies were sent out to all participating laboratories once a year. Laboratories, which reported false MRD values in >10%–≤25% of cases were flagged with a warning (yellow circle) and reports with >25% false MRD values were considered to be critical (red flag). Total contributing I-BFM labs contained AIEOP-BFM, ALL IC-BFM, and other associated laboratories. Statistical analysis was performed using a 3 × 2 contingency table and two-sided Fisher’s test.

A series of list mode data ring trials of B-ALL at regenerative time points (e.g., day 78 or after relapse therapies) is shown in Figure 3. LMD ring trials (RT) were organized once a year by the I-BFM-FLOW coordinating center in Vienna. The total number of participating laboratories (AIEOP-BFM, ALL IC-BFM as well as other associated laboratories) varied from 31 in the first RT 2016 up to 41 laboratories in RT 2020. In the first LMD ring trial, 19 out of 31 laboratories (61%) reported MRD values consistent with the target value, while four laboratories (13%) submitted >10% discordant values and eight laboratories (26%) were even considered critical with >25% discordant values (Figure 3, lower panel).

Samples that were incorrectly assessed by several centers were subjected to error analysis (*n* = 24) (Table S4). Most of the repeatedly misinterpreted samples were regenerative BM with a particularly high number of early B cell progenitors (hematogones) (18 out of 24 discordant samples; 75%) resulting mostly in false positive assessments in 13 out of these 18 samples (72%). Additionally, false negative assessments (5/18) as well as inaccurate gating leading to lower or higher MRD levels (5/18) occurred in this group of samples. Along with these considerations, common reasons for failure were an insufficient theoretical separation of blasts from hematogones by classical leukemia-associated immunophenotype (LAIP) markers or maker combinations (7/24), the absence of mature B-cells that could serve as a normal reference population (6/24), negative or only dim expression of CD34 on early BCP1 in burst regeneration (4/24), the co-existence of BCP1 and blasts with only a few aberrations of the latter (4/24), a rare and/or heterogeneous phenotype of the blasts (2/24), or the presence of only very few MRD cells (2/24) (Table S4). The most common errors, together with solutions and suggestions for improvements, were presented and discussed at regular joint meetings and educational material was disseminated to all participants (Supplementary Materials File S7 “Educational Slides”). During these educational feedback meetings, guidelines, and suggestions for better distinguishing hematogones from aberrant blasts were provided.

This educational feedback proved to be efficient and sustainable. In the second LMD ring trial (RT 2017), the overall performance of the laboratories significantly improved (*p* = 0.005). No laboratory submitted >25% discordant results while 8/32 laboratories (25%) were still assigned a warning flag (Figure 3, lower panel). In the last LMD ring trial (RT 2020), 93% of all laboratories succeeded, while no laboratory was assigned a critical status, and only three laboratories (7%) were assigned a warning flag.

Of the ALL IC-BFM laboratories, only 54% passed successfully in RT 2016, whereas this number increased to 80% in RT 2017 and remained at high levels (range 68–84%) in RT 2018–2020 (Figure 3, middle panel and table). There was no significant improvement when considering only the AIEOP-BFM laboratories, although the percentage of laboratories successfully passing the LMD ring trial improved from 70% in 2016 (7 out of 10) to 100% in 2020 (Figure 3, upper panel).

When assessing only those laboratories which were active from the beginning, results of the improvement status over time did not differ from the assessment of all participating laboratories (Figure S1).

**Figure 4:**
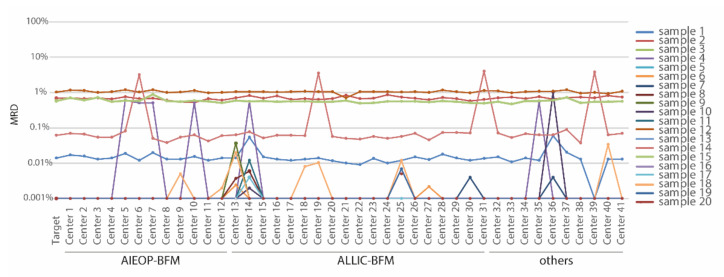
Plot from a representative list-mode file ring trial. RT 2020 comprised 20 regenerative bone marrow samples with either negative or varying levels of MRD. LMD files were sent out to 41 centers, all of which reported results. AIEOP-BFM n = 12, ALL IC-BFM = 19, and other I-BFM cooperating centers = 10. The target MRD value was calculated as a median from six experienced and matured AIEOP-BFM reference laboratories (Vienna, Monza, Padova, Prague, Kiel and Berlin). Two opportunities for educational intervention can be seen in this figure: (i) similar errors by four and six centers, respectively, in samples 14 and sample 4 representing a systematic error; and (ii) an accumulation of errors in single centers (see center 13 and 14) representing lack of experience.

A representative example of an LMD ring trial summary with data files from B-ALL samples of regenerative timepoints is illustrated in Figure 4.

### 3.4. Independent Data Concordance: A Retrospective Quality Control Measure

**Figure 5:**
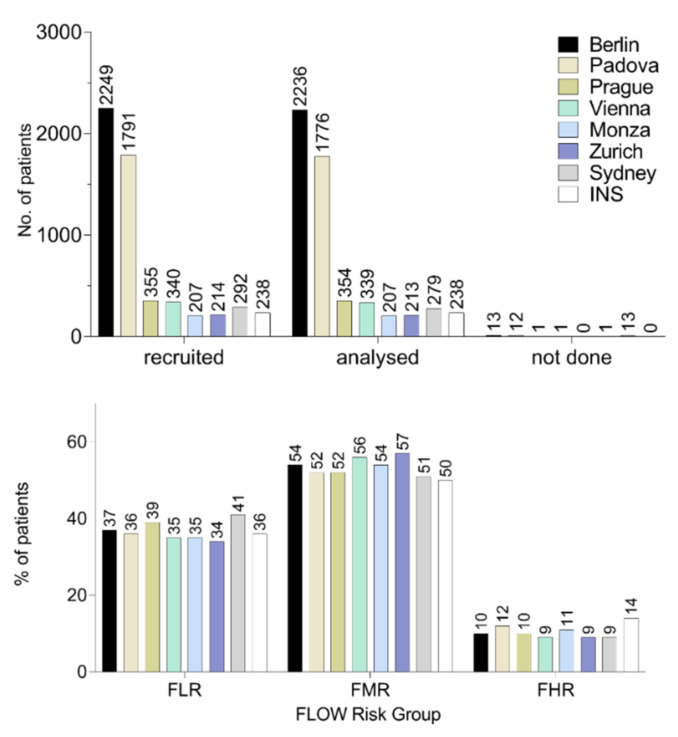
Homogeneity of B-ALL risk classification data of well-trained and matured centers within the AIEOP-BFM ALL 2009 trial. In a survey including nine AIEOP-BFM FLOW reference laboratories from eight countries, independent data of FCM-MRD-based risk classification of B-ALL on day 15 were collected and compared. Statistical analysis was performed using GraphPad Prism 8.3.0. FHR, Flow high risk; FLR, Flow low risk; FMR, Flow medium risk; INS, contains combined data from the two reference laboratories in Israel.

We tested the agreement of eight matured and highly experienced I-BFM centers regarding the distribution of patients in different FCM-based risk groups within the AIEOP-BFM-ALL 2009 trial (details on all requested data of the survey can be found in Supplementary Materials File S6 “Data query”). The percentage of ALL patients assigned to either the low-risk (FLR), medium-risk (FMR) or high-risk (FHR) group by FCM were highly concordant and there were no significant differences between the independent patient cohorts from the eight centers (Figure 5 (B-ALL) and Figure S2 (T-ALL)).

## 4. Discussion

This I-BFM-FLOW network report summarizes the comprehensive QC/QA program on FCM-MRD involving an unprecedented, intercontinental effort of two of the world’s largest pediatric ALL trial groups. As of November 2020, twenty-two trainee and seven expert trainer laboratories participated in a twinning maturation program and all trainee laboratories achieved maturation status, enabling them to report FCM-MRD results for clinical decision making without further control by partner laboratories according to the study group’s guidelines. Forty-one I-BFM-FLOW laboratories submitted results in the most recent LMD file send around (RT2020) and data on 1679 results of 29 issued external QA ring trials (UK NEQAS) of up to 38 laboratories participating per ring trial were collected. As shown in this report, these exercises were successfully developed, and maintained and controlled the proficiency of the many FCM laboratories involved in AIEOP-BFM and ALL IC-BFM trial conduct.

The harmonization and alignment of the technical aspects of the FCM-MRD methodology, including machine settings, panel composition, sample preparation, as well as sample staining between different centers, are important prerequisites for concordant multicenter applications [9]. Several previous studies showed that the harmonization of technical and methodological aspects is feasible, even in an international context [5,9,10,12]. This is particularly important for large, internationally collaborative trials, such as the AIEOP-BFM-ALL and the ALL IC-BFM trial, where the results of the different laboratories must be concordant for the correct stratification and risk-adapted treatment of patients. While the technical aspects of the FCM-MRD method can be well harmonized, the human factor, i.e., the interpretation of the electronic files of the acquired sample, is the actual Achilles heel of the methodology and strongly relies on operator skills and expert knowledge [9,12,17]. Hence, discrepancies in MRD values between laboratories can be due to wrong interpretations of the data [9,12]. This becomes especially apparent in the case of discrepancies in the results of FCM data file exchanges, where preanalytical and acquisition issues do not play a role.

Hence, we based the continuous quality control on two major cornerstones: firstly, the LMD file ring trials, where we focused on the assessment of the analysis performance of each laboratory; and, secondly, on the external UK NEQAS quality control program that allows the performance of the whole methodology to be assessed. Our LMD file ring trials also contain an educational component, where problematic cases are discussed in joint meetings. As part of such meetings, suggestions and solutions for the proper identification of the aberrant cells are provided. In the LMD ring trials, strong BCP regeneration in the bone marrow was found to be the main cause of incorrect MRD assessment. In most cases, these were false positive assessments because normal B cell progenitors (hematogones) were wrongly mistaken for leukemic cells. Therefore, we specifically selected cases with different stages of regeneration and different MRD levels for the LMD ring trials to train the laboratories, especially on such challenging samples. Although BCP regeneration does normally not occur at day 15 or at day 33, it becomes relevant at day 78 of BFM-type induction or during later follow-up time points [4]. While most previous studies were conducted using four-color techniques, panels with ≥6 colors significantly improved the specificity and sensitivity of MRD analysis [18,19,20] and the use of a single-tube with the addition of markers such as CD38, frequently allowed for a reliable differentiation between hematogones and leukemic cells [19,20].

Our approach has some limitations. There are situations where standard panels do not allow for a correct identification of MRD events, even by the experts themselves, unless additional markers, which are useful for such rare cases, are deployed for a better distinction of aberrant cells [21]. In addition, time and staff constraints form a bottleneck for the continued QC and training necessities in a large consortium such as the I-BFM FLOW network. Therefore, approaches with the aim of supporting the operator by using an objective, automated, FCM-analysis tool based on machine learning techniques are necessary and are already being developed [17,22,23,24]. Such a tool may not only relevantly decrease technical variability inherent in manual gating strategies but may also serve as a virtual training partner in twinning programs, such as those presented here to reduce the need for the laborious and costly face-to-face trainings or remote educational feedback loops of trainees with experts. With respect to the survey of independent on-trial data, it does not allow for short-term quality control and immediate intervention and improvements, however, can be used as retrospective evidence of the reliability of the data.

## 5. Conclusions

Our extensive training and quality assurance program contribute significantly to improving and continuously ensuring the performance of individual laboratories. Of note, the structure based on a coordinating center responsible for quality oversight and feedback proved particularly effective and relevant. Overall, this led to a very high inter-laboratory concordance rate of FCM-MRD assessments and to a high conformity of risk estimates in independent patient cohorts. This is essential for conducting large ALL treatment trials which use multi-laboratory FCM-MRD assessments for stratification.

“The I-BFM FLOW-network is open to further national or trial-based reference laboratories to join the quality program for providing excellent, quality-assured diagnostics to young patients with acute leukemia and for promoting collaborative research.”

## Figures and Tables

**Table 1:** Overview of the I-BFM FLOW twinning program.

Overview	
**Nº of trainee labs ^§^**	22
Nº of expert trainer labs ^*^	7
Nº of samples reviewed	682
Nº of samples required for maturation/lab	25
Median needed [range]	28 (25–57)
Mean needed [SD]	31 (8.8)

^§^ Laboratories of the ALL IC-BFM FLOW network; * Laboratories of the i-BFM FLOW network.

**Table 2:** Twinning maturation program. The twinning maturation program significantly reduces the number of wrong MRD assessments after a first series of training samples.

All 22 Trainee Laboratories				
Main Sample Set (Sample 1–25)		Series 1 (Sample 1–13)	Series 2 (Sample 14–25)	Series 3 (Sample 14–pX) ^§^
Total Nº of reported results	550	286	264	396
Nº of failures	67	42	25	37
Failures [%]	12.2	14.7	9.5	9.3
**All samples (sample 1–57)**				
Total Nº of reported results	682			
Nº of failures	79			
Failures [%]	11.6			

^§^ pX = last sample (sample57 latest).

**Table 3:** Table summarizing participation and performance of AIEOP-BFM and ALL IC-BFM laboratories in UK NEQAS “Minimal residual disease for ALL by Flow cytometry” ring trials. Data evaluation was completed with each single ring trial by the I-BFM FLOW coordinating center in Vienna. In case of outliers or missing results, the local/individual center was contacted, and corrective measures were discussed. Note that the № of possible results is higher than the number of submitted results, because not all laboratories submitted all results.

**Participation**			
Nº of issued trials ^a^	29		
Nº of participating laboratories	29–38		
Nº of possible results	1925		
Nº of submitted results	1679		
**Performance**			
	AIEOP-BFM	ALL IC-BFM/others	Total
Nº of submitted results	487	1192	1679
Nº of Outliers ^b^	11	67	78
Outliers ^b^ [%]	2.3	5.6	4.6
	*p* = 0.0043 ^c^		

^a^ Evaluation period: starting with trial number 131403 until 202103; ^b^ Outliers are definded as reported MRD levels ≥ 3× larger or smaller than the target value; i.e., MRD-level > half a log up or down in a log10-correlation (concordance margin); Target MRD levels: median of all reported values of the sample derived from all I-BFM laboratories submitting a result. ^c^ Statistics was performed using a 2 × 2 contingency table and Two sided Fisher’s test.

## Data Availability

For original data, please contact dworzak@stanna.at.

## Supplementary Materials

The following data are available online at https://www.mdpi.com/article/10.3390/cancers13236148/s1, Figure S1: Results of regular ring trial tests of core laboratories that were involved from the beginning of the program. Figure S2: Homogeneity of T-ALL risk classification data of well trained and matured centers within the AIEOP-BFM 2009 trial, Table S1: Antibodies used for MRD assessment in samples from pediatric patients with B-ALL, Table S2: Twinning-maturation program, Table S3: LMD Ring Trials performed by the I-BFM FLOW network, Table S4: Table summarizing the most common problems causing incorrect MRD assessments in LMD File ring trials, Supplemental File Sl: “SOP FLOW-MRD 1.5 AIEOP-BFM”, Supplemental File S2: “SOP ALL-IC 2009”, Supplemental File S3: “Amendment to SOP ALL-IC_2014”, Supplemental File S4: “I-BFM FLOW Twinning Maturation”, Supplemental File S5: “Maturation Certificate”, Supplemental File S6: “Data Query”, Supplemental File S7: “Educational Slides”.
