# Supplementary material for: An Extensive Quality Control and Quality Assurance (QC/QA) Program Significantly Improves Inter-Laboratory Concordance Rates of Flow-Cytometric Minimal Residual Disease Assessment in Acute Lymphoblastic Leukemia: An I-BFM-FLOW-Network Report
Source: Cancers (Basel). 2021 Dec 6;13(23):6148. doi: 10.3390/cancers13236148 (PMC8656726; doi:10.3390/cancers13236148)
Supplement: Supplementary file 1 [file cancers-13-06148-s001.zip › FigureS1.pdf]

LMD MRD trials (2016-2020) BCP-Regeneration

symbol	faults	rating
green	≤ 10 %	success
yellow	≤ 25 %	warning
red	> 25 %	critical

AIEOP-BFM contributing labs

	success	warning	critical
n= 9 RT 2016	6	1	2
n= 8 RT 2017	6	2	0
n= 6 RT 2018	6	0	0
n= 7 RT 2019	5	2	0
n= 7 RT 2020	7	0	0

p= 0.57 ns (RT 2016 vs RT 2017)
p= 0.38 ns (RT 2017–RT 2020)
p= 0.32 ns (RT 2016–RT 2020)

ALL IC-BFM contributing labs

	success	warning	critical
n=17 RT 2016	9	3	5
n=16 RT 2017	13	3	0
n=16 RT 2018	12	2	2
n=17 RT 2019	14	3	0
n=17 RT 2020	14	3	0

p= 0.07 ns (RT 2016 vs RT 2017)
p= 1 ns (RT 2017–RT 2020)
p= 0.059 ns (RT 2016–RT 2020)

Total contributing I-BFM labs

	success	warning	critical
n=30 RT 2016	18	4	8
n=28 RT 2017	21	7	0
n=25 RT 2018	21	2	2
n=28 RT 2019	22	5	1
n=27 RT 2020	24	3	0

p= 0.009 (RT 2016 vs RT 2017)
p= 0.30 ns (RT 2017–RT 2020)
p=0.007 (RT 2016–RT 2020)

2x3 Contingency Table Exact test, two-tailed, Freeman-Halton extension to Fisher's exact test

LMD Trial	Group	No. of participants	Passed successfully	(%)
RT 2016	ALL IC-BFM	17	9	53
RT 2017	ALL IC-BFM	16	13	81
RT 2018	ALL IC-BFM	16	12	75
RT 2019	ALL IC-BFM	17	14	82
RT 2020	ALL IC-BFM	17	14	82
