# Supplementary material for: An Extensive Quality Control and Quality Assurance (QC/QA) Program Significantly Improves Inter-Laboratory Concordance Rates of Flow-Cytometric Minimal Residual Disease Assessment in Acute Lymphoblastic Leukemia: An I-BFM-FLOW-Network Report
Source: Cancers (Basel). 2021 Dec 6;13(23):6148. doi: 10.3390/cancers13236148 (PMC8656726; doi:10.3390/cancers13236148)

Standard Operating Procedure

≥6-color FLOW-MRD detection in ALL

AIEOP – BFM-A/G/S – CPH – INS - NSW

version "International" 1.5 of November 17, 2010

Assoc. Prof. Dr. Michael N. Dworzak (CO, PI)
BFM-A, Vienna, AUSTRIA

Prof. Dr. Giuseppe Basso (PI)
AIEOP, Padova, ITALY

Dr. Giuseppe Gaipa (PI)
AIEOP, Monza, ITALY

Dr. Richard Ratei / Dr. Leonid Karawajew (PIs)
BFM-G, Berlin, GERMANY

Assoc. Prof. Dr. Ondrej Hrusak (PI)
CLIP, Prague, CZECH REPUBLIC

Dr. Jean-Pierre Bourquin (PI)
BFM-S, Zurich, SWITZERLAND

Dr. Drorit Luria (PI)
INS, Petach-Tikva, ISRAEL

Dr. Mary Sartor (PI)
NSW, Sydney, AUSTRALIA

CONTENTS

1. Sample registration p. 3
2. Calculation of cell input p. 4
3. Determination of mAbs used in MRD monitoring p. 5
4. SYTO p. 7
5. Usage of mAbs – amounts for staining p. 8
6. Work with mAb cocktails p. 10
7. Staining and lysing of MRD samples p. 11
8. Settings and compensation p. 13
9. Acquisition of cells at diagnosis and at d15 p. 15
10. Analysis guidelines for MRD detection p. 16
11. Reporting p. 21
12. Intra-laboratory QC of cytometer performance p. 23
13. Inter-laboratory QC of analysis performance p. 24
14. Documentation and QA p. 26
15. Appendices p. 28
    - 15.1. Abbreviations p. 28
    - 15.2. Examples of center-specific set-up p. 29
        - 15.2.1. Berlin p. 29
        - 15.2.2. Monza p. 30
        - 15.2.3. Padova p. 31
        - 15.2.4. Prague p. 33
        - 15.2.5. Vienna p. 35
        - 15.2.6. Zurich p. 37
        - 15.2.7. Petach-Tikva p. 38
    - 15.3. Example of PMT-Monitoring "BD Cytometer Setup and Tracking Beads" p. 39
    - 15.4. References p. 40
    - 15.5. Examples of gating strategy for BCP- and T-ALL p. 42
    - 15.6. Examples of cross-lineage background controls p. 49

Note: **Deliverables in BLUE**

1. Sample registration

Aim: Unambiguous identification and validation of a sample submitted for immunophenotypic analysis and MRD-determination according to the needs of the clinical protocol AIEOP-BFM ALL 2008.

Procedure:

- Upon arrival of a specimen, save to documentation file at least the following information:
  - patient's name
  - date of birth
  - patient's and/or sample code (UPN, study number or similar)
  - treatment protocol
  - ALL lineage (T- or BCP-; data as available)
  - type of specimen (e.g. BM)
  - sampling time-point (within treatment protocol; e.g. at diagnosis, or day15)
  - date of sample collection
  - date of sample registration (= date of processing)

2. Calculation of cell input

Aim: For MRD detection down to the 0.01% level (with a required resolution of 10 MRD events minimum to refer a sample as positive), an optimum of 300 000 nucleated cells (not merely events, which may contain also debris etc.!) need to be acquired. Since preparation leads to cell loss it is recommended to stain at least **700 000 cells at d15**. (Diagnosis: stain 100 000, acquire 30 000 e.g.). Cell loss depends on storage and age of materials and on cell content.

Procedure:

1. Determine nucleated cell (NC) count. This can ideally (but not exclusively) be performed using an automated hemo-counter (note that this value includes also normoblasts!)
2. Calculate necessary volume by formula (see also example below):
   Input in µL = intended number of input cells/counter cell count (cells per µL)
3. round up as necessary
4. Report **NC count** to documentation file

Example:

Cell count in sample is 2100/µL (= 2.1 G/L) of PB/BM; therefore input per staining tube is 333 µL of original sample. (Formula: 700 000:2100=333)

3. Determination of mAbs used in MRD monitoring

Aim: All participants should use same combinations of essential mAbs but may use different dyes as this depends on the flow cytometer facilities available.

Procedure: The combination of mAbs per tube is based on a backbone of at least four mAbs: in **BCP-ALL** **CD19** (as primary gate), **CD10** (as immature/blast cell marker), **CD20** (for discrimination of mature B-Cells) and **CD45** (as pan leukocyte marker and quality control of BM composition); and in **T-ALL** **CD7** (as primary gate, positive in T and NK cells), **CD3** (mature T-cells), **CD5** (positive in T cells including immature, and negative in NK cells) and **CD45** (pan-leukocyte marker and quality control of BM composition).

Notably, the **expression of antigens frequently changes under treatment**. Phenotypic patterns on d15 should therefore not simply be considered similar as at diagnosis.

In analyzing the data the **primary gate** should be set in **CD19** versus SSC correlations in BCP-ALL samples, and in **CD7** versus SSC in T-ALLs, hence, it is **recommended to use similar mAb conjugates** for these two antigens **throughout all tubes**.

Essential antigens in BCP-ALL (B-I to B-III):

**CD58**	**FITC** preferred
**CD10**	**to be included in each tube**; **PE** preferred (at least in 1 tube), PE-Cy7
**CD45**	**to be included in each tube**; **PerCP** preferred, ECD
**CD19**	**to be included in each tube**; **APC** preferred, PC7
**CD20**	**to be included in each tube**; FITC, ECD, APC-Cy7, or Pacific Blue
**CD34**	PE or PE-Cy7 preferred, APC
CD38	recommended in B-I or CD10low/neg cases; FITC, Ax700
CD11a	optional; **PE only**

Essential antigens in T-ALL (T-I to T-IV):

**CD99**	FITC or PE
**sCD3**	**to be included in each tube**; PE-TR (=ECD), APC
**CD45**	**to be included in each tube**; **PerCP** preferred, ECD, APC-Cy7
**CD5**	**to be included in each tube**; **PE or PC7**
**CD7**	**to be included in each tube**; **APC** preferred, PE, PC5
**cyCD3**	PC7, Ax700
**CD4**	**essential in T-IV (mature);** FITC, Ax700
**CD8**	**essential in T-IV (mature);** PE, APC-Cy7
TdT	optional; FITC

Note: It is necessary to **use the same clone for CD3 cytoplasmatic and surface staining** in order to prevent re-staining of surface epitopes during cytoplasmic staining (after prior surface staining) because of differences in epitopes.
It is NOT necessary to engage negative control antibodies because PB and BM are sufficiently heterogeneous cell compartments, allowing for in-sample control by normal cell types which are negative for the antigens under investigation on MRD cells.

Remark: Center-specific mAb combinations, clones, and fluorochrome-conjugates are specified in Appendix 1.

QC: Centers should **keep records of specifications** of their mAbs including Lot-No., date of first usage of individual mAb vial, date of emptying, expiry date.
As **templates of normal background** each center should **acquire and retain LMD files** of at least five day 15 BM samples stained in cross-lineage set-up with the center-specific panel for BCP- as well as T-ALL (e.g. 5 BCP-follow-up samples stained with the T-panel plus 5 T-follow-up samples stained with the BCP-panel).

4. SYTO

Aim: SYTO16 and SYTO41 are DNA>RNA dyes which readily stain nucleated cells without a need for permeabilization. These dyes are used to determine all nucleated cells in samples in order to exclude non-nucleated events like erythrocytes, platelets, and debris. SYTO16 is measured in FL1-channel whereas SYTO 41 needs a violet laser for excitation. Ideally SYTO positive cells should lie in the 3rd log (not higher), to prevent overspill into other channels and to clearly separate positive from negative events.

Procedure:

SYTO 16 is delivered frozen (Invitrogen, molecular probes #S7578). The thawed stock solution can be aliquoted and refrozen, e.g. at -20°. Working solutions of SYT0 16 should be prepared as 1:1000 dilution preferably with PBS/BSA2%/0.1%NaN3 and can be stored at 4° in dark vials. This dilution is stable for months. If SYTO 16 working solution is prepared using isotonic saline, it is recommended to prepare the solution fresh every week. Add 2 µl of the 1:1000 working solution to 200 µl of sample volume, e.g. immediately before acquisition. If not bright enough add 1 further µl.

SYTO 41 is delivered frozen (Invitrogen, molecular probes #S11353) and can be handled as above, but for working solutions SYTO 41 should be diluted 1:200/250 with 20mM TRIS buffer pH7.5 and stored at 4° in dark vials. Add 3 µl to 200 µl of sample before acquisition when prepared fresh. Increase amount by 1 µl for each additional day the dilution is used.

Note: A vitality check (PI, 7AAD etc.) is NOT required because not unambiguously reflecting the in vivo situation at the time of BM/PB sampling – apoptosis may have been induced during transport and preparation. As long as scattering properties of cells are largely preserved, cells are included in MRD estimates (even if they would take up dead-cell dyes).

5. Usage of mAbs – amounts for staining

Aim: Warrant saturating staining for best resolution of blast versus normal cells.

Procedure:

General: **use amount of mAb as indicated by manufacturer**.

Note: MAb amounts as recommended on data sheets from companies are adapted for staining specimens with homogeneous cell populations at saturating conditions. If such relatively high mAb amounts are used in inhomogeneous samples containing MRD cells this may occasionally disturb phenotypic recognition. In case, mAb amounts can be optimized by titration. **If mAb amounts for usage are determined by titration, documentation of experimental evidence supporting the use of mAb at the relevant dilution must be available in the center** (and should be made available to the CO upon request). This documentation should include the type of antibody, the amount determined for usage per MRD tube, the amount recommended by the manufacturer, and a summary of the titration experiment (date, cell type, and results). If titration is used to determine the amount of mAb insert instead of the manufacturer recommendation, mind that titration is needed **with each new lot of mAb** – compare to previous lot.

Titration procedure:

For titration of ALL associated mAbs like TdT, CD5, CD7, CD10, CD19, CD20, CD34 BM or PB of leukemia initial samples with respective antigen positivity should be used (frozen samples (N2) can be used but lower fluorescence may be taken in account – viability should be ≥90%); in case a cell line is used mind the usually higher autoflourescence.

For titration of mAbs as CD3, CD4, CD8, CD11a, CD20, CD38, CD45, CD58 and CD99 PB of a healthy donor can also be used.

The amount of input of CD3 in cytoplasmatic staining must be determined separately.

Detail:

1. Stain approximately 10E-6 cells per test in a maximum of 100 µL volume.
2. Preferably use step-wise longitudinal dilution technique for staining.
3. Use a negative control (this could be cells without mAb or with matched irrelevant mouse mAbs conjugated with respective fluorochromes).
4. Use a positive control (stain with the amount of mAb indicated on bottle or data sheet.
5. Dilute mAb with PBS or just use lower input. The lowest step should be at least the tenth part of the positive control (example: positive control 20 µL; lowest titration step 2 µL).

6. Incubate, lyse, measure according to the protocol.
7. Analyze results in a histogram and compare "Median" MFIs of positive population. In case of variable positivity in non-homogeneous samples (relevant for broadly expressed antigens: CD11a, CD38, CD45, CD58, CD99), gate on distinct sub-population with highest expression.
8. It is recommended to use then the double amount of the first saturating concentration of mAb.
9. Test lot-to-lot changes using the optimal amount of mAb of the old lot as positive control, and compare the result with those from the new lot: 1x, 2x, 0.5x the amount of the old lot (example: optimum is 2 µL of old lot; check 2 µL, 4 µL and 1 µL of the new reagent); compare MFI results, make changes if necessary.
10. Save files or print outs of titrations and record expiry dates and lot numbers of mAbs.

6. Work with mAb cocktails

Aim: Working with mAb cocktails has several advantages towards economization of work and avoidance of errors: pipetting steps can be reduced and expedited, and mAb input is assured and standardized. On the other hand, since tandem fluorochrome conjugates as PE-CY7 tend to instability their inclusion into cocktails is NOT recommendable. In addition, manufacturers do not evaluate performance of their mAbs sold and stored as single reagents for performance when stored in cocktails. Therefore, accreditation of these reagents for in-vitro-diagnostics is limited to storage as recommended by company. Usage of mAb cocktails is therefore not recommended, however, it is conceded to the groups to **work with cocktails upon individual decision** as long as these are **rapidly used** and prepared fresh again (e.g. weekly) **or provided that stability is monitored**. Intra-sample plausibility

Note: Tandem conjugates must NOT be included in cocktails, except cocktails are freshly prepared each week. SYTO dyes must NOT be included in cocktails.

Procedure:
Cocktail must contain all mAb in the optimal concentration plus PBS/BSA/NaN3 (Example: 2 µL FITC+2 µL PE+1 µL PerCP+1 µL APC+4 µL PBS/BSA/NaN3). Fill-up e.g. with PBS to reach similarity of end-volumes and input-volumes between different cocktails is not necessary. After preparation, an **intra-sample plausibility check** should be performed with each new cocktail to secure that all intended mAbs are really included. A more formal cocktail check with beads is not warranted.

Note: Use cocktail according to recommended input of each single antibody (as indicated by manufacturer). In case mAb amounts for staining are determined by titration, use respective volumes: e.g. 10 µL of cocktail should contain mAbs for up to 1(-2) x10E-6 cells to be stained in a final volume of 50-100 µL. For volumes bigger than 100 µL (in low cellularity material) input of cocktail must be adjusted. This is 20 µL of cocktail for up to 200 µL end volume, and 30 µL for up to 300 µL or more.

If retained and used longer than a week, stability of cocktails must be checked (preferably at bi-weekly intervals) e.g. using BD CompBeads: for details of procedure see below (chapter on Settings and Compensation).
Save files or print outs of stability assessments and record dates of evaluation together with records of the date of cocktail preparation.

7. Staining and lysing of MRD samples

Aim: Staining for MRD assessment should be done with a stain-lyse (=red cell lysis) approach.

Procedure surface staining:

1. Add mAbs into tube (e.g. falcon BD# 352052)
2. Add BM (volume according to calculation)
3. Vortex, incubate at RT, in dark for 15 min.
4. Add 2 ml of BD lysing solution (BD# 349202 predilution 1:10 with demin. water)
5. Vortex, leave for 10 to 15 min at RT in the dark
6. Centrifuge, discard supernatant
7. Top up with washing solution, e.g. cold PBS
8. Centrifuge at 400g for 10 minutes
9. Tip off/aspirate supernatant
10. Resuspend in 200(-400) µl PBS
11. Add SYTO (see chapter SYTO)
12. Proceed to Flow Cytometer

Remarks

To steps 1 and 11:

- SYTO16 can also be added early together with mAbs (step 1), so that step 11 can be cancelled; however, SYTO41 must be added late.

To step 4:

- Add 1 ml lysis buffer, vortex, add second ml of buffer, doing so will prevent overspill of fluid.
- If incubation step 3 is performed at +4°C for some reasons, make sure that material is at room temperature before adding lysis reagent.
- 2 ml of lysing buffer are sufficient for lysing up to 500 µL of BM/PB with optimum results up to 300 µL input; in cases of >>300 µL of input, the whole volume should be split into several tubes (for example 3 tubes with 300 µL input each); add lysing solution per tube as indicated above, and blend tube contents before measurement.
- If after 10 minutes of incubation with lysing buffer a sample does not appear clear >> extend to 15 min of incubation time; in very reluctant cases (lots of red cells after step 6) one may continue at step 7 (instead of 1x PBS) with hypotonic lysis (add 1 ml of demin. water for 30 sec and then add 1 ml of double concentrated PBS).

To steps 6 to 8:

- Step 8 (second wash) can be cancelled, if step 7 is brought forward before step 6 (optional adding of extra washing solution, e.g. PBS, depends on the size of tubes used in the procedure: avoid overspill).

Procedure intracellular staining:

1. Execute point 1 to 4 from surface staining protocol
2. Add reagent A from FIX & PERM Kit (Caltag # GAS-004), increase input of reagent A according to material volume (per 50-100 µL BM staining volume add 100 µL of reagent A; increase in increments of 100 µL as necessary up to a maximum of 300 µL of reagent A)
3. Incubate for 15 min at RT in the dark
4. Top up with cold PBS
5. Centrifuge (400g 10 minutes), discard supernatant, vortex to fully resuspend the cell pellet
6. Add reagent B (similar volumes as in point 2 according to volume of resuspended pellet) and add mAbs for intracellular staining (i.e. TdT, cy-CD3)
7. Vortex and incubate 20 minutes at RT in the dark
8. Top up with cold PBS
9. Centrifuge (400g 10 minutes)
10. Adjust volume to 200(-400) µL
11. Add SYTO (see chapter SYTO)
12. Proceed to acquisition on flow cytometer.

Remarks:

See tips for surface staining, like splitting into more tubes. Hypotonic lysis may be also applied as step 8.

8. Settings and compensation

Aim: Digital flow cytometers have automated support for compensation. Automated compensation settings can also be changed manually by using bi-exponential display and/or can be overwritten with a new automatically determined compensation as necessary. As **automated compensation tools** (e.g. BD FACS DIVA CS&T module, or similar) exclude the human factor, these should be preferred. Nevertheless, to warrant that correct interpretation of phenotypic pictures is possible, it is useful to have a common approach for setting the compensation at least within participants using cytometers from the same company.

Guidelines:

General: Prepare lysed normal BM or pB sample as indicated in this SOP.

Device-specific compensation procedures must be performed by all centers.

As an example, the procedure with BD FACS DIVA is explicitely delineated below:

Procedure: Add one drop each of BD CompBeads #51-BP80212-02 (negative) and #51-BP80212-01 (anti mouse Ig–mAb catching beads) into tubes. Prepare one tube with beads only and additional tubes for the mAbs used in the MRD-panel.

Note: With NON-tandem fluorochromes it is sufficient to have one tube per fluorochrome/channel even if different antibodies are used with the respective fluorochrome. In case of tandem-fluorochrome conjugates, each Ab must be tested separately (due to changes of the individual Ab+fluorochrome with time).

Get started:

1. Add single mAb to beads suspension,
2. add 100 µl additional PBS ,
3. incubate 15 min in the dark,
4. wash and resuspend in 500 µl PBS
5. Proceed to FACS acquisition

Get started on Cytometer (BD specific):

1. Start Diva Software
2. Go to <instrument> scroll <instrument set up>, <create compensation control> now templates will open
3. define channels and in case also for additional tandem conjugates
4. Acquire lysed BM sample (not stained with mAbs!! – just to see autofluorescent background) with a gate on lymphoid cells in low speed and adjust PMT voltage to 10^2 for each fluorochrome/channel
5. Acquire unlabeled beads - file will be automatically saved; control PMT setting

6. Continue with tubes with labeled beads according to tube list - files will be automatically saved
7. When finished with acquisition go to <calculate compensation> and save and name settings (according to assay, e.g. "MRD"-settings). Settings are now stored in a separate folder, from where they can be applied anytime.

Automated compensation must be repeated (for all channels) in particular

- When PMT are changed for any reasons,
- With first usage of a new vial of tandem-conjugated AB (start with step 6 – overwrite data only of old conjugate)
- When changes in compensation are visible due to possible aging of tandem conjugates (overwrite data only of the respective conjugate)

9. Acquisition of cells at diagnosis and at d15

Aim: Adequate numbers of acquired events are a prerequisite for sufficient test sensitivity.

Guidelines:

A number of **10 cellular events (dots) of similar overall features is the minimum amount to judge a sample MRD-positive**. Hence, to warrant that a sample can reliably be judged at the threshold of 0.01%, a minimum amount of 100 000 nucleated cells should be acquired. To warrant a more reliable and reproducible test resolution, 300 000 events need to be acquired. Acquired events should contain as much as possible of relevant, i.e. nucleated events, and not residual non-lysed erythrocytes or debris (of note: in particular aplastic samples tend to poor lysis efficacy). Several actions enable reaching this goal:

- Higher cell input and acquisition (e.g. to $1x10^6$)
- Usage of hypotonic lysis (see above) in case of insufficient primary red cell lysis
- **SYTO-positive acquisition (whenever possible)**

The **most useful** and standardized of these measures is **SYTO-positive acquisition**, and this is the **method of choice if practicable** according to staining combinations used.

Acquisition at diagnosis: Acquire **30 000** events.

Acquisition at d15: Acquire **300 000 nucleated cells** (SYTO positive)

Acquisition gate must be set on SYTO-positive cells and 300 000 $SYTO^+$ events acquired. To save disk space only the SYTO-positive events may be stored.

Note: Exclude carry-over from recently run samples, e.g. by acquiring a blank sample of distilled water or sheath fluid at high rate until <300 events are acquired per minute before running a new patient specimen.

Remark:

A **SYTO16/SSC** plot is recommended for gating. However, when using SYTO41, cells with a $FSC^{high}SSC^{low/intermediate}$ pattern and lower SYTO41 uptake as other nucleated cells (with normal FSC) may appear after BD lysis: a **SYTO41/FSC** display is therefore recommended for gating, so that these cells can be included in the SYTO+ gate.

10. Analysis guidelines for MRD detection

Aim: Sensitive, specific and reproducible quantification of ALL-MRD. For FLOW-based stratification using the algorithm »**d15 BM MRD**: FLR is <0.1%, FHR is ≥10%, FMR all others« it is **necessary to be sensitive and specific at the 0.1% level**.

Procedure:

- Define primary plots (x-/y-axis): FSC/SSC, SYTO/SSCorFSC, all CDxx/SSC
- Define secondary plots (CDxx vs CDyy; recommended set-up given below; in parentheses=as available):
  - **BCP-ALL**: CD19 vs CD10
    CD58 vs CD10
    CD45 vs CD10
    CD34 vs CD10
    CD20 vs CD10
    CD38 vs CD10
    (CD11a vs CD10)
    (CD45 vs CD11a)
    (CD45 vs CD38)
  - Optional in **CD10$^{low/neg}$ BCP-ALL** CD19 vs CD38
    CD34 vs CD38
    CD10 vs CD38
    CD20 vs CD38
    CD45 vs CD38
  - **T-ALL**: sCD3 vs cyCD3
    sCD3 vs CD5
    CD7 vs CD5
    CD45 vs CD5
    CD45 vs CD99
    CD45 vs sCD3
    CD45 vs cyCD3
    CD7 vs CD99
    CD5 vs CD99
    sCD3 vs CD99
    (TdT vs CD99)
    CD4 vs CD8
- If not done already at acquisition, **gate SYTO+ events** in SYTO/SSCorFSC plot (region1) and omit SYTO^{neg} events

Note: include SYTO41^{low}FSC^{high} cells (which may appear after BD lysis) into SYTO+ compartment (SYTO/FSC plot)

- **Gate CD19+ or CD7+ cells** with **SSC$^{\text{low to intermediate}}$** (region 2)

Note: do <u>not</u> introduce an extra step for excluding doublets (e.g. via FSC-W vs FSC-A), but just exclude lymphoid-marker^{pos} cells with an aberrant SSC^{high} (in CDxx/SSC plots) or FSC^{high} profile (by back-gating).

- In all **secondary plots display in particular region 2 cells**

Note: occasionally, the CD19+ gate does not contain all CD10+SSC$^{low/int}$ cells due to very low CD19 expression! In case, adjust region 2 to include all CD10+ cells by extending region 2 into the CD19^{neg}SSC$^{low/int}$ area!

- **Gate potential MRD containing compartment among region 2** (= region 3):
  - In CD10$^{high/intermediate}$ BCP-ALL: gate **CD10+** cells
  - In CD10$^{low/negative}$ BCP-ALL: gate **CD20^{neg}** cells
  - In sCD3$^{low/negative}$ T-ALL: gate **sCD3$^{low/neg}$** cells
  - In sCD3^{high} T-ALL: gate **sCD3+** cells

- **Investigate secondary plots for leukemia-associated aberrations** and **insert sub-gate to MRD-population** if present (region 4 – MRD gate): at least one of the patterns given below will distinguish MRD cells from normal residual lymphocytes in BM at day15. »**Cluster gating«**, that is gating of cells which form a distinctive cluster based on their antigen combination patterns, **is mandatory**.
  - **CD10$^{high/intermediate}$ BCP-ALL**: on **CD10+CD19+**(/-) cells (region 3; these cells will contain mostly leukemic blasts, and possibly some mature B-cells with low CD10 expression=CD20^{high}CD45^{high})
    - confine interest to the **CD10$^{++/+++}$** cells if existing
    - check for **CD19 over-/underexpression**
    - check for **CD20 under**-/**overexpression**
    - check for **CD45 underexpression**
    - check for **CD58 overexpression**
    - check for **CD34 (over)expression**
    - check for **CD11a under**-/(over)**expression**
    - check for **CD38 underexpression**
  - **CD10$^{low/negative}$ BCP-ALL**: on **CD20^{neg}CD19+** cells (region 3; these cells will contain leukemic blasts, and possibly plasma cells=CD38^{+++})
    - confine interest to the **CD38$^{+/++}$** population if existing
    - check for **CD10 underexpression**

- check for **CD45 underexpression**
- check for **CD34 (over)expression**
- check for **CD38 underexpression**
- check for **CD58 overexpression**
- check for **CD11a under**-/(over)**expression**, if available

- **sCD3$^{low/negative}$ T-ALL**: on **sCD3$^{low/neg}$CD7+** cells (region 3; these cells will contain leukemic blasts, and normal NK cells=CD5^{neg})
  - confine interest to **cyCD3^{pos} and/or CD5^{pos}** cells if existing
  - check for **CD99 overexpression**
  - check for **TdT expression**
  - check for **CD45 underexpression**
  - check for **CD7 overexpression**
  - check for **CD5 low expression**

  Note: few cells cyCD3+sCD3$^{low/neg}$ can be seen in most BM samples (≤0.1%). These cells are also NK cells and do not express CD5 as opposed to T-ALL blasts!

  Note: cells CD99^{high}TdT^{pos}CD7^{low}sCD3^{neg} may be seen in (regenerating) BM samples. If cyCD3^{neg} these cells will rather be immature B cell precursors and should not simply be misinterpreted as T-ALL blasts! Due to residual hematopoietic function in some cases of T-ALL with incomplete marrow infiltration at diagnosis, such immature B cell precursors may be encountered even at day 15.

- **sCD3^{high} T-ALL**: on **sCD3$^{+/++}$CD7+** cells (region 3; these cells will contain leukemic blasts, and normal T cells=CD45^{++})
  - check for **sCD3 inhomogeneous expression**
  - confine interest to subfractions of sCD3$^{+/++}$population and
  - check for **CD99 overexpression**
  - check for **TdT expression**
  - check for **CD45 underexpression**
  - check for **CD7 under-/overexpression**
  - check for following aberrations **only in combination** with previous:
    - **CD5 low expression**
    - **CD4/CD8 double-positivity**
    - **CD4/CD8 double-negativity**
    - **CD4 weak expression**
    - **CD8 weak expression**

- In plot FSC/SSC (displaying region 2 only) set gate (region 5) on lymphoid cells to exclude distinct FSC$^{very\ low}$/SSC^{intermediate} events (**debris area prone to autofluorescence**) as necessary.

- In plot CD45/SSC (displaying region 1 cells) set gate (region 6) on CD45^{neg} cells. This region 6 will contain normoblasts, as well as occasionally leukemic cells. To contain only **normoblasts for quantification**, make sure that region 2 cells are then excluded from region 6.

- Make sure that population statistics is based on SYTO+ = 100%

- Display gating hierarchy and region statistics

- Save, export and print (as necessary)

- Report data of each individual tube to documentation file:
    - **Number of total acquired SYTO+ events** (report as primary value if SYTO+ acquisition is performed; can be calculated by using the following two parameters also)
    - **Number of total acquired events** (report if SYTO+ acquisition is NOT performed)
    - **% SYTO+ events among total events** (report if SYTO+ acquisition is NOT performed)
    - **MRD population in % of SYTO+** (region 4 or region 5, see above)
    - **Absolute number of MRD cells in the acquisition**
    - **MRD vote positive or negative**

      Mind that a MRD-cluster needs to contain:

      ≥10 cells with related characteristics to be regarded as **positive**

      ≥30 cells with related characteristics for **positivity "in the quantifiable range"**
    - **Normoblast population in % of SYTO+** (region 6 excluding leukemic blasts!). Note that this "scientific" parameter will be used for later retrospective evaluations only, but <u>not</u> to exclude samples for risk group assignment if certain counts are not met.

- If more than one staining/tube is used, build final MRD-estimate of the sample
    - by that tube which shows the **clearest separation of the MRD cluster** (it is not intended to build average values)

- in **T-ALL**, the value of a **surface-only tube** should be used for MRD-quantification wherever possible, whereas the permeabilized stain should be used for validation only (permeabilization may lead to skewing of BM composition).

11. Reporting

Aim: Warrant timely reporting of day15 MRD results to the clinical study office.

Procedure:

Report **within 32 hours upon receipt of BM sample (working days only), or on the first day after a weekend or holiday.**

The MRD data report to the clinical authorities should include items for unambiguous patient/sample identification and validation, as well as the Flow-MRD data including quality items. The **minimal required data set for the report includes**:

1. **Patient's name (first and family name) and/or* code (study code, UPN)**
   *as per national study group's practice
2. **Date of birth**
3. **Treatment protocol**
4. **Type of sample (e.g. bone marrow)**
5. **Time-point of assessment (e.g. day 15)**
6. **Date of sampling**
7. **Date of processing**
8. **Date of report**
9. **Signature of laboratory responsible**

10. **Sample votes** (according to the guidelines of this SOP):
    - »**MRD-Positive**«, or
    - »**MRD-Negative**«, or
    - »**Inadequate sample**«, in case, give explanation why:
        - Wrong sample (PB instead of BM, discrepancies of label to allotment)
        - Too few cells for analysis (<30 000 SYTO+ events per tube)
        - Too poor quality to be analyzed, which may be due to
            - **very significant hemodilution**
              Note: normoblasts (SYTO+CD45^{neg}lymphLIN^{neg}) are usually contained in day 15 BMs
            - **protracted time from collection to processing**
              Note: <48 hours = favorable, <96 hours = usually acceptable
            - **loss of regular scattering properties** (e.g. massive cell death)

11. **MRD-% among all nucleated cells (only if judged MRD-positive)**

12. **Interpretation of FLOW-based risk (only if NOT inadequate sample):**
   - »**FHR**« **(Flow high risk)**, i.e. MRD BM day15 ≥10% (of NC=SYTO+)
   - »**FMR**« **(Flow medium risk)**, i.e. MRD BM day15 <10%, but ≥0.1% NC
   - »**FLR**« **(Flow low risk)**, i.e. MRD BM day15 <0.1% NC
     - Note: FLOW-based risk should be interpreted only if quality requirements are fulfilled and sample is NOT rated »**inadequate**«.

Note: Reporting should be done based on the practice and choice of the clinical study authorities: e.g. ONLY to clinical study office (BFM-A and BFM-G), or to both the clinical study office AND the local treating center (AIEOP). A copy of the report must be retained in the lab.

State in clinical report: »data were generated based on the stringent quality standards developed and approved by the AIEOP-BFM FLOW-MRD group«

12. Intra-laboratory QC of cytometer performance

Aim: Secure that phenotyping results are stable over time what regards technical influences by cytometer. This is very important since expression of antigens may differ by biological causes between time-points, which should not be influenced by methodology in addition. A sequence of quality control measures should be performed in each institution/laboratory.

1. At least **yearly technical check-up** of flow cytometer by an accredited company.
2. **PMT-monitoring** by measurement of **fluorescent particles:**
    - **at least once weekly, or**
    - if low sample recruitment (<1 per week): **measure together with each MRD-sample**
    - **after technical adjustments**
3. **Once-weekly follow-up review of PMT-monitoring data**.

Procedure: Fluorescent particles should be used as useful for the different cytometer types. Notably, do not use DAKO fluorospheres or similar reagents as useful for 4-color cytometers because these beads do not allow adequate monitoring of PMTs in all channels of newer poly-color cytometers. If channel-specific PMTs are stable, bead fluorescence-peaks are visible at the same positions when followed over time. Use one reference peak throughout all experiments (preferably one out of the highest peaks).

Monitoring and adjustments: CV and position of the reference peak in follow up data must be monitored: should be similar as in start up data when using same PMT voltage. In case of continuously declining median MFI position of the reference peak in a certain channel (decline by ≥15% in log per measurement) in repetitive assessments within one month, cytometer service or adjustments must be performed in a timely manner (as appropriate).

Service and monitoring data have **to be documented in each center** and cumulative data (trend analysis) should be made available to the CO upon request:

1. n=bead assessments per year
2. n=PMT adjustments per channel per year
3. n=machinery service and adjustments per year (including date)
4. Per channel: mean of all values (per series with unchanged PMTs) of reference peak MFIs and CVs (±SD). SD should also be given in % of mean.

13. Inter-laboratory QC of analysis performance

Aim: MRD determination by Flow leads to results which should be reproducible. To achieve this aim appropriate teaching and longitudinal quality control of involved staff must be assured.

Ring test trials based on exchange of non-selected (spotted by time-point) **LMD files**, or of patient samples and spiked specimens (mixtures of leukemic cells from samples at diagnosis with normal PB or BM) are of great value to determine the quality of performance when multiple laboratories co-operate. LMD file exchange is particularly useful to assess the ability of staff in terms of post-acquisition skills, which is most crucial in MRD assessment because depending on the "human" factor in dot plot interpretation. It also shows the quality of acquired samples per center. This is specifically necessary for training of new staff and centers which start doing the MRD-assay. Technically, evaluation of exchanged LMD files can be hampered by inter-laboratory differences in the technical devices used for acquisition, which may also disturb appropriate import of files into cross-company software for analysis (e.g. BD vs DAKO; DAKO software Summit 4.3 is not compatible with BD/Coulter LMD files and vice-versa; however, **FlowJo** is compatible and could be used as common platform analysis program after an appropriate training and validation phase).
Recently, cell stabilizing reagents (e.g. TransFix® from Cytomark, distributed by Invitrogen) became available which makes **sample exchange** feasible even under consideration of several days of time lapse between sampling and analysis due to transport over boarders. Sample exchange allows in addition for assessing whether differences in sample preparation/acquisition quality exist which might impair optimal post-acquisition analysis. Commercial QC rounds with stabilized MRD-samples (4x 3 samples per year) are available from **UK NEQAS** (T:+44 114 267 3600; F: +44 114 267 3601; l.whitby@btconnect.com). A rating procedure is under development within this QC scheme. **These QC rounds will form the main basis of the AIEOP-BFM external quality control** system.

In addition to data entry to the NEQAS website (www.ukneqasli.org/SampleEntry), **results of QC rounds should be sent by each center to the CO** after closure of each trial period by NEQAS (usually 3 weeks from dispatch of samples from UK NEQAS). The CO will collect and analyze the data and communicate the joint results to all centers. **Criteria for performance evaluation** based on QC round data have been delineated in detail in Dworzak et al., Clin Cytometry (B) 2008. **Discordant results** (i.e. defined as a result different in qualitative (pos/neg) terms or divergent more than ±3x of the median [positive] value of the total group, as well as divergent with respect to stratification results as for d15 BM MRD: FLR is <0.1%, FHR is ≥10%) **will need interpretation by the center (by local PI) as well as discussion with the CO in order to take timely**

efforts to improve performance as appropriate (e.g. need for technical adjustments and/or staff education). See also p. 26.

Summary:

1. **twice yearly study group meetings** (at least once a year of <u>all</u> staff involved)
2. **web-platform** to allow LMD file exchange
3. **UK NEQAS MRD QC rounds** (4x 3 samples per year)
4. **Audit program: on-site visits** of all lab centers (at least once per center) will be performed over the active period of protocol AIEOP-BFM ALL 2009 (e.g. along with the twice-yearly rotational study group meetings or along with staff exchange, as appropriate) for review of local procedures, performance, and documentation. Audit items will be based on items in chapter »documentation and QA« (p. 25-26 of this SOP), and will also include review of data from the performance evaluation (UK NEQAS QC rounds). An **audit report** will be composed thereafter to document adherence or deviation from standards as well as the general performance of the center. Based on such an audit report individual centers will gain, if judged positive by the auditor and the CO, **accreditation** as »flow cytometry center for MRD assessment in ALL based on the stringent quality standards developed and approved by the AIEOP-BFM FLOW-MRD group«.

14. Documentation and QA

Aim: Warrant validation and traceability of data, as well as quality assurance.

Procedure:

Each center must retain appropriate and neatly arranged documentation of MRD records of each patient/sample, as well as of QC data.

MRD records should include items for unambiguous patient/sample identification and validation, as well as the Flow-MRD data including quality items. The **minimal required data set in the documentation files includes**:

1. Patient's name (first and family name)
2. Patient's code (study code, UPN)
3. Date of birth
4. Treatment protocol
5. ALL lineage
6. Time-point of assessment (e.g. day 15)
7. Type of sample (e.g. BM)
8. Date of sampling
9. Date of processing
10. Date of reporting
11. NC count of sample
12. Number of total acquired SYTO+ events (primary or calculated value, see p. 15)
13. Sample votes (positive, negative, inadequate)
14. MRD in % of SYTO+ (only if judged MRD-positive*)
15. Absolute number of MRD cells (tube with clearest separation of MRD-cluster*)
16. Normoblast population in % of SYTO+
17. Time from sampling to processing (in days, as calculated by items 8 and 9)
18. Interpretation of FLOW-based risk (FHR, FMR, FLR)
19. Copy on paper of the MRD-report sent to the clinical study center

QC/QA records should include:

1. Specifications of **mAbs** used:
    - Type, clone, label, producer, reagent number (company derived code)
    - Lot-No.
    - date of first usage of individual mAb vial
    - date of emptying
    - expiry date
    - amount used per stain (MRD tube)
    - amount recommended by manufacturer (per 1x 10^6 cells)

- titration results (as applicable; includes date of experiment, mAb specifications, type of cells, result, LMD record out-print or screen-shot)
- usage in cocktails (Yes or No; if Yes, documentation of stability)

2. Specifications of **SYTO** used:
    - Type, producer, reagent number (company derived code)
    - Lot-No.
    - date of first usage of individual vial
    - date of preparation of new working dilution
    - amount used per stain (MRD tube)

3. Automated **compensation** runs:
    - date
    - reagent (e.g. BD CompBeads)
    - type (full series or individual channel/reagent)

4. LMD files of **normal templates** (five day 15 BM examples each for the center-specific BCP- and the T-panel)

5. **Intra-laboratory longitudinal QC** data:
    - Date of cytometer service
    - Date of PMT adjustment (plus channel specification)
    - Date and consecutive number of PMT monitoring run
    - reagent (e.g. BD Cytometer Setup and Tracking Beads)
    - results of each run per channel (only for reference peak of choice)

6. **Inter-laboratory QC** data (UK NEQAS MRD QC rounds):
    - Per period of ring trial (e.g. October 2009)
    - ALL lineage per sample
    - Date of analysis (at analyzing center)
    - Results per sample:
        - sample votes
        - MRD in % of SYTO+
        - Flow-based risk estimate (as to available data)

    Performance improvement measures:

    - In case of discordant results in QC rounds, a protocol of problem interpretation including a corrected result after re-interpretation (as applicable) as well as the type and timing of measures taken (assay adjustments, staff training etc.) must be written by the local PI, and then sent and discussed with the CO. Such a protocol may be in Email format and should include pdfs of the respective discordant LMD file analysis (as necessary).

15. Appendices

15.1. Abbreviations

APC= allophycocyanin

APC-Cy7= allophycocyanin-cyanin7

BC= Beckman Coulter

BCP=B cell precursor

BD= Becton Dickinson

BM=bone marrow

CO=coordinator

cy= intracytoplasmic

CY= cascade yellow

ECD= PE-TR= phycoerythrin-Texas-Red

FITC= fluoresceinthiocynate

LMD=list mode data

mAb=monoclonal antibody

MP= Molecular Probes

MRD=minimal residual disease

PB=peripheral blood

PE= phycoerythrin

PE-Cy5= PC5= phycoerythrin-cyanin5

PE-Cy7= PC7= phycoerythrin-cyanin7

PO= pacific orange

PI=principal investigator

QC=quality control

QA= quality assurance

s= surface

15.2. Examples of center-specific set-up

15.2.1. Berlin

Flow cytometer: **CyAn ADP**, Beckman Coulter as per April 2008
3 lasers (405nm, 488nm, 635nm)

Acquisition soft ware: **Summit V4.3**, as per April 2008

Analysis soft ware: **Summit V4.3 Dako/BC**, as per April 2008
FlowJo 7.1.3 for PC

mAb-combination for BCP-ALL (B-I, B-II, B-III):

1 Tube

Laser	488nm					405nm		635nm	
FL	FITC	PE	ECD	PC5	PC7	Syto	CY	APC	APC-Cy7
CD	58	10	45	11b/38*	34	41	3	19	20
clone	AICD58	SS2/36	J.33	LS198-4-3/ Bear1	581	-	UCHT1	J4.119	L27
Company	BC	Dako	BC	BC/BC	BC	MP	Dako	BC	BD

* CD38 in case of low CD10 or pro-B ALL

mAb-combination for T-ALL:

1. Tube for immature and early T-ALL

Laser	488nm					405nm		635nm	
FL	FITC	PE	ECD	PC5	PC7	Syto	PO	APC	Alexa-700
CD	TdT	99	s3	7	5	41	45	56	cy3
clone	HT-6	3B2	UCHT 1	M-T701	B1 1a	-	HI30	NCAM16.2	UCHT1
Company	Dako	CALTAG	BC	BD	BC	MP	Caltag	BD	BD

2. Tube for mature T-ALL

Laser	488nm					405nm		635nm	
FL	FITC	PE	ECD	PC5	PC7	Syto	PO	APC	Alexa-700
CD	4	99	s3	7	5	41	8	TdT	cy3
clone	SK3	3B2	UCHT 1	M-T701	B1 1a	-	3B5	E17-1519	UCHT1
Company	BD	Caltag	BC	BD	BC	MP	Caltag	BD	BD

Monitoring of instrument performance CyAn ADP (Berlin)

Quality control of the optical alignment and of PMT-linearity is performed with Ultra rainbow calibration particles (URCPs) available from Spherotech, Inc. (Cat. No. URCP-38-2-K) according to the manufacturers instruction at:
http://www.spherotech.com/Updated%20STN%208-21-07/STN-8%20Rev%20C.pdf

15.2.2. <u>Monza</u>

Flow cytometer: **FACSCanto II Becton Dickinson**, as per April 2008
2 lasers (488, 635nm)

Acquisition soft ware: **DIVA 6.1**, as per April 2008

Analysis soft ware: **DIVA 6.1**, as per April 2008
PAG 3.0.2

<u>mAb-combination for BCP-ALL (B-I, B-II, B-III):</u>

Tube 1 (compulsory):
Syto16/CD10PE/CD45PerCP/CD34PE-Cy7/CD19APC/CD20APC-Cy7/CD38Ax700

Tube 2 (compulsory):
CD58FITC/CD11aPE/CD45PerCP/CD10PE-Cy7/CD19APC/CD20APC-Cy7/CD38Ax700

<u>List of reagents:</u>
CD10 PE (clone SS2/36, Dako)
CD10 PE-Cy7 (clone HI10a, BD)
CD19 APC (clone SJ25C1, BD)
CD45 PerCP (clone 2D1, BD)
CD20 APC-Cy7 (clone L27, BD)
CD58 FITC (clone AICD58, Coulter)
CD38 Alexa 700 (clone HIT2, EXBIO)
CD34 PE-Cy7 (clone 8G12, BD)
CD11a PE (clone LFA-1, Pharmingen BD)
SYTO16 (Molecular Probes)

<u>mAb-combination for T-ALL:</u>

Tube 1 (compulsory in immature T-ALL, but additional in mature T-ALL):
Syto16/CD99PE/sCD3PE-TR/CD45PerCP/CD5PC7/CD7APC/cyCD3 Alexa700

Tube 2 (compulsory in mature T-ALL, additional in immature T-ALL):
Syto16/CD99PE/sCD3PE-TR/CD45PerCP/CD5PC7/CD7APC/CD8APC-Cy7/CD4 Ax700

<u>List of reagents:</u>
CD99 PE (clone 3B2, Caltag)
CD3 PE-Texas Red / Alexa 700 (UCHT1, Coulter, BD)
CD5 PC7 (clone BL1a Coulter)
CD7 APC (clone 6B7, Caltag)
CD45 PerCP (clone 2D1, BD)
CD4 Alexa 700 (clone EDU-2, BD)
CD8 APC-Cy7 (clone SK1, BD)
SYTO16 (Molecular Probes)

<u>PMT-Monitoring on BD FACSCanto II (Monza):</u>
Use "BD **7-color set up beads**" (# 335775). This kit contains reagents as lyophilized pellets. Each pellet contains a mixture of four μ and 6 μ that are either unlabelled or labelled with one fluorochrome each. These beads allow monitoring instrument performance including sensitivity of the machine, as well as for prediction of loss of efficiency over time in order to make sure that technical service interventions are triggered as necessary. Values are automatically reported to data spread-sheets using the Levey-Jennings tool provided with BD FACScanto Clinical software.

15.2.3. Padova

Flow cytometer: **2 FACSCanto II Becton Dickinson**, as per April 2008
2 lasers (488, 635nm)

Acquisition and analysis soft ware: **DIVA 5.0.1,** as per April 2008
DIVA 5.0.2, as per April 2008

mAb-combination for BCP-ALL (B-I, B-II, B-III):

Tube 1 (compulsory; CD38 optional):
CD58FITC/CD10PE/CD45ECD/CD19PE-Cy7/CD34APC/CD20APC-Cy7/CD38Ax700

Tube 2 (nucleated and living cells):
Syto16/CD45 ECD/7AAD/CD19 PE-Cy7

List of reagents:
CD10 PE (ALB1 Beckman-Coulter)
CD19 PE-Cy7 (J4119 B-C)
CD45 ECD (J33 B-C)
CD20 APC-Cy7 (L27 BD)
CD34 APC (8G12 BD)
CD58 FITC (AICD58 B-C)
7AAD (B-C)
SYTO16 (Molecular Probes)
CD38 Alexa 700 (clone HIT2, EXBIO)

mAb-combination for T-ALL:

Tube 1: compulsory in immature T-ALL but additional in mature T-ALL:
CD99FITC/ CD5PE/ CD7PE-Cy5/ CD34PE-Cy7/ CD45APC/ CD3APC-Cy7

Tube 2: compulsory in mature T-ALL:
CD4FITC/ CD8PE/ CD7PE-Cy5/ CD5PE-Cy7/ CD45APC/ CD3APC-Cy7

Tube 3:
TDT FITC/CD5 PE/CD7PE-Cy5/CD45ECD/ cyCD3APC/CD3APC-Cy7

Tube 4 (nucleated and living cells):
Syto 16/ CD7PE/ CD45 ECD/7ADD

List of reagents:
CD99 FITC (Tu12 BD)
CD5 PE (L17F12 BD)
CD5 PE-Cy7 (BL1a B-C)
CD7 PE (6B7 Caltag)
CD7 PE-Cy5 (6B7 Caltag)
CD34 PE-Cy7 (8G12 BD)
CD3 APC (SK7 BD)
CD3 APC-Cy7 (SK7 BD)
CD45 APC (H130 Caltag)
CD4 FITC (SK3 BD)
CD8 PE (SK1 BD)
CD19 PE-Cy7 (J4119 B-C)
TdT FITC (HT6 B-C)

CD3 PE-Cy7 (SK7 BD)
CD45 ECD (J33 B-C)
7AAD and **SYTO16** as above

Monitoring of instrument performance

Quality control of the optical alignment and of PMT-linearity is performed with Sphero Rainbow Calibration Particles, 8-peaks, Spherotech Inc., # RFP-30-5A according to the manufacturers instructions.

15.2.4. Prague

Flow cytometer: **DAKO Cyan**, as per April 2008
3 lasers (405, 488, 635nm),
Back-up instrument: BD LSR II 3 lasers (405, 488, 635nm)

Acquisition software: **SUMMIT 4.3**, as per April 2010

Analysis software: **FlowJo 8.8.1 (MACINTOSH)**, as per April 2010

mAb-combination for BCP-ALL (B-I, B-II, B-III):
Tube 1 (compulsory):
CD20FITC/ CD10PE/ CD45PerCP/ CD34APC/ CD19PC7/CD38Ax700/SYTO41

Tube 2 (compulsory):
CD58FITC/CDxxPE/CD10ECD/CD45PerCP/CD19PC7/CD34APC/CD38Ax700/CD20PB
o **CD66c PE** if positive on ≥50% of blasts
o **CD33 PE** if positive on ≥50% of blasts
o **CD11a PE** if <50% CD66c$_{pos}$ blasts
o other relevant marker of choice

Tube 3 (nucleated and living cells):
Syto16/CD19PE/CD45 PerCP/CD71Ax700/DAPI

List of reagents:
CD10 PE (clone SS2/36, Dako)
CD10 ECD (clone ALB 1, Immunotech)
CD45 PerCp (clone 2D1, BD)
CD19 APC (clone J4.119 Immunotech)
CD19 PE (clone SJ25C, BD)
CD34 PC7 (clone LIQ, Immunotech)
CD38 Ax700 (clone HIT2, EXBIO)
CD20 FITC (clone 2H7, BD)
CD20 PB (clone 2H7, E-BIOSCIENCES)
CD58 FITC (clone AICD58, Immunotech)
CD66c PE (clone KOR-SA 3544, Immunotech)
CD11a PE (clone LFA-1, Pharmingen BD)
CD71 Ax700 (clone MEM-75, EXBIO)
SYTO16 (Molecular probes)
SYTO41 (Molecular probes)
DAPI (Molecular probes)

mAb-combination for T-ALL:
Tube 1 (compulsory in immature T-ALL but additional in mature T-ALL):
TdTFITC/CD99PE/sCD3PE-TR/CD45PerCP/CD5PC7/CD7APC/cyCD3Ax700/SYTO41

Tube 2 (compulsory in immature T-ALL but additional in mature T-ALL):
TdTFITC/CD5PE/CD7 ECD/CD45PerCP/CD3PC7/intra-CD3 APC/-- /SYTO41

Tube 3 (compulsory in mature T-ALL, additional in immature T-ALL):
CD4FITC/CD99PE/sCD3PE-TR/CD45PerCP/CD5PC7/CD7APC/CD8Ax700/SYTO41

List of reagents:
TdT FITC (clone HTdT6 Dako)
CD99 PE (clone 3B2 Caltag)
CD3 PE-Texas Red and Alexa 700 (UCHT1, BD)
CD3 PE-Cy7 and APC (UCHT1, Coulter)
CD5 PE and CD5 PC7 (clone BL1a, Coulter)

CD7 APC (clone 6B7, Caltag)
CD45 PerCP (clone 2D1, BD)
CD4 FITC (clone SK3, BD)
CD8 Ax700 (clone SK1, BD)
SYTO41 (Molecular Probes)

PMT-Monitoring on DAKO Cyan:
Use Spherotech rainbow calibration particles (8-peaks): # RCP-50-5A
Daily procedure before measurement of clinical samples:

1. One drop of Rainbow particles into 1 ml PBS, shake properly
2. open the panel called "Rainbow"
3. removal of the tube with DI water
4. automatic back-flush
5. insertion of the tube with rainbow particles
6. measurement on low pressure of 10 000 events
7. removal of the tube, closing the lever
8. gates adjustment and recording of the CV of the 8th peak into Excel-file
9. save the LMD data-file with the name RAINBOW Crrmmdd

Calculation of cell-input:
Instead of assessing nucleated cell counts on hemo-counters, counting is based on event counts per second when acquiring samples at lowest flow on the cytometer (a fixed sample volume is aspired per second and acquired events will be 100% of throughput at lowest flow rate). Measured flow rate can then be compared with standard values derived from samples with known cellularity in order to extrapolate the respective nucleated cell count.

1. Take 5 µL of sample and add 250 µL of lysing solution (NH_4Cl)
2. Incubate for 15 minutes, no wash
3. Acquire with standard speed of sheath fluid
4. Measure flow rate of non-debris events
5. Input flow rate into formula which includes flow rate of samples with known cell count

15.2.5. Vienna

Flow cytometer: **LSR II Becton Dickinson**, as per April 2008
3 lasers (405, 488, 635nm)

Acquisition soft ware: **DIVA 6.1.2**, as per January 2009

Analysis soft ware: **DIVA 6.1.2**, as per January 2009
PAG 3.0.2

mAb-combination for BCP-ALL (B-I, B-II, B-III):

Tube 1 (compulsory):
CD20FITC/CD10PE/CD45PerCP/CD34PECy7/CD19APC/CD38Ax700/SYTO41

Tube 2 (compulsory):
CD58FITC/CD11aPE/CD45PerCP/CD10PECy7/CD19APC/CD20APCCy7/CD38Ax700/SYTO41

List of reagents:
CD10 PE (clone SS2/36, Dako)
CD10 PE-Cy7 (clone HI10a, BD)
CD19 APC (clone SJ25C1, BD)
CD45 PerCP (clone 2D1, BD)
CD20 FITC; APC-Cy7 (clone L27, BD)
CD58 FITC (clone AICD58, Coulter)
CD38 Ax700 (clone HIT2, EXBIO)
CD34 PE; CD34 PE-Cy7 (clone 8G12, BD)
CD11a PE (clone LFA-1, Pharmingen BD)
SYTO41 (Molecular Probes)

mAb-combination for T-ALL:

Tube 1 (compulsory in immature T-ALL but additional in mature T-ALL):
TdTFITC/CD99PE/sCD3PE-TR/CD45PerCP/CD5PC7/CD7APC/cyCD3Ax700/SYTO41

Tube 2 (compulsory in mature T-ALL, additional in immature T-ALL):
CD4FITC/CD99PE/sCD3PE-TR/CD45PerCP/CD5PC7/CD7APC/CD8APC-Cy7/SYTO41

List of reagents:
CD99 PE (clone 3B2 Caltag)
TdT FITC (clone HTdT6 Dako, Caltag)
CD3 PE-Texas Red and Alexa 700 (UCHT1, BD)
CD5 PC7 (clone BL1a, Coulter)
CD7 APC (clone 6B7, Caltag)
CD45 PerCP (clone 2D1, BD)
CD4 FITC (clone SK3, BD)
CD8 APC-Cy7 (clone SK1, BD)
SYTO41 (Molecular Probes)

PMT-Monitoring on BD LSRII:
Use **"BD Cytometer Setup and Tracking Beads" (# 641319**; see Appendix 2). This reagent contains fluorescent beads which give three peaks (low, medium, high) in each fluorescence channel. **BD FACSDiva software 6.0 or later** allows the software even to automatically characterize, track, and report measurements of the # 641319 beads when measured on supported BD digital flow cytometers (refer to BD Cytometer Setup and Tracking Application Guide for set up information). Beads (#641319) are prepared according instructions; **measurement is performed weekly**. Results of each

measurement are saved automatically and can be exported for continuous reporting. With software 6.1.2 also automated compensation can be performed with beads or real cells. Cells are preferred because providing more accurate compensation. All QC/QA steps related to PMT-monitoring and compensation on LSR II are regulated through internal SOP **"FI-GE-002 LSR II Durchflusszytometer"**.

15.2.6. Zurich

Flow cytometer: FACSCanto II Becton Dickinson
3 lasers (405nm, 488nm, 633nm)

Acquisition software: BD FACSDiva Software v6.1.2

Analysis software: BD FACSDiva Software v6.1.2

mAb-combination for BCP-ALL (B-I, B-II, BIII):

Tube	Laser	405nm		488nm				633nm	
	FL	SYTO	PO	FITC	PE	PerCP-Cy5.5	PE-Cy7	APC	APC-H7
	CD	41	45	58	11a	34	19	10	20
	Clone	-	HI30	AICD58	HI111	8G12	J3-119	HI10a	L27
1	Company	Molecular Probes	Invitrogen	BC	BD Pharmingen	BD	BC	BD	BD
	FL	SYTO	PO	FITC	PE	PerCP-Cy5.5	PE-Cy7	APC	APC-H7
	CD	41	45	38	11b	34	19	10	20
	Clone	-	HI30	HIT2	D12	8G12	J3-119	HI10a	L27
2	Company	Molecular Probes	Invitrogen	BioLegend	BD	BD	BC	BD	BD

Tube 1
CD58FITC/CD11aPE/CD34PerCP-Cy5.5/CD19PE-Cy7/CD10APC/CD20APC-H7/CD45PO/SYTO41

Tube 2
CD38FITC/CD11bPE/CD34PerCP-Cy5.5/CD19PE-Cy7/CD10APC/CD20APC-H7/CD45PO/SYTO41

List of reagents:
CD10 APC (HI10a, BD)
CD11a PE (HI111, BD Pharmingen)
CD11b PE (D12, BD)
CD19 PE-Cy7 (J3-119, BC)
CD20 APC-H7 (L27, BD)
CD34 PerCP-Cy5.5 (8G12, BD)
CD38 FITC (HIT2, BioLegend)
CD58 FITC (AICD58, BC)
CD45 PO (HI30, Invitrogen)
SYTO41 (Molecular Probes)

mAb-combinatin for T-ALL:

Tube	Laser	405nm		488nm				633nm	
	FL	SYTO	PO	FITC	PE	PerCP-Cy5.5	PE-Cy7	APC	APC-H7
	CD	41	45	nuTdT	99	cy3	5	7	sm3
	Clone	-	HI30	HT6	TÜ12	SK7	BL1a	124-1D1	SK7
1	Company	Molecular Probes	Invitrogen	Dako	BD Pharmingen	BD	BC	eBiosciences	BD
	FL	SYTO	PO	FITC	PE	PerCP-Cy5.5	PE-Cy7	APC	APC-H7
	CD	41	45	8	99	sm3	5	7	4
	Clone	-	HI30	SK1	TÜ12	SK7	BL1a	124-1D1	SK3
2	Company	Molecular Probes	Invitrogen	BD	BD Pharmingen	BD	BC	eBiosciences	BD

Tube 1
nuTdTFITC/CD99PE/cyCD3PerCP-Cy5.5/CD5PE-Cy7/CD7APC/smCD3APC-H7/CD45PO/SYTO41

Tube 2
CD8FITC/CD99PE/smCD3PerCP-Cy5.5/CD5PE-Cy7/CD7APC/CD4APC-H7/CD45PO/SYTO41

List of reagents:
CD3 PerCP-Cy5.5 (SK7, BD)
CD3 APC-H7 (SK7, BD)
CD4 APC-H7 (SK3, BD)
CD5 PE-Cy7 (BL1a, BC)
CD7 APC (124-1D1, eBiosciences)
CD8 FITC (SK1, BD)
CD45 PO (HI30, Invitrogen)
CD99 PE (TÜ12, BD Pharmingen)
TdT FITC (HT6, Dako)
SYTO41 (Molecular Probes)

PMT-Monitoring on BD FACSCanto II:
Use "BD Cytometer Setup and Tracking Beads" (# 641319 = one 3 mL vial / # 642412 = three 3 mL vials). Each vial contains equal concentrations of polystyrene beads of three fluorescence emission intensities (dim, midrange and bright) in each fluorescence channel. The CS&T-Beads allow the software to automatically characterize, track and report measurements of supported BD digital flow cytometers (refer to BD Cytometer Setup and Tracking Application Guide for setup information).

Preparation of beads according to the manufacturer's instructions at:
http://www.bdbiosciences.com/external_files/is/doc/tds/Package_Inserts_RUO/live/web_enabled/23-9141-01_CS&T%20Beads_PI_RUO.pdf

15.2.7. <u>Petach-Tikva</u>

Flow cytometer: **FACSCanto II Becton Dickinson**, as per February 2009
3 lasers (405nm, 488 nm, 635nm)

Acquisition software: **DIVA 6.1.2**
Analysis software: **DIVA 6.1.2**

mAb-combination for BCP-ALL (B-I, B-II, B-III)

Tube 1
CD58FITC/CD10PE/CD34PerCPcy5.5/CD38PC7/CD19APC/CD20APC-H7/CD45PO/SYTO41

Tube 2
CD38FITC/CD11aPE/CD34PerCPcy5.5/CD10PC7/CD19APC/CD20APCH7/CD45PO/SYTO41

<u>List of reagents:</u>
CD10 PEcy7 (HI10a BD)
CD10 PE (SS2/36 Dako)
CD19 APC (SJ25C1 BD)
CD20 APC-H7 (L27 BD)
CD58 FITC (AICD58 Coulter)
CD38 FITC (HIT2 BD)
CD38 PEcy7 (HIT2 BD)
CD34 PerCPcy5.5 (8G12 BD)
CD11a PE (LFA-1 Pharmingen BD)
CD45 Pacific Orange (HI30 Invitrogen)
SYTO41 (Invitrogen/Molecular Probes)

mAb-combination for T-ALL:

Tube 1
TdTFITC/CD99PE/cyCD3PerCP-Cy5.5/CD5PC7/CD7APC/sCD3APC-H7/CD45PO/SYTO41

Tube 2
CD4FITC/CD99PE/CD8PerCP-Cy5.5/CD5PC7/CD7APC/sCD3APC-H7/CD45PO/SYTO41

<u>List of reagents:</u>
CD99 PE (3B2 Caltag)
TdT FITC (HTdT6 Dako)
CD3 APC-H7 AND PerCPcy5.5 (SK7 BD)
CD5 PC7 (BL1a Coulter)
CD7 APC (6B7 Caltag)
CD4 FITC (SK3 BD)
CD8 PerCPcy5.5 (SK1 BD)
CD45 PO (HI30 Invitrogen)
SYTO41 (Invitrogen/Molecular Probes)

<u>PMT-Monitoring on BD Canto II:</u>
Use BD Cytometer Setup and Tracking Beads (# 641319). Each vial contains equal concentrations of beads of three fluorescence emission intensities (bright, mid and dim) in each fluorescence channel. The beads allow the software to automatically characterize, track, and report measurements of supported BD digital flow cytometrs.
Defining baseline: Add to 0.5 mL diluent 3 drops of beads. Vortex and proceed using CST features in the Cytometer menu. Daily performance Checks: Add 1 drop of beads to 0.35 mL diluents, vortex and run with CST features using performance setup. Performance check settings are automatically entered into Levey-Jenning plots for performance tracking.

15.3. Example of PMT-Monitoring

PMT-Monitoring by
BD Cytometer Setup and Tracking Beads
(# 641319)

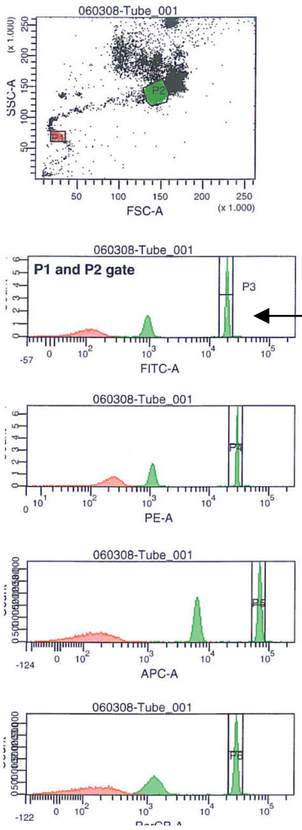

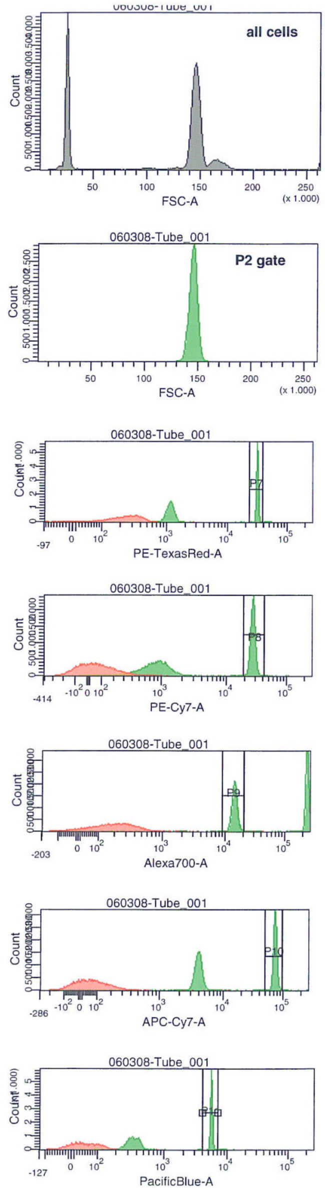

Specimen Name: 060308
Record Date: 06.03.2008 12:23:47

Population	FITC-A CV	PE-A CV	PE-Tex... CV	PerCP-A CV	PE-Cy7-A CV	APC-A CV	Alexa7... CV	APC-Cy... CV	PacificB... CV
FITC	3,1	2,3	3,1	6,1	9,8	6,1	5,3	7,8	3,1
PE	3,1	2,3	3,1	6,2	10,1	6,2	5,2	8,1	3,4
APC	3,3	2,7	3,4	6,2	9,7	6,1	5,1	7,7	3,6
PerCP	3,1	2,4	3,1	6,1	9,7	6,1	5,2	7,7	3,2
PE- Texas Red	3,1	2,3	3,1	6,2	10,1	6,2	5,2	8,1	3,1
PE-Cy7	3,5	2,9	3,6	6,2	9,0	6,2	4,9	7,1	3,8
Alexa 700	12,2	11,1	13,9	26,7	42,9	11,2	10,0	13,1	18,2
APC-Cy7	3,5	2,9	3,6	6,2	9,0	6,1	4,9	6,8	3,8
PacificBlue	3,1	2,3	3,1	6,1	9,8	6,1	5,3	7,8	3,1

Specimen Name: 060308
Record Date: 06.03.2008 12:23:47

Population	FITC-A Median	PE-A Median	PE-Tex... Median	PerCP-A Median	PE-Cy7-A Median	APC-A Median	Alexa70... Median	APC-Cy... Median	PacificB... Median
FITC	19.076	28.320	30.487	27.804	26.390	68.162	234.254	71.695	5.435
PE	19.076	28.320	30.487	27.805	26.390	68.163	234.256	71.696	5.435
APC	19.077	28.321	30.488	27.806	26.393	68.171	234.270	71.701	5.435
PerCP	19.076	28.320	30.487	27.805	26.391	68.164	234.256	71.697	5.435
PE- Texas Red	19.076	28.320	30.487	27.805	26.390	68.163	234.256	71.696	5.435
PE-Cy7	19.078	28.323	30.490	27.806	26.396	68.166	234.275	71.703	5.435
Alexa 700	914	1.081	1.135	1.220	813	6.148	14.262	3.832	347
APC-Cy7	19.078	28.324	30.494	27.805	26.390	68.162	234.252	71.695	5.435
PacificBlue	19.076	28.320	30.487	27.804	26.390	68.161	234.256	71.696	5.435

Tube: Tube_001

Population	#Events	%Parent	%Total
All Events	50.000		100,0
P1	15.562	31,1	31,1
P2	26.290	52,6	52,6
FITC	12.969	49,3	25,9
PE	12.970	49,3	25,9
APC	12.977	49,4	26,0
PerCP	12.969	49,3	25,9
PE- Texas Red	12.968	49,3	25,9
PE-Cy7	12.910	49,1	25,8
Alexa 700	13.045	49,6	26,1
APC-Cy7	12.909	49,1	25,8
PacificBlue	12.967	49,3	25,9

FITC-population refers to beads defined by bars in FITC-histogram: same population assessed in all other

Specimen Name: 060308
Record Date: 06.03.2008 12:23:47

Population	FITC-A CV	PE-A CV	PE-Texa... CV	PerCP-A CV	PE-Cy7-A CV	APC-A CV	Alexa70... CV	APC-Cy... CV	PacificBl... CV
FITC	3,1	2,3	3,1	6,1	9,8	6,1	5,3	7,8	3,1

15.4. References

1. Dworzak MN, Fritsch G, Fleischer C, et al. Multiparameter phenotype mapping of normal and post-chemotherapy B lymphopoiesis in pediatric bone marrow. Leukemia. 1997;11:1266–1273.

2. Dworzak MN, Fritsch G, Fleischer C, et al. Comparative phenotype mapping of normal vs. malignant pediatric B-lymphopoiesis unveils leukemia-associated aberrations. Exp Hematol. 1998;26:305-313.

3. Dworzak MN, Fröschl G, Printz D, et al. Prognostic significance and modalities of flow cytometric minimal residual disease detection in childhood acute lymphoblastic leukemia. Blood. 2002;99:1952-1958.

4. Veltroni M, De Zen L, Sanzari MC, et al. Expression of CD58 in normal, regenerating and leukemic bone marrow B-cells: implications for the detection of minimal residual disease in acute lymphocytic leukemia. Haematologica. 2003;11:1245–1252.

5. Dworzak MN, Fröschl G, Printz D, et al. CD99 expression in T-lineage ALL: implications for flow cytometric detection of minimal residual disease. Leukemia. 2004;18:703-708.

6. Gaipa G, Basso G, Maglia O, et al. Drug-induced immunophenotypic modulation in childhood ALL: implications for minimal residual disease detection. Leukemia. 2005;19:49-56.

7. Gaipa G, Basso G, Aliprandi S, et al. Prednisone induces immunophenotypic modulation of CD10 and CD34 in non-apoptotic B-cell precursor acute lymphoblastic leukemia cells. Cytometry B (Clin Cytometry). 2008;74:150-155.

8. Dworzak MN, Gaipa G, Veltroni M, et al. Standardization of flow cytometric minimal residual disease evaluation in acute lymphoblastic leukemia: Multi-centric assessment is feasible. Results of the AIEOP-BFM-ALL-FCM-MRD-Study Group. Cytometry B (Clin Cytometry). 2008:74:331-340.

9. Dworzak M.N., Schumich A., Printz D., Pötschger U., Husak Z., Attarbaschi A., Basso G., Gaipa G., Ratei R., Mann G., Gadner H. for the AIEOP-BFM-ALL-FCM-MRD-Study Group. CD20 up-regulation in pediatric B-cell precursor acute lymphoblastic

leukemia during induction treatment: setting the stage for anti-CD20 directed immunotherapy. Blood. 2008:112:3982-3988

10. Ratei R., Basso G., Dworzak M., Gaipa G., Veltroni M., Rhein P., Biondi A., Schrappe M., Ludwig W.D., Karawajew L. Monitoring treatment response of childhood precursor B-cell acute lymphoblastic leukemia in the AIEOP-BFM-ALL 2000 protocol with multiparameter flow cytometry: predictive impact of early blast reduction on the remission status after induction. Leukemia. 2009:23(3):528-534.

11. Basso G., Veltroni M., Valsecchi M.G., Dworzak M., Ratei R., Silvestri D., Benetello A., Buldini B., Maglia O., Masera G., Conter V., Arico M., Biondi A., and Gaipa G. (2009) Risk Of Relapse Of Childhood Acute Lymphoblastic Leukemia Is Predicted By Flow Cytometric Measurement Of Residual Disease On Day 15 Bone Marrow. Journal of Clinical Oncology 27(31): 5168-5174

12. Brüggemann M., Schrauder A., Raff T., Pfeifer H., Dworzak M., Ottmann O.G., Asnafi V., Baruchel A., Bassan R., Benoit Y., Biondi A., Cavé H., Dombret H., Fielding A.K., Foà R., Gökbuget N., Goldstone A.H., Goulden N., Henze G., Hoelzer D., Janka-Schaub G.E., Macintyre E.A., Pieters R., Rambaldi A., Ribera J.M., Schmiegelow K., Spinelli O., Stary J., von Stackelberg A., Kneba M., Schrappe M., van Dongen J.J. (2010) Standardized MRD quantification in European ALL trials: Proceedings of the Second International Symposium on MRD assessment in Kiel, Germany, 18-20 September 2008. Leukemia 24(3): 521-535

13. Rhein P., Mitlohner R., Basso G., Gaipa G., Dworzak M. N., Kirschner-Schwabe R., Hagemeier C., Stanulla M., Schrappe M., Ludwig W.-D., Karawajew L., and Ratei R. (2010) CD11b is a therapy-resistance and minimal residual disease-specific marker in precursor B-cell acute lymphoblastic leukemia. Blood 115(18): 3763-3771

14. Dworzak M.N., Gaipa G., Schumich A., Maglia O., Ratei R., Veltroni M., Husak Z., Basso G., Karawajew L., Gadner H., Biondi A. (2010) Modulation of antigen expression in B-cell precursor acute lymphoblastic leukemia during induction therapy is partly transient: Evidence for a drug-induced regulatory phenomenon. Results of the AIEOP-BFM-ALL-FLOW-MRD-Study Group. Cytometry B Clin Cytom. 78(3): 147-153

15.5. Examples of center-specific gating strategy

15.5.1. Berlin

9 – color analysis protocol for BCP-ALL (Berlin)

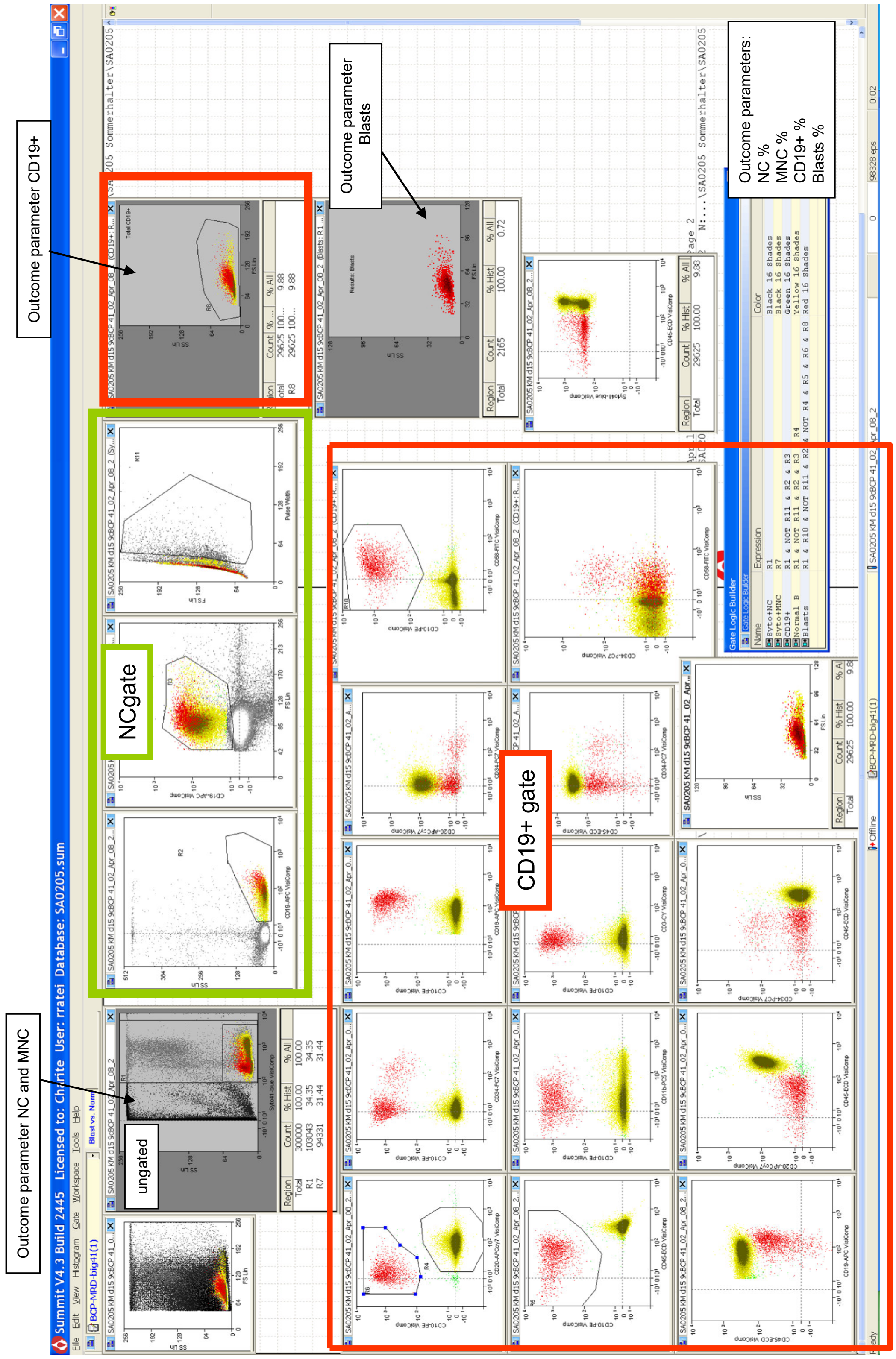

Protocol screenshot from Monitor HPLP3065: 2560x1600Pixel, 32“

15.5.2. Monza

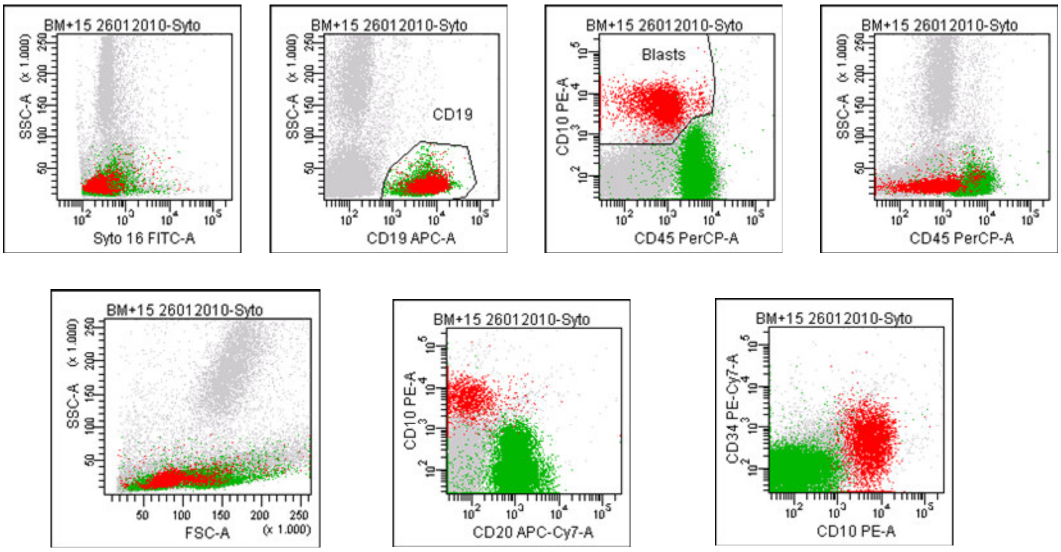

Tube: ALL-BCP

Population
- Syto 16 positive
  - CD19
    - Blasts

2,5% blasts

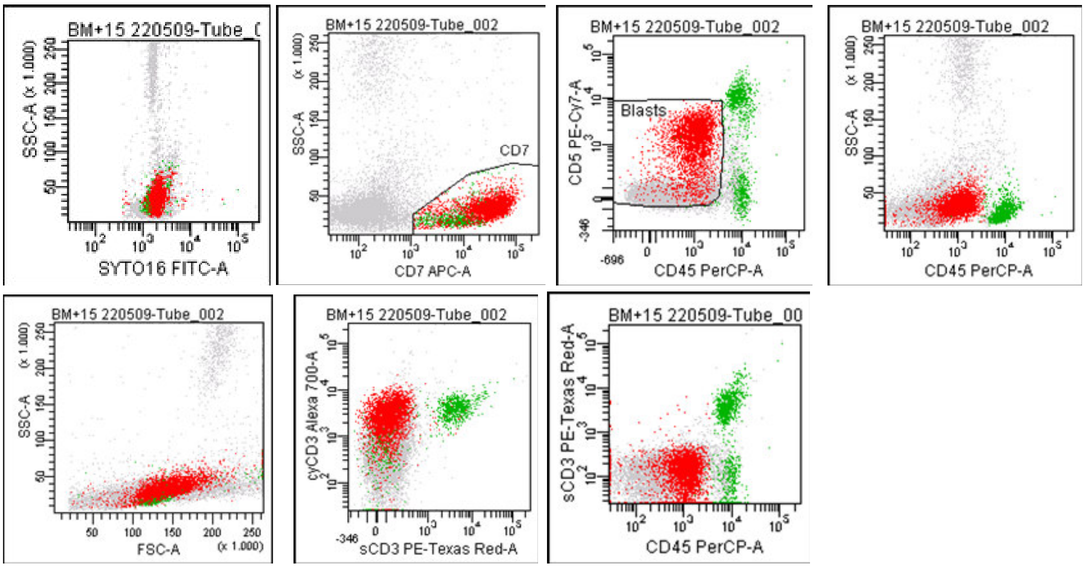

Tube: ALL-TCP

Population
- Syto 16
  - CD7
    - Blasts

20% blasts

15.5.3. Padova

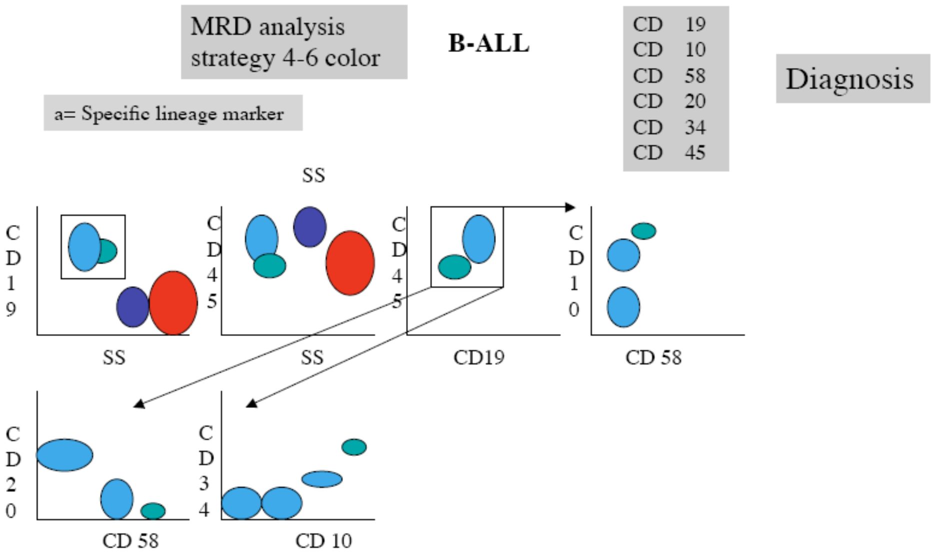
MRD analysis
strategy 4-6 color
B-ALL
CD 19
CD 10
CD 58
CD 20
CD 34
CD 45
Diagnosis
a= Specific lineage marker
SS
CD19
CD45
CD10
CD20
CD34
SS
SS
CD19
CD 58
CD 58
CD 10

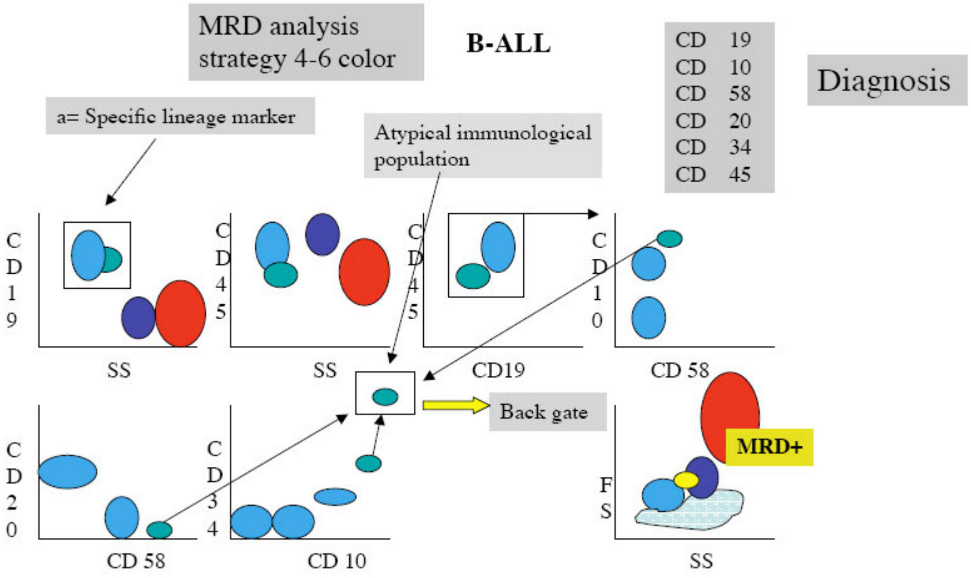
MRD analysis
strategy 4-6 color
B-ALL
CD 19
CD 10
CD 58
CD 20
CD 34
CD 45
Diagnosis
a= Specific lineage marker
Atypical immunological
population
CD19
CD45
CD10
CD20
CD34
FS
SS
SS
CD19
CD 58
Back gate
MRD+
CD 58
CD 10
SS

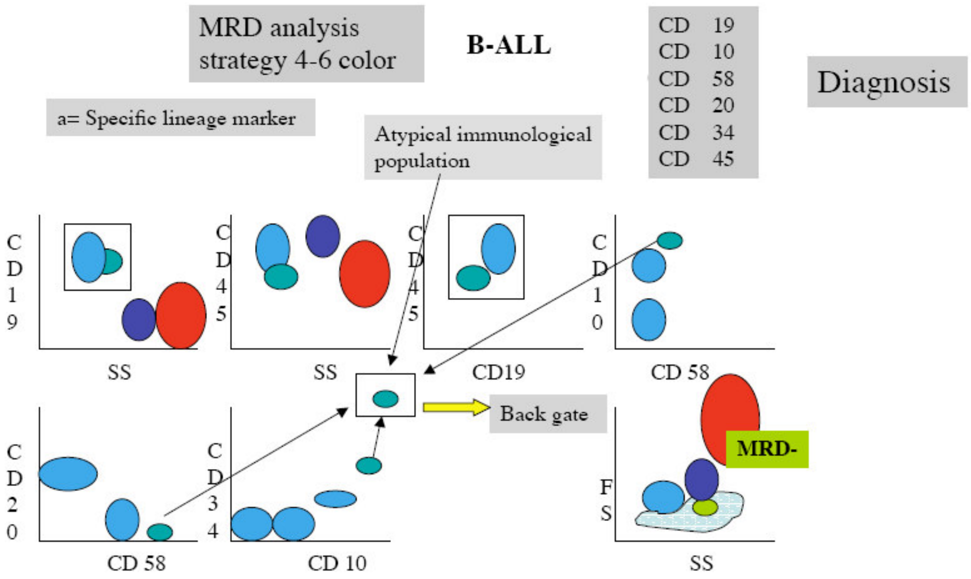
MRD analysis
strategy 4-6 color
B-ALL
CD 19
CD 10
CD 58
CD 20
CD 34
CD 45
Diagnosis
a= Specific lineage marker
Atypical immunological
population
CD19
CD45
CD10
CD20
CD34
FS
SS
SS
CD19
CD 58
Back gate
MRD-
CD 58
CD 10
SS

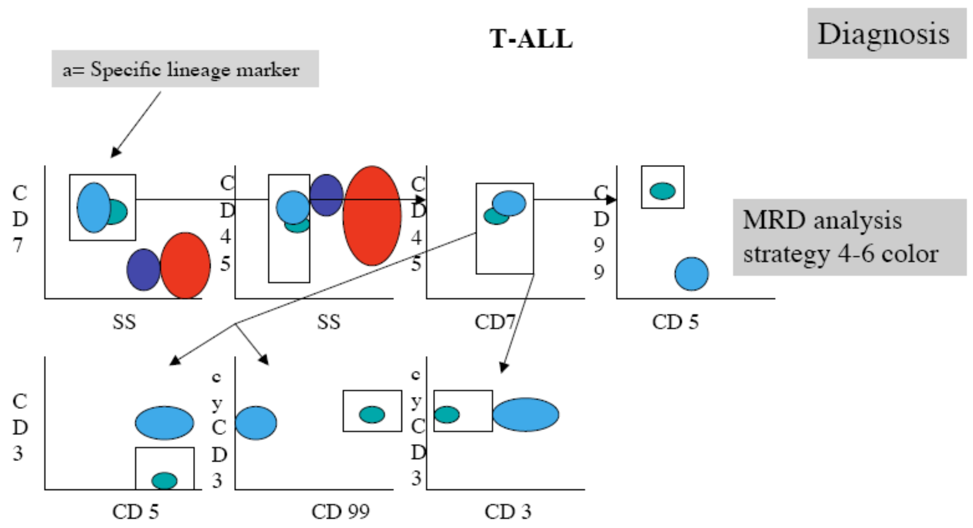

T-ALL

T-ALL	CD 7<br>CD 99<br>CD 5<br>CD 3<br>CD cy3

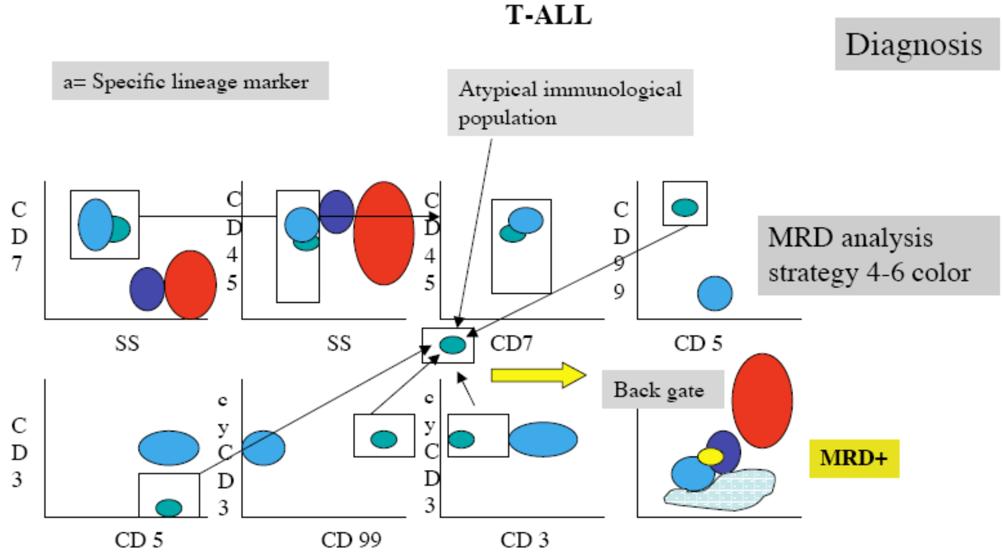

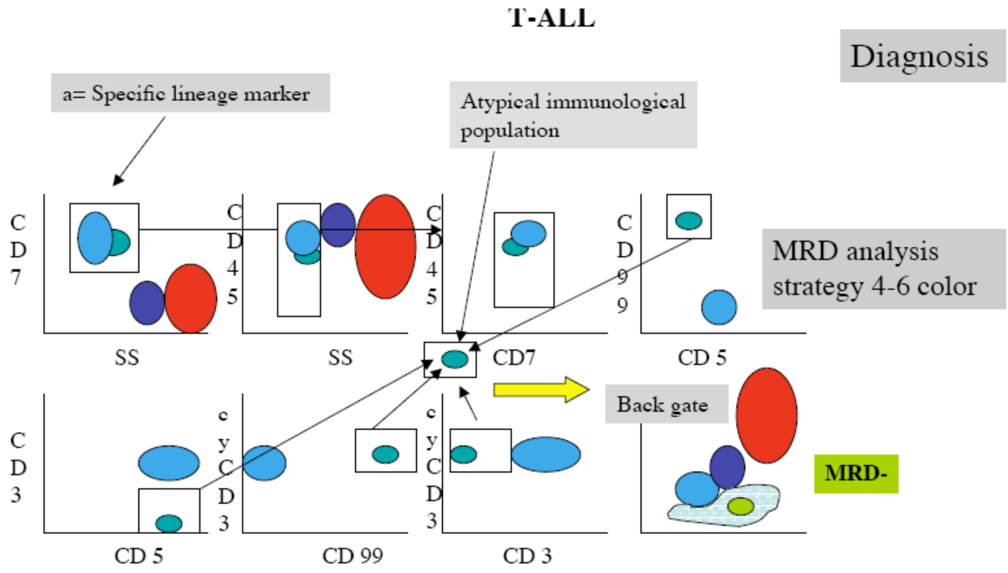

15.5.4. Prague

Interpretation procedure

We use a list of predefined gates which have to be checked in a predefined workspace.

In **tube 2**, all predefined subpopulations are visually checked, a cluster which is covering blast subpopulation is manually gated, and the gate is then called "suspect blasts". In layout editor these cells are red, all graphs are checked. If there are some clear non-specific events, a second gate can be defined which is called "suspect blasts refined" (than the final MRD population is green).
Tube 1: also predefined subpopulations are analyzed, blast are calculated directly from SYTO41 positive cells. Higher value out of tube 1 or 2 is reported as MRD. Complete workspace, gating strategy, pdf files from the analysis are stored in every patient.
Tube 3: SYTO16: % of all B cells is calculated, quality of the samples regarding nucleated erythroid cells is assessed (CD45negCD19neg and CD71++CD45negCD19neg).

Example BCP ALL
Tube 1.

Σ Count	534443	
SYTO41+	46.9	250593
Σ Count	250593	
19-45-	10.2	25454
Σ Count	25454	
CD19+	14.2	35585
Σ Count	35585	
CD34+	0.14	49
Σ Count	49	
Σ Freq. of Grandparent	0.02	
CD10+MATURE B CELLS EXCLUDED	0.33	116
Σ Count	116	
Σ Freq. of Grandparent	0.046	
CD10+34+	0.12	41
Σ Count	41	
Σ Freq. of Grandparent	0.016	
CD10negCD20neg	0.35	124
Σ Count	124	
Σ Freq. of Grandparent	0.049	
MATURE B CELLS	97.8	34807
Σ Count	34807	
Σ Freq. of Grandparent	13.9	

Tube 2.

WIDE LYMO	82	609344
Σ Count	609344	
10+	7.88	48027
19+20+	31.1	189500
19+58++	4.3	26195
19+66+	2.83	17266
34+	48.8	297167
45++	36.1	219913
45dim	49.4	301102
CD19+	54.9	334572
Σ Count	334573	
10++58++	3.89...	13
Σ Count	13	
10+38dim neg	2.64	8844
Σ Count	8844	
10+58++	7.13	23864
Σ Count	23864	
34+	87.5	292809
Σ Count	292809	
34+10+	0.25	839
Σ Count	839	
58++66c neg	56.5	189117
Σ Count	189117	
58++66c+	1.83	6108
Σ Count	6108	
CD10+MATURE B CELLS EXCLUDED	6.65	22237
Σ Count	22237	
CD10+66C+	0.31	1034
Σ Count	1034	
CD10NegCD20neg	16.1	53877
Σ Count	53877	
CD34NEG38++	0.15	79
Σ Count	79	
Σ Freq. of Grandparent	0.024	
MATURE B CELLS	57.5	192497
Σ Count	192497	
SUSP BLASTS	87.4	292546

Tube 3.

Σ Count	226171	
CD45DIM GRANULO	18.7	42346
DAPI NEG	4.96	11207
SYTo16+	47.3	106989
Σ Count	106989	
CD19+	13.2	14109
Σ Count	14109	
WIDE LYMO	100	14105
Σ Count	14105	
Σ Freq. of Grandparent	13.2	
CD19+45DIM OR NEG	0.13	138
Σ Count	138	
WIDE LYMO	94.9	131
Σ Count	131	
Σ Freq. of Grandparent	0.12	
CD19-45-	2.69	2875
Σ Count	2875	
CD71++	2.43	70
Σ Freq. of Grandparent	0.065	
DAPI NEG	10.3	11007
Σ Count	11007	

Σ Count	226171	
CD45DIM GRANULO	18.7	42346
DAPI NEG	4.96	11207
SYTo16+	47.3	106989
Σ Count	106989	
CD19+	13.2	14109
Σ Count	14109	
WIDE LYMO	100	14105
Σ Count	14105	
Σ Freq. of Grandparent	13.2	
CD19+45DIM OR NEG	0.13	138
Σ Count	138	
WIDE LYMO	94.9	131
Σ Count	131	
Σ Freq. of Grandparent	0.12	
CD19-45-	2.69	2875
Σ Count	2875	
CD71++	2.43	70
Σ Freq. of Grandparent	0.065	
DAPI NEG	10.3	11007
Σ Count	11007	

example T ALL

Tube 1.

BLASTY	69.3	91061
CD7+	41.8	38106
CD7+5+	91.6	34893
CD3neg	1.3	452
CD99++	0.5	174
sy41+	72.3	94982
BLASTY	60.2	57225
CD7+	66.3	37941
CD7+5+	91.7	34790
CD3neg	1.18	409
Σ Freq. of CD7+	1.08	
Σ Freq. of sy41+	0.43	
CD99++	0.5	174
Σ Freq. of CD7+	0.46	
Σ Freq. of sy41+	0.18	

Tube 2.

syto41	88.6	163175
blasty	59.2	96600
cd7+5+	40.2	38881
cd99++	0.34	132
CD99+az++3neg	0.92	356
Σ Freq. of syto41	0.22	
Σ Freq. of Total	0.19	
non malignant T cells	96.1	37370
Σ Freq. of syto41	22.9	
Σ Freq. of Total	20.3	

15.5.5. Vienna

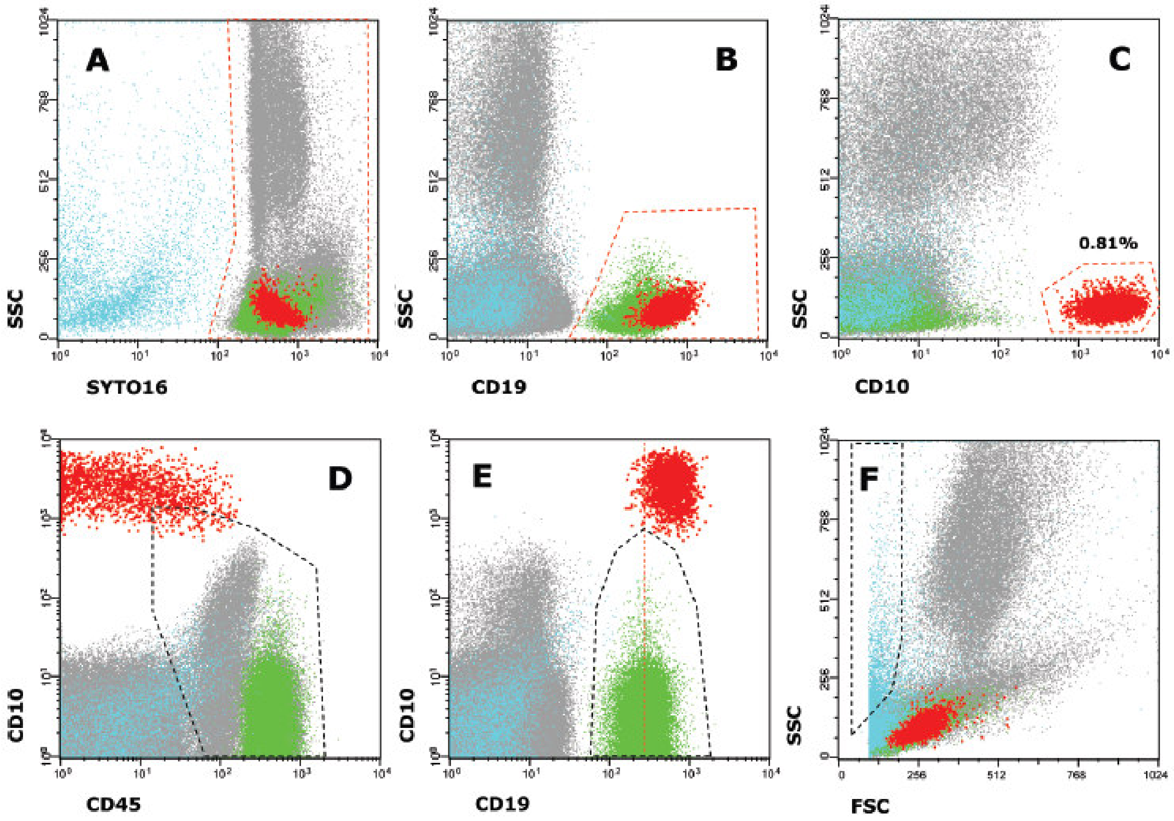

Fig. 1. Representative dot plots exemplifying the analysis and gating strategy in MRD assessment. This Day-15 BM sample from a patient with BCP-ALL was acquired on a BD FACSCalibur™ using PAINT-A-GATE™ software for analysis. In (**A**), gating on SYTO®16-positive, i.e., nucleated events is shown (irrelevant events in blue). B cells are then identified by plotting CD19 against SSC (**B**) and potential leukemic CD19^{+} cells (red) based on expression of the immaturity marker CD10 (**C**). Normal B cells (herein mature, CD10negCD45^{+++}) are painted green. Subsequently, in dual-color plots the supposedly leukemic cells are checked for leukemia-associated phenotypic aberrations in order to define MRD. Major aberrations, as CD45-negativity and CD10 hyper-expression (**D**), and minor leukemia-associated features (CD19 overexpression on immature B cells in **E**) can be found in this case. Dotted polygonal gates indicate the typical regions of normal B cells, including immature (D) or only mature cells (E), as obtained from cross-lineage background controls (e.g., BCP-panel stained in T-ALL follow-up samples from various time-points). Note that some MRD cells overlap with the normal region in (D), but these can undoubtedly be added to the leukemic cluster. Finally, the FSC/SSC plot (**F**) is used to inspect the morphological appearance of cells in order to exclude events from further calculations if appearing in the "dead cell" region (dotted polygon), which is specifically prone to autofluorescence.

15.6. Center-specific examples of cross-lineage controls

15.6.1. Vienna

B-cell background day15 BM – BCP-tube 1

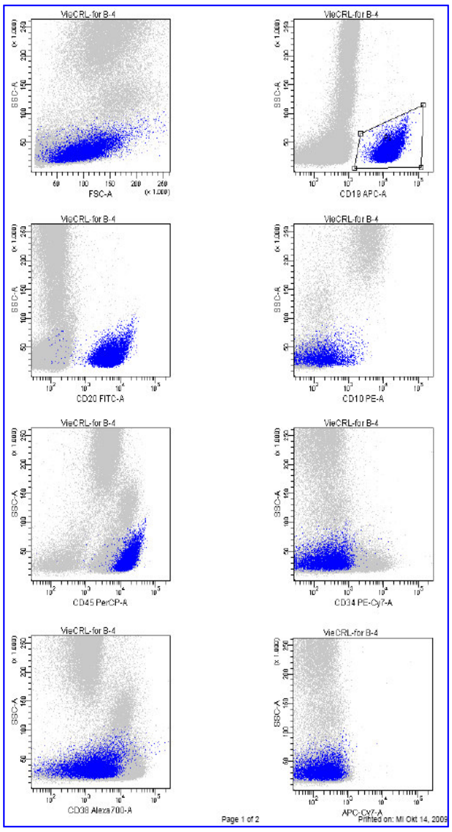

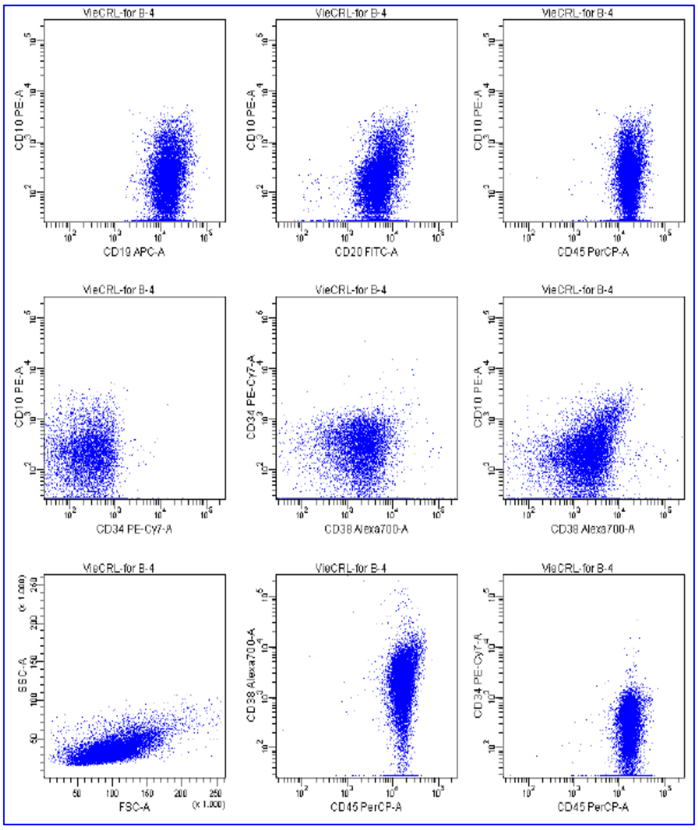

B-cell background day15 BM – BCP-tube 2

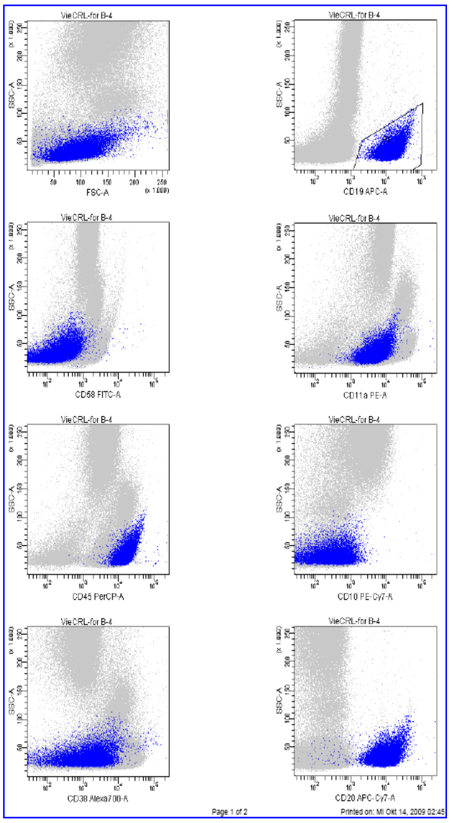

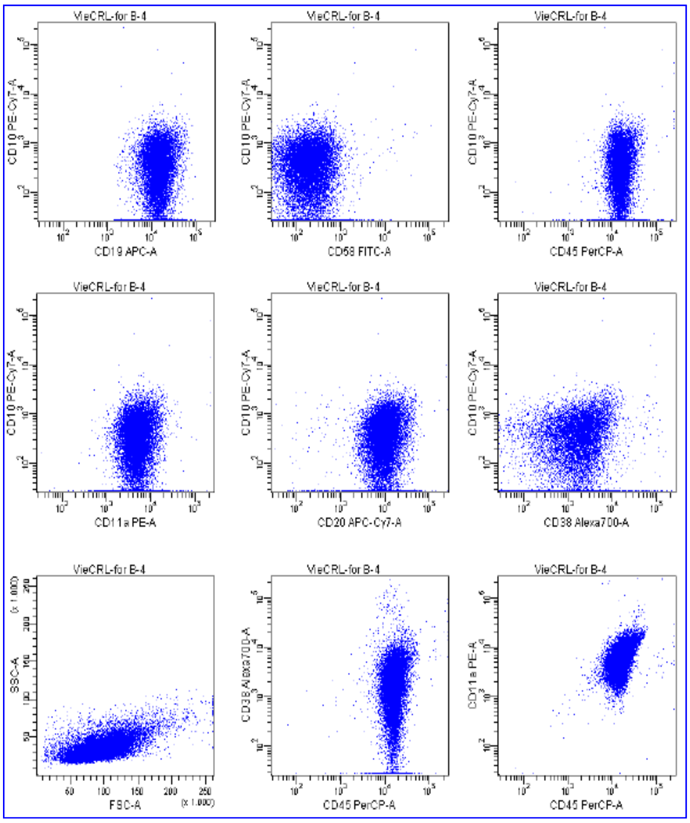

T-cell background day15 BM – T-tube 1

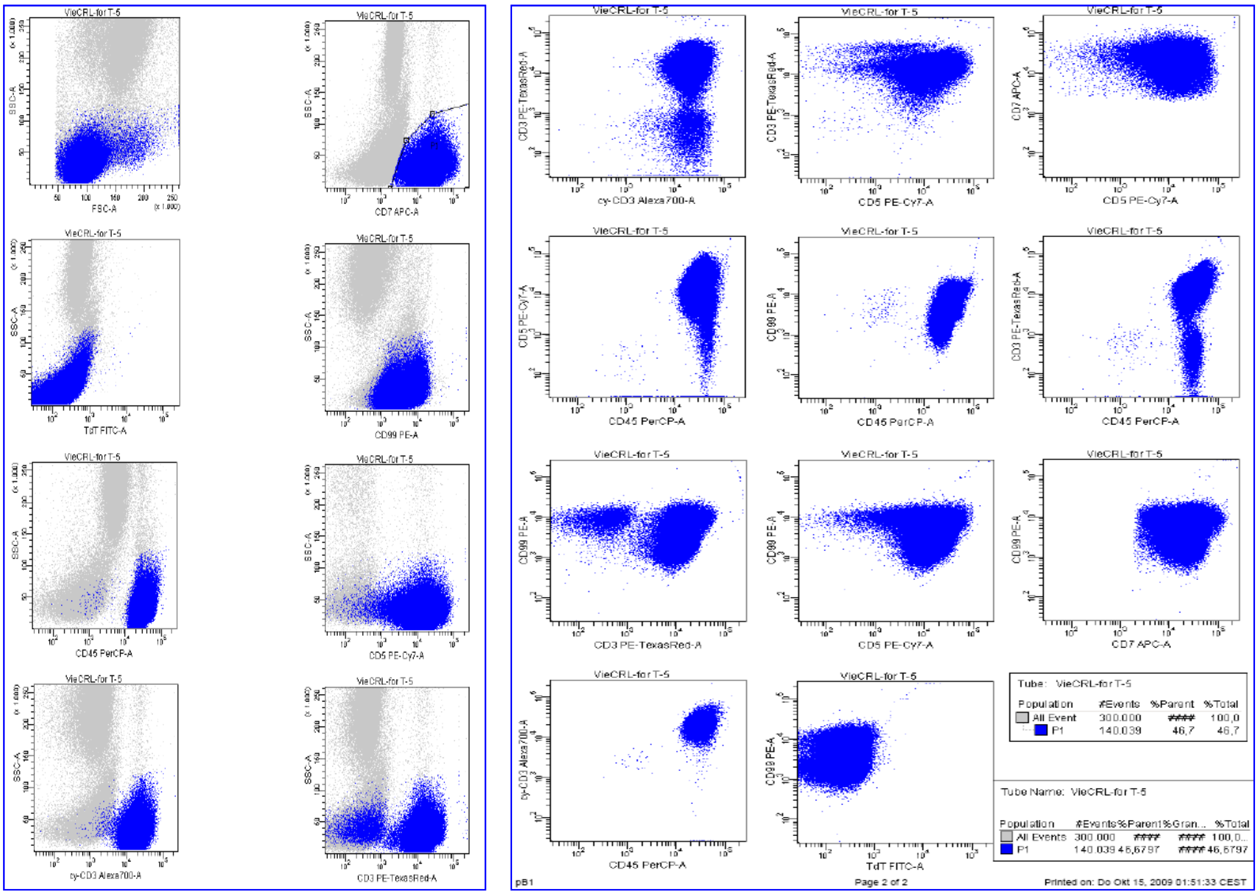

T-cell background day15 BM – T-tube 2

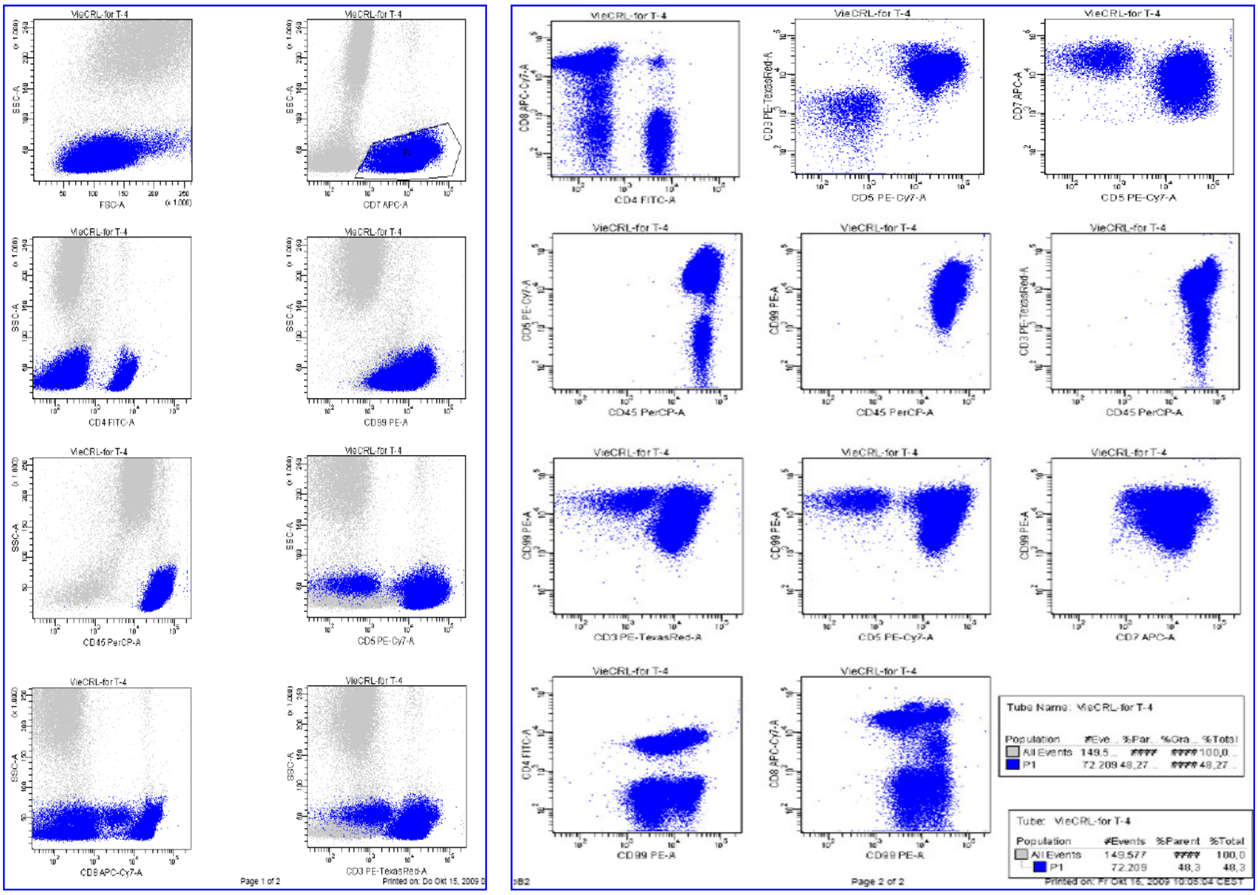

15.6.2. Petach-Tikva

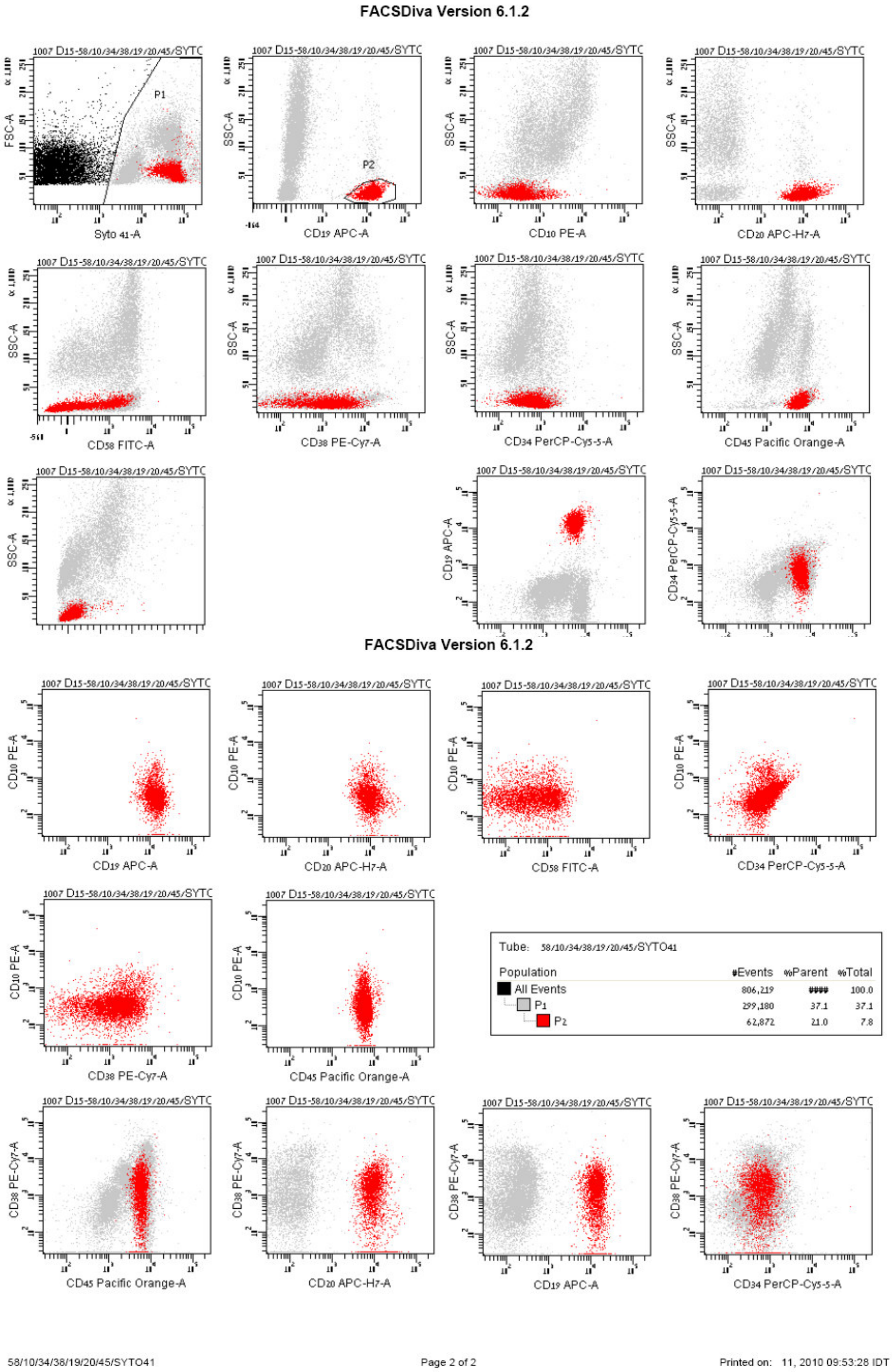

FACSDiva Version 6.1.2

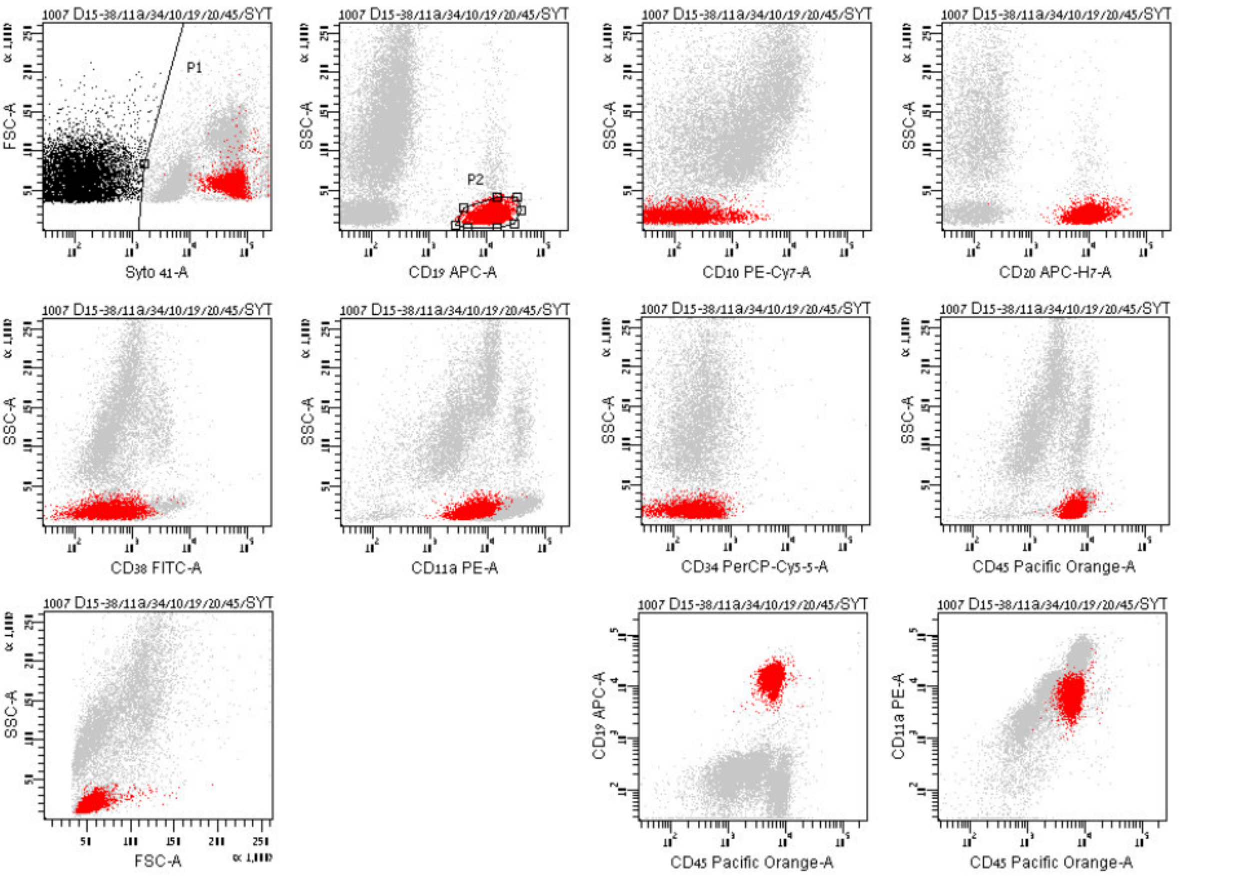

FACSDiva Version 6.1.2

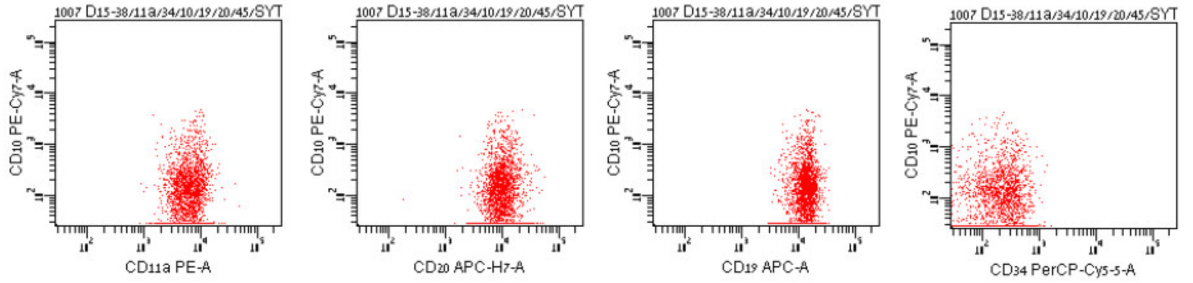

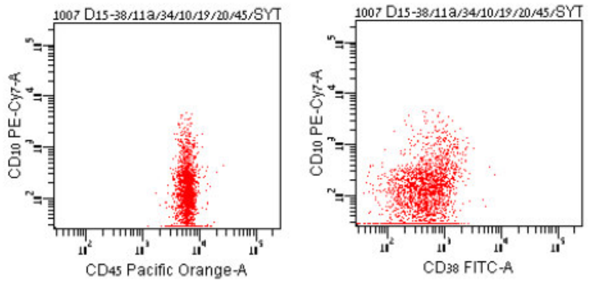

Tube: 38/11a/34/10/19/20/45/SYTO41

Population	#Events	%Parent	%Total
All Events	776,325	####	100.0
P1	304,487	39.2	39.2
P2	65,591	21.5	8.4

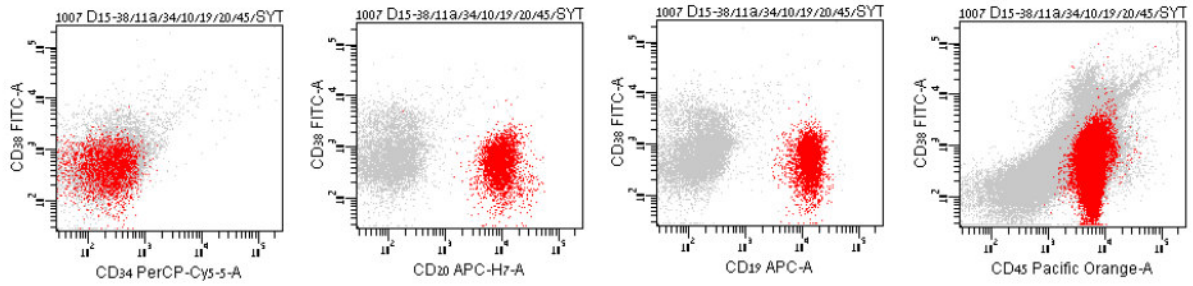

FACSDiva Version 6.1.2

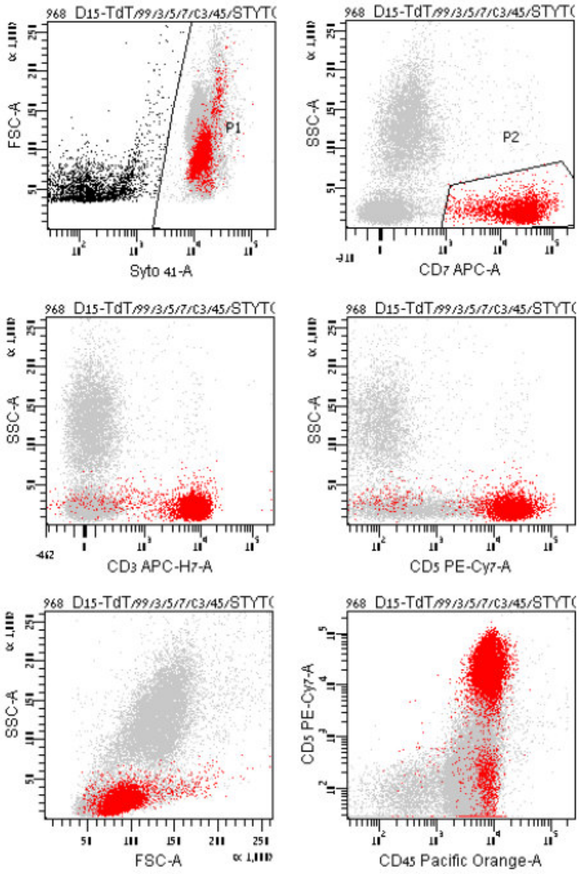

Tube: TdT/99/3/5/7/C3/45/STYTO41

Population	#Events	%Parent	%Total
All Events	300,000	####	100.0
P1	267,983	89.3	89.3
P2	64,532	24.1	21.5

FACSDiva Version 6.1.2

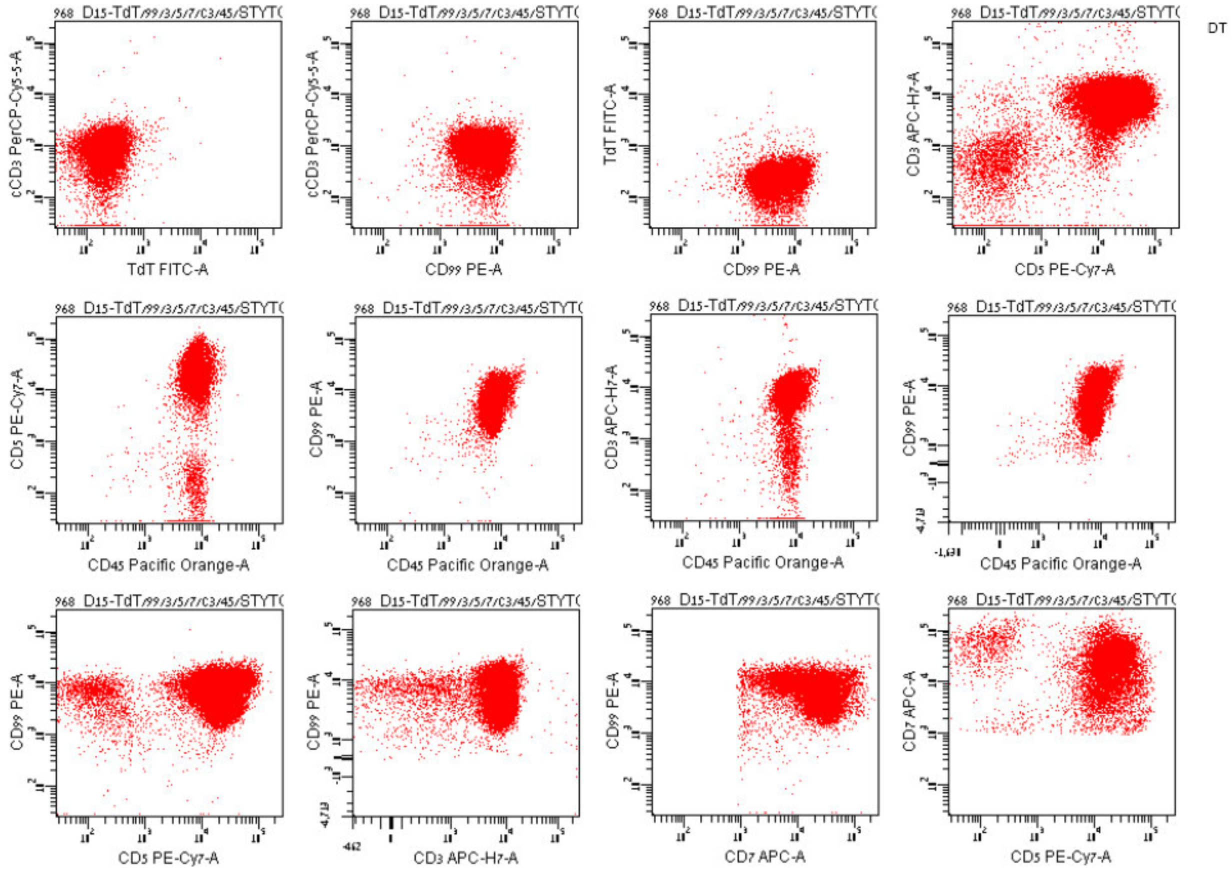

FACSDiva Version 6.1.2

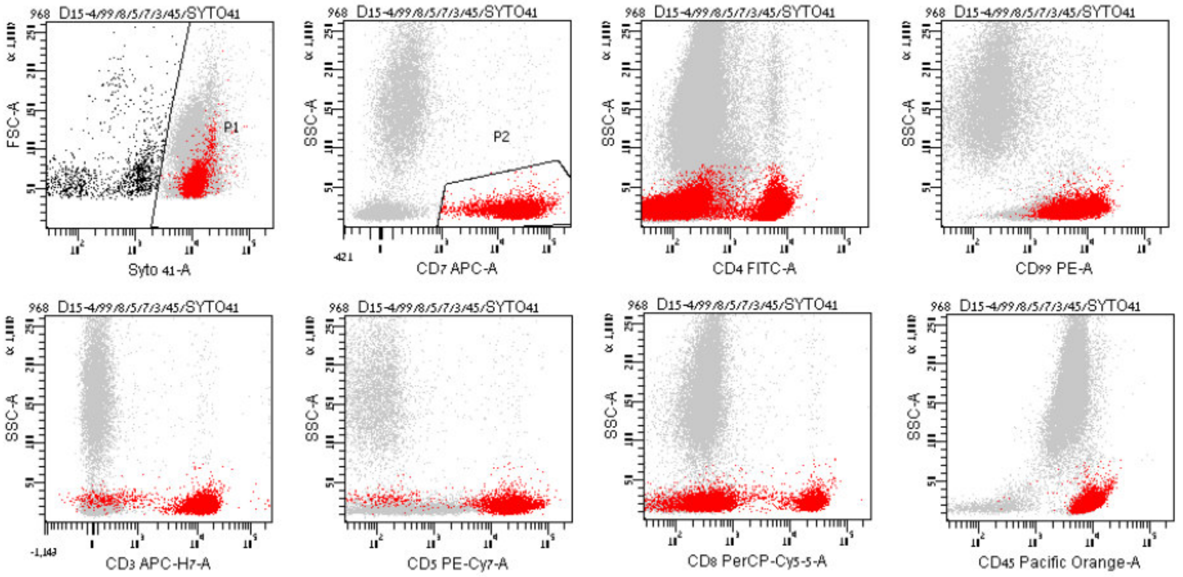

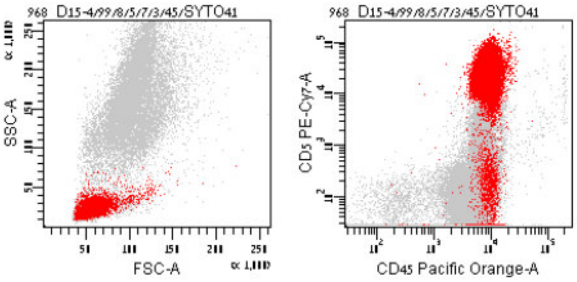

Tube: 4/99/8/5/7/3/45/SYTO41

Population	#Events	%Parent	%Total
All Events	352,893	####	100.0
P1	334,114	94.7	94.7
P2	88,323	26.4	25.0

FACSDiva Version 6.1.2

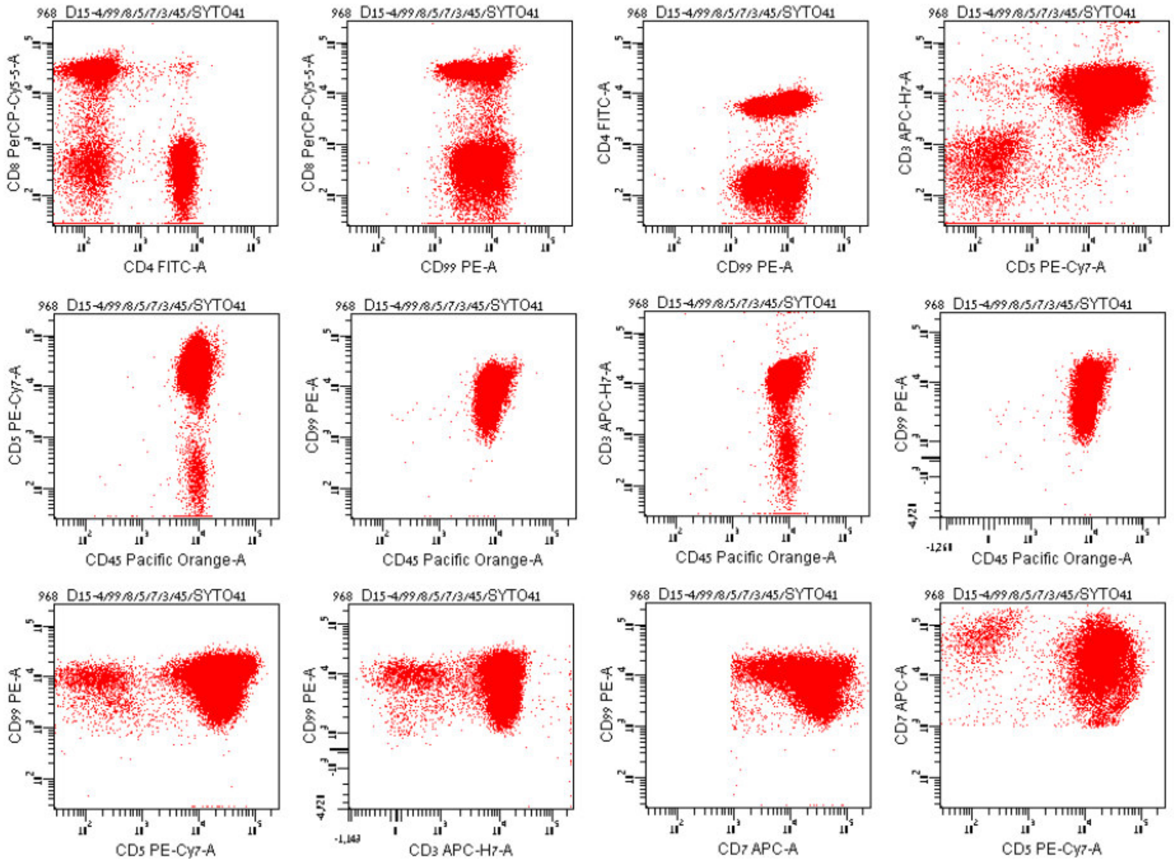

Supplement: Supplementary file 1 [file cancers-13-06148-s001.zip › Supplemental File 1 SOP AIEOP-BFM.pdf]
