# Supplementary material for: An Extensive Quality Control and Quality Assurance (QC/QA) Program Significantly Improves Inter-Laboratory Concordance Rates of Flow-Cytometric Minimal Residual Disease Assessment in Acute Lymphoblastic Leukemia: An I-BFM-FLOW-Network Report
Source: Cancers (Basel). 2021 Dec 6;13(23):6148. doi: 10.3390/cancers13236148 (PMC8656726; doi:10.3390/cancers13236148)
Supplement: Supplementary file 1 [file cancers-13-06148-s001.zip › Supplemental File 2 SOP ALL-IC 2009.pdf]

# ALL IC Protocol 2009

**Flow cytometric analysis of minimal residual disease (MRD) in pediatric ALL**

## 1. Monitoring and adjustment of settings on the flow cytometer

## Monitoring

**Aim:**
Performing MRD on a flow cytometer affords that multiple samples of an individual patient taken at several occasions in the follow-up period of weeks or months are to be studied. Due to treatment, expression of antigens may differ by biological causes between time-points and cell numbers in samples may change. To assure stability of results at least with respect to the methodological approach, monitoring of technical performance and device set up is of utmost importance. The following guidelines are intended to secure this issue and to make comparisons of data possible.

**Intralaboratory monitoring:**
This is a sequence of quality controls that should be performed on flow cytometers used for MRD assessment in each institution. Besides yearly check up through an accredited company, controls must be carried out every two weeks. In the following a proposal containing BD applications will be made, but this may be performed with corresponding tools on other flow cytometers than FACSCalibur.
The automated FACSComp program has to be run every two weeks. For running FACSComp, Calibrite 3 beads (BD#340486) extended with APC beads (BD#340487) are necessary. FACSComp controls device set up including time delay (for technical support and interpretation of data ask local BD support).

**Interlaboratory monitoring:**
MRD determination by flow leads to results which are examinable and must meet certain criteria. To achieve this aim, appropriate teaching and longitudinal quality control of involved staff must be assured. Standards concerning sample preparation and performance must be adhered to. For quality control, discussion of problematic cases as well as teaching a common platform is highly recommendable to warrant a rapid LMD file exchange. Special attention has to be put to new staff.
**In order to expect uniform data analysis it is required that ALL IC 2009 participants successfully complete 2 sendarounds of LMD file analysis.**
Ring test trials based on exchange of non-selected (spotted by time points) LMD files, or of patient samples and spiked specimens (mixture of leukemic cells from normal samples at diagnosis with normal PB or BM are of great value to determine the quality of performance when multiple laboratories co-operate. LMD file exchange is particularly useful to access the ability of staff in terms of post-acquisition skills, which is most crucial in MRD assessment because depending on the human factor in dot plot interpretation. Technically, evaluation of exchanged LMD files can be hampered by interlaboratory differences in the technical devices used for acquisition which may also disturb appropriate import of files into cross-company software for analysis.
Recently, cell stabilizing reagents (TransFix from Cytomark, distributed by Invitrogen) became available which makes sample exchange feasible even under consideration of several

days of time lapse between sampling and analysis due to transport over boarders. About the use of TransFix further investigations have to be performed.
The most promising alternative for external quality control monitoring will be the participation in the UK-NEQAS MRD analysis sendarounds.

## Adjustment of setting the flow cytometer (e.g. FACSCalibur)

**Aim:**
Standardized flow cytometer setup secures comparable results in participating institutions. Nevertheless, settings can not be transferred from one cytometer to the other, because each device has its own peculiarities. Certainly each institution has its own approach, but adherence to a backbone of common guidelines is recommended.

**Guidelines:**
General: Stain PB cells with mAbs in FITC, PE, PerCP, APC, and SYTO16 (per tube only one mAb) plus extra tube without staining. Make sure to have enough cells and choose mAbs giving bright signals (3$^{rd}$ log); prefer antigens positive on lymphocytes (e.g. CD3+). Importantly, the preparation of the samples must be used for MRD monitoring.

Setting:
1. put all compensation values to zero
2. show histograms for FL1 to FL4
3. show dot plots according to compensation possibilities
4. acquire unstained tube
5. adjust FSC and SSC (P1 and P2) linear values to optimize gates
6. gate on lymphocytes, exclude debris
7. adjust PMT (P3 to P7 (APC)) in log values to put auto-fluorescence of unstained cells into first log.
8. acquire FITC tube and adjust FL2-FL1 (second compensation)
9. acquire PE tube and adjust FL1-FL2 (first comp) and FL3-FL2 (fourth comp)
10. acquire PerCP tube and adjust FL4-FL3 (sixth compensation)
11. acquire APC tube and adjust FL3-FL4 (fifth compensation)
12. acquire SYTO16 tube (show all cells) and optimize FL2-FL1 (second compensation)
13. values for SYTO16 must be noted and used according to mAb composition in tubes

## Determination of mAbs used in MRD monitoring

**Aim: All participants should use same combinations of essential mAbs but may use different dyes.**
The combination of mAbs per tube is based on a backbone of two mAbs: in BCP-ALL CD19 (as primary gate), CD10 (as immature/blast cell marker); and in T-ALL CD7 (as primary gate, positive in T and NK cells), CD3 (mature T-cells). Backbone staining warrants that in each tube out of the series of three tubes the relevant cells can be addressed by similar phenotypic appearance, so that the additional markers can be evaluated as if all markers were combined in one tube. Notably, the expression of antigens frequently changes under treatment. Phenotypic patterns during follow up should therefore not simply be considered similar as at diagnosis. Instead, it is recommended to compare phenotypes rather with the immediately

preceding time point. In analyzing the data the primary gate should be set in CD19 versus SSC correlations in BCP-ALL samples, and in CD7 versus SSC in T-ALLs, hence, it is recommended to use similar mAb conjugates for these two antigens throughout all tubes.
Note: It is necessary to use the same clone for CD3 cytoplasmatic and surface staining in order to prevent re-staining of surface epitopes during cytoplasmic staining (after prior surface staining) because of differences in epitopes. It is NOT necessary to engage negative control antibodies because PB and BM are sufficiently heterogeneous cell compartments, allowing for in-sample control by normal cell types which are negative for the antigens under investigation on MRD cells.

## ALL IC 2009 will use the following time points for MRD analysis:

1. **de novo sample**
2. **day 15 sample**

Optionally: day33 and week12 (before MTX) are recommended for research purposes.

## ALL IC 2009 Suggested panels:

**for BCP-ALL MRD**

| | | | | |
|---|---|---|---|---|
| **Tube 1** | **SYTO 16** | **CD10-PE** | **CD45-PerCP** | **CD19-APC** |
| **Tube 2** | **CD20-FITC** | **CD10-PE** | **CD34-PerCP-Cy5.5** | **CD19-APC** |
| **Tube 3** | **CD58-FITC** | **CD10-PE** | **CD45-PerCP** | **CD19-APC** |
| **Tube 4** | **CD10/CD20-FITC** | **CD38-PE** | **CD45-PerCP** | **CD19-APC** |

**for T-ALL MRD**

| | | | | |
|---|---|---|---|---|
| **Tube 1** | **SYTO 16** | **CD7-PE** | **CD45-PerCP** | **CD3-APC** |
| **Tube 2** | **CD99-FITC** | **CD7-PE** | **CD5-PerCP-Cy5.5** | **CD3-APC** |
| **Tube 3** | **TdT-FITC** | **CD7-PE** | **CD3-PerCP** | **cyCD3-APC** |
| **Tube 4** | **CD4-FITC** | **CD8-PE** | **CD45-PerCP** | **CD3-APC** |

## SYTO

**Aim:**
SYTO16 is DNA>RNA dye which readily stains nucleated cells without a need for permeabilization. This dye is used to determine all nucleated cells in samples in order to exclude non-nucleated events like erythrocytes, platelets and debris. Weakly it also stains reticulocytes (hence exclude these by gating on SYTO16 clearly positive events). SYTO 16 may be used like a mAb (but not added to cocktails) or added to already lysed material.
SYTO16 is measured in FL1-channel although is not FITC, therefore it needs a bit different strategies in compensation (see chapter adjustment). Ideally SYTO positive cells should lie in the 3rd log (not higher), to prevent overspill into other channels and to clearly separate positive from negative events.

**Procedure:**
SYTO 16 is delivered frozen (Invitrogen, molecular probes #S7578). The thawed stock solution can be aliquoted and refrozen, e.g. at -20˚C. Working solutions of SYT0 16 should be prepared as 1:1000 dilution preferably with PBS/BSA2%/0.1%NaN3 and can be stored at 4˚C in dark vials. This dilution is stable for months. If SYTO 16 working solution is prepared using isotonic saline, it is recommended to prepare the solution fresh every week. Add 2 µl of the 1:1000 working solution to 200 µl of sample volume, e.g. immediately before acquisition. If necessary, increase the input by 0,5 µl steps to find the optimum. Respective optimum amount per 700 000 cells can be added to individual MRD tubes either along with mAbs (in this case double the amount) or after the final wash immediately prior to acquisition (1x amount). If location is too low more SYTO16 can be added without the need for extra incubation time. Notably, intensity might decrease with storage of the dilution over weeks, discard dilution when values arrive in the $2^{nd}$ log.

## Usage of mAbs – amounts for staining

**Aim:**
Warrant saturating staining for best resolution of blast versus normal cells.

**General procedure:**
The amount of mAb as indicated by manufacturer.
Note: mAb amounts as recommended on data sheets from companies are adapted for staining specimens with homogeneous cell populations at saturating conditions. In case, mAb amounts can be optimized by titration. If mAb amounts for usage are determined by titration, documentation of experimental evidence supporting the use of mAb at the relevant dilution must be available in the center. This documentation should include the type of antibody, the amount determined for usage per MRD tube, the amount recommended by the manufacturer, and a summary of the titration experiment (date, cell type, and results). If titration is used to determine the amount of mAb insert instead of the manufacturer recommendation, mind that titration is needed with each new lot of mAb – compare to previous lot.

**Titration procedure:**
For titration of ALL-associated mAbs like TdT, CD5, CD7, CD10, CD19, CD34 BM or PB derived from de novo leukemia samples with respective antigen positivity should be used.

Make sure that the tested mAb are positive and the sample contains over 90% blasts. Frozen samples (in liquid nitrogen) can also be used but lower fluorescence may occur. The viability should be ≥90%. In case a cell line is used mind the usually higher autoflourescence.
For titration of mAbs as CD3, CD20, CD45, CD58 and CD99 PB of a healthy donor can also be used, cytoplasmatic CD3 staining must be determined separately.
Note: It is necessary to use the same clone for CD3 cytoplasmatic and surface staining in order to prevent re-staining of surface epitopes during cytoplasmic staining (after prior surface staining) because of differences in epitopes.

Detailed description:
1. Stain approximately 1 million cells per test in a maximum of 100 μL volume.
2. Preferably use step-wise longitudinal dilution technique for staining.
3. Use a negative control (this could be cells without mAb or with matched irrelevant mouse mAbs conjugated with respective fluorochromes).
4. Use a positive control (stain with the amount of mAb indicated on bottle or data sheet.)
5. Dilute mAb with PBS or just use lower input. The lowest step should be at least the tenth part of the positive control (example: positive control 20 μL; lowest titration step 2 μL).
6. Incubate, lyse, measure according to the protocol.
7. Analyze results in a histogram and compare Geomean MFIs. Choose the optimal concentration of mAb by recording the lowest Ab concentration that still saturates binding. In case of variable positivity in non-homogeneous samples (relevant for broadly expressed antigens: CD11a, CD38, CD45, CD58, CD99), gate on distinct subpopulation with highest expression.
8. Use then the double amount of the first saturating concentration of mAb!
9. Test lot-to-lot changes using the optimal amount of mAb of the old lot as positive control, and compare the result with those from the new lot: 1x, 2x, 0.5x the amount of the old lot (example: optimum is 2 μL of old lot; check 2 μL, 4 μL and 1 μL of the new reagent); compare MFI results, make changes if necessary.
10. Save files or print outs of titrations and record expiry dates and lot numbers of mAbs.

## Work with mAb cocktails

**Aim:**
Working with mAb cocktails has several advantages towards economization of work and avoidance of errors: pipetting steps can be reduced and expedited, and mAb input is assured and standardized. On the other hand, since tandem fluorochrome conjugates as PE-CY7 tend to instability their inclusion into cocktails is NOT recommended. In addition, manufacturers do not evaluate performance of their mAbs sold and stored as single reagents for performance when stored in cocktails. Therefore, accreditation of these reagents for in-vitro-diagnostics is limited to storage as recommended by the vendor. Usage of cocktails depends upon individual decisions and are recommended as long as the cocktails are rapidly used and prepared fresh again weekly or provided that stability is monitored.
Tandem conjugates and SYTO dyes must NOT be included in cocktails.

Procedure:
10 μL of cocktail must contain all mAb in the optimal concentration plus PBS/BSA/NaN3
(Example: 2 μL FITC+2 μL PE+1 μL PerCP+1 μL APC+4 μL PBS/BSA/NaN3).
**Note:**

Use cocktail according to recommended input of each single antibody (as indicated by manufacturer). In case mAb amounts for staining are determined by titration, use respective volumes: e.g. 10 µL of cocktail should contain mAbs for up to 1-2 million cells to be stained in a final volume of 50-100 µL. For volumes bigger than 100 µL (in low cellularity material) input of cocktail must be adjusted. This is 20 µL of cocktail for up to 200 µL end volume, and 30 µL for up to 300 µL or more.
Check stability of cocktails (preferably at bi-weekly intervals) using BD CompBeads: for details of procedure see below (chapter on Settings and Compensation).
Save files or print outs of stability assessments and record dates of evaluation together with records of the date of cocktail preparation.

## Calculation of utilized volumes (cell input)

**Aim:**
For MRD detection down to the 0.01% level (with a required resolution of 30 MRD events minimum to refer a sample as positive), an optimum of 300 000 nucleated cells need to be acquired. Since preparation leads to cell loss it is recommended to stain at least 700 000 cells at all time points except at diagnosis. At diagnosis, stain 100 000 and acquire 30 000 cells. Cell loss depends on storage and age of materials and on cell content.
**Procedure:**
1. Determine nucleated cell (NC) count. This can be performed using an automated hemo-counter (note that this value includes also normoblasts!)
2. Calculate necessary volume by formula (see also example below):
Input in µL = intended number of input cells/counter cell count (cells per µL)
3. Round up as necessary
4. Record NC count

**Example:**
If cell count in the sample is 2100 /µL (= 2.1 G/L) of PB/BM; therefore input per staining tube is 333 µL of original sample. (Formula: 700 000:2100=333)

## Staining and lysing of MRD samples

**Aim:**
Staining for MRD assessment should be done with a stain-lyse (=red cell lysis) approach.
**Procedure surface staining**:
1. Add mAbs into tube (e.g. falcon BD# 352052)
2. Add SYTO16 in appropriate tube
3. Add BM (volume according to calculation; minimum is 50 µL- in case of very high cellularity dilute BM with PBS up to 50 µL volume)
4. Vortex, incubate at RT, in dark for 15 min.
5. Add 2 ml of BD lysing solution (BD# 349202 predilution 1:10 with demin. water) Vortex, leave for 10 to 15 min at RT in the dark
6. Centrifuge, discard supernatant
7. Top up with washing solution, e.g. 4ml cold PBS
8. Centrifuge at 400g for 10 minutes
9. Tip off/aspirate supernatant

10. Resuspend in 200 µL PBS
11. Alternatively SYTO staining can be carried out at this poin (see chapter SYTO)
12. Proceed to Flow cytometer

**Remarks:**

To steps 1 and 11:
SYTO16 can also be added early together with mAbs (step 1), so that step 12 can be cancelled.
To step 4:
Add 1 ml lysis buffer, vortex, add second ml of buffer, doing so will prevent overspill of fluid.
2 ml of lysing buffer are sufficient for lysing up to 400 µL of BM in cases of low cellularity with >> 400 µL of input, the whole volume should be split into several tubes (for example 3 tubes with 400 µL input each); add lysing solution per tube as indicated above, and merge tubes before measurement.
If after 10 minutes of incubation with lysing buffer a sample does not appear clear extend incubation time up to 15 minutes; in very reluctant cases (lots of red cells after step 6) one may continue at step 7 (instead of 1x PBS) with hypotonic lysis (add 1 ml of deionized water for 30 sec and then add 1 ml of double concentrated PBS).
We have no experience with $NH_4CL$ but according to literature data are available showing that $NH_4CL$ -lysis renders comparable results to BD lysis solution. Usage of pharmacy-grade $NH_4CL$ is therefore an acceptable option.

**Intracellular staining:**

1. Execute point 1 to 4 from surface staining protocol
2. Add reagent A from IntraStain (Dako), increase reagent A volume proportionally to sample volume (per 50-100 µL BM staining volume add 100 µL of reagent A; increase in increments of 100 µL as necessary up to a maximum of 300 µL of reagent A)
3. Incubate for 15 min at RT in the dark
4. Wash once in 4 mL cold PBS
5. Centrifuge (400g 10 minutes), discard supernatant, vortex to fully resuspend the cell pellet
6. Add reagent B (similar volumes as in point 2 according to volume of resuspended pellet) and add mAbs for intracellular staining (i.e. TdT, cy-CD3)
7. Vortex and incubate 20 minutes at RT in the dark
8. Wash with 4 mL cold PBS
9. Adjust volume to 200 µL
10. Add SYTO (see chapter SYTO)
11. Proceed to acquisition on flow cytometer.

Remarks:
See tips for surface staining, like splitting into more tubes. Hypotonic lysis may be also applied as step 8

## Acquisition of cells at diagnosis and in follow up for MRD

**Aim:** Adequate numbers of acquired events are a prerequisite for sufficient test sensitivity.
**Guidelines:**

A number of 30 cellular events (dots) of similar overall features is the minimum amount to judge a sample MRD-positive. Hence, to warrant that a sample can reliably be judged at the threshold of 0.01% it is necessary that acquired events contain as much as possible relevant events. The proportion of relevant cells among all events is assessed by the SYTO16 stain (also needed of the calculation of MRD% among NC=SYTO16+). Hence, to keep the number of non-relevant events low a certain standard in sample preparation is necessary (see before for optimized lysis procedure). At time points in follow up with a low cellularity of supplied samples the number of tube to be investigated should be kept as low as necessary in order to warrant enough sample per relevant tube. Notably, aplastic samples sometimes also tend to poor lysis efficacy, which should be concentrated by increasing the cell input into the most relevant tubes, or by acquiring a higher than standard number of events. Gated acquisition of CD19+ or CD7+ events, respectively is a further alternative to be done in the very rare problematic cases.

At diagnosis acquire 30 000 events only

**Acquisition in BCP-ALL follow up:**
First tube: SYTO16/CD10/CD45/CD19
    Acquire 10 000 events (SYTO-count among all events)
    Define gate on SYTO+ cells
    Acquire 300 000 events in SYTO gate
    Record the number of total acquired events in the first tube
Second tube: CD20/CD10/CD34/CD19
    Acquire the number of events recorded by tube 1
Third tube: CD58/CD10/CD45/CD19
    Acquire the number of events recorded by tube 1
Fourth tube: CD10-CD20/CD38/CD45/CD19

**Acquisition in T-ALL follow up:**
Follow the above listed steps adjusted to T-ALL panel.

Control:
Evaluate percentage of SYTO16+ cells in tube 1. Collect SYTO%/all events data in a spread sheet to monitor lysis performance over time. The percentage of SYTO+ cells must be higher than 50%. Record simultaneously how often it is possible to acquire 300 000 events in the SYTO gate of tube 1. If this is a frequent event cell input must be increased.

**Report data:**

| ALL-IC 2009 | | | | |
|---|---|---|---|---|
| **Patient data** | | | | |
| Name: | | | | |
| Sex: | Male: | | Female: | |
| Date of birth: | | | | |
| Sample: | day 0: | | day 15: | |
| Date of diagnosis: | | | | |
| Onset of treatment: | | | | |
| Onset of sample staining | | | | |
| Sampling time: | | | | |

**Results**

| | | | |
|---|---|---|---|
| Number of total acquired events: | Tube 1 | | |
| | Tube 2 | | |
| | Tube 3 | | |
| | Tube 4 | | |
| Number of acquired syto+ events: | | | |
| % syto+ events among total: | | | |
| Blast % in each tube: | Tube 1 | | |
| | Tube 2 | | |
| | Tube 3 | | |
| | Tube 4 | | |
| % of CD45 negative events (blasts and debris excluded): | | | |
| Date | | | Signature |
