# Supplementary material for: An Extensive Quality Control and Quality Assurance (QC/QA) Program Significantly Improves Inter-Laboratory Concordance Rates of Flow-Cytometric Minimal Residual Disease Assessment in Acute Lymphoblastic Leukemia: An I-BFM-FLOW-Network Report
Source: Cancers (Basel). 2021 Dec 6;13(23):6148. doi: 10.3390/cancers13236148 (PMC8656726; doi:10.3390/cancers13236148)
Supplement: Supplementary file 1 [file cancers-13-06148-s001.zip › Supplemental File 3 Amendment to SOP ALL-IC_2014.pdf]

Amendment to ALL IC-BFM 2009
Standard Operating Procedure
≥6-color FLOW-MRD detection in ALL

Version June 2014

M.N. Dworzak/J. Kappelmayer

Marker recommendation for multi-color MRD monitoring

Aim: All participants should use the same combinations of essential antigens/mAb-clones as they have been using in the original 4-color set-up according to the original ALL IC 2009 SOP (see trial protocol for details), however, different fluorochrome-conjugates of these mAb clones can be used as this depends on the flow cytometer facilities available. If done accordingly, training and maturation based on 4/5-color assay performance will be accepted as given also after methodological up-grading to ≥6-colors.

Procedure: The combination of mAbs per tube is based on a backbone of at least four mAbs: in **BCP-ALL** **CD19** (as primary gate), **CD10** (as immature/blast cell marker), **CD20** (for discrimination of mature B-Cells) and **CD45** (as pan leukocyte marker and quality control of BM composition); and in **T-ALL** **CD7** (as primary gate, positive in T and NK cells), **CD3** (mature T-cells), **CD5** (positive in T cells including immature, and negative in NK cells) and **CD45** (pan-leukocyte marker and quality control of BM composition).

Notably, the **expression of antigens frequently changes under treatment**. Phenotypic patterns on d15 should therefore not be considered similar to diagnosis.

In analyzing the data the **primary gate** should be set in **CD19** versus SSC correlations in BCP-ALL samples, and in **CD7** versus SSC in T-ALLs, hence, it is **recommended to use similar mAb conjugates** for these two antigens **throughout all tubes**.

Recommended tube combinations for day 15 analysis (BCP-ALL)

6-color (order of antigens/channels as per local habits):

Tube 1: **CD10/CD19/CD20/CD34/CD45/SYTO**

Tube 2: **CD10/CD19/CD20/CD38/CD45/CD58**

Tube 3: optional; may include one CDXX antibody* (variable antigen usually in PE) out of CD11a (PE only!!), CD66c, CD123, CRLF2, CLEC12A together with backbone CD10/CD19/CD20/CD45.

8-color (order of antigens/channels as per local habits):

Tube 1: **CD10/CD19/CD20/CD34/CD38/CD45/CD58/SYTO**

Tube 2: optional CD10/CD19/CD20/CD38/CD45/CD58/CDXX*/SYTO

Recommendations:

**CD58**	**FITC** preferred
**CD10**	**to be included in each tube**; **PE,** PE-Cy7, PE-TR
**CD45**	**to be included in each tube**; **PerCP** preferred, ECD
**CD19**	**to be included in each tube**; **APC** preferred, PC7
**CD20**	**to be included in each tube**; FITC, ECD, APC-Cy7, or Pacific Blue
**CD34**	PE or PE-Cy7 preferred, APC
CD38	important not at least in B-I or CD10low/neg cases; FITC, Ax700

Recommended tube combinations for day 15 analysis (T-ALL)

6-color (order of antigens/channels as per local habits):

Tube 1: **CD3/CD5/CD7/CD45/CD99/SYTO** (all)

Tube 2 (perm.): **cyCD3/sCD3/CD7/CD45/CD99/TdT** (in immature T-ALL)

Tube 3: **CD3/CD4/CD5/CD7/CD8/CD45** (in mature T-ALL)

Tube 4: optional; may include antibodies to TCR, CD16&CD56, CD34, CD117 (ETP!) together with backbone CD3/CD5/CD7/CD45.

8-color (order of antigens/channels as per local habits):

Tube 1: **CD3/CD4/CD5/CD7/CD8/CD45/CD99/SYTO** (all)

Tube 2 (perm.): **cyCD3/sCD3/CD5/CD7/CD45/CD99/TdT/SYTO** (all)

Tube 3: optional; may include antibodies to TCR, CD16&CD56, CD34, CD117 (ETP!) together with backbone CD3/CD5/CD7/CD45.

Recommendations:

**CD99**	FITC or PE
**sCD3**	**to be included in each tube**; PE-TR (=ECD), APC
**CD45**	**to be included in each tube**; **PerCP** preferred, ECD, APC-Cy7
**CD5**	**to be included in each tube**; **PE or PC7**
**CD7**	**to be included in each tube**; **APC** preferred, PE, PC5
**cyCD3**	PC7, Ax700
**CD4**	essential in T-IV (mature); FITC, Ax700
**CD8**	essential in T-IV (mature); PE, APC-Cy7

Note: It is necessary to **use the same clone for CD3 cytoplasmatic and surface staining** (or at least clones against the same epitope) in order to prevent re-staining of surface epitopes during cytoplasmic staining (after prior surface staining) because of differences in epitopes.

It is NOT necessary to engage negative control antibodies because PB and BM are sufficiently heterogeneous cell compartments, allowing for in-sample control by normal cell types which are negative for the antigens under investigation on MRD cells.

QC: Centers should **keep records of specifications** of their mAbs including Lot-No., date of first usage of individual mAb vial, date of emptying, expiry date.

As **templates of normal background** each center should **acquire and retain LMD files** of at least three day 15 BM samples stained in cross-lineage set-up with the center-specific panel for BCP- as well as T-ALL (e.g. 3 BCP-follow-up samples stained with the T-panel plus 3 T-follow-up samples stained with the BCP-panel).

SYTO

Aim: SYTO16 and SYTO41 are DNA>RNA dyes which readily stain nucleated cells without a need for permeabilization. These dyes are used to determine all nucleated cells in samples in order to exclude non-nucleated events like erythrocytes, platelets, and debris. SYTO16 is measured in FL1-channel whereas SYTO 41 needs a violet laser for excitation. Ideally SYTO positive cells should lie in the 3rd log (not higher), to prevent overspill into other channels and to clearly separate positive from negative events.

Procedure:
SYTO 16 is delivered frozen (Invitrogen, molecular probes #S7578). The thawed stock solution can be aliquoted and refrozen, e.g. at -20°. Working solutions of SYT0 16 should be prepared as 1:1000 dilution preferably with PBS/BSA2%/0.1%NaN3 and can be stored at 4° in dark vials. This dilution is stable for months. If SYTO 16 working solution is prepared using isotonic saline, it is recommended to prepare the solution fresh every week. Add 2 µl of the 1:1000 working solution to 200 µl of sample volume, e.g. immediately before acquisition. If not bright enough add 1 further µl.

SYTO 41 is delivered frozen (Invitrogen, molecular probes #S11352) and can be handled as above (keeping an intermediary stock of 1:200 may be useful), but for working solutions SYTO 41 should be diluted 1:2000 with 20mM TRIS buffer pH7.5 and stored at 4° in dark vials. Add approx. 1 µl per tube of sample before acquisition when prepared fresh. Increase amount by 1 µl for each additional day the dilution is used. Brightness diminishes significantly within one week – use fresh dilution.

Note: A vitality check (PI, 7AAD etc.) is NOT required because not unambiguously reflecting the in vivo situation at the time of BM/PB sampling – apoptosis may have been induced during transport and preparation. As long as scattering properties of cells are largely preserved, cells are included in MRD estimates (even if they would take up dead-cell dyes).

Analysis guidelines for MRD detection

Aim: Sensitive, specific and reproducible quantification of ALL-MRD. For FLOW-based stratification using the algorithm »**d15 BM MRD**: FLR is <0.1%, FHR is ≥10%, FMR all others« it is **necessary to be sensitive and specific at the 0.1% level**.

Procedure:

- Define primary plots (x-/y-axis): FSC/SSC, SYTO/SSCorFSC, all CDxx/SSC
- Define secondary plots (CDxx vs CDyy; recommended set-up given below; in parentheses=as available):
  - **BCP-ALL**:
    CD19 vs CD10
    CD58 vs CD10
    CD45 vs CD10
    CD34 vs CD10
    CD20 vs CD10
    CD38 vs CD10
    (CD11a vs CD10)
    (CD45 vs CD11a)
    (CD45 vs CD38)
  - Optional in **CD10$^{low/neg}$ BCP-ALL**
    CD19 vs CD38
    CD34 vs CD38
    CD10 vs CD38
    CD20 vs CD38
    CD45 vs CD38
  - **T-ALL**:
    sCD3 vs cyCD3
    sCD3 vs CD5
    CD7 vs CD5
    CD45 vs CD5
    CD45 vs CD99
    CD45 vs sCD3
    CD45 vs cyCD3
    CD7 vs CD99
    CD5 vs CD99
    sCD3 vs CD99
    (TdT vs CD99)
    CD4 vs CD8
- If not done already at acquisition, **gate SYTO+ events** in SYTO/SSCorFSC plot (region1) and omit SYTO^{neg} events

Note: include SYTO41^{low}FSC^{high} cells (which may appear after BD lysis) into SYTO+ compartment (SYTO/FSC plot)

- **Gate CD19+ or CD7+ cells** with **SSC$^{low\ to\ intermediate}$** (region 2)
- In all **secondary plots display in particular region 2 cells**
  Note: occasionally, the CD19+ gate does not contain all CD10+SSC$^{low/int}$ cells due to very low CD19 expression! In case, adjust region 2 to include all CD10+ cells by extending region 2 into the CD19^{neg}SSC$^{low/int}$ area!
- **Gate potential MRD containing compartment among region 2** (= region 3):
  - In CD10$^{high/intermediate}$ BCP-ALL: gate **CD10+** cells
  - In CD10$^{low/negative}$ BCP-ALL: gate **CD20^{neg}** cells
  - In sCD3$^{low/negative}$ T-ALL: gate **sCD3$^{low/neg}$** cells
  - In sCD3^{high} T-ALL: gate **sCD3+** cells
- **Investigate secondary plots for leukemia-associated aberrations** and **insert sub-gate to MRD-population** if present (region 4 – MRD gate): at least one of the patterns given below will distinguish MRD cells from normal residual lymphocytes in BM at day15. »**Cluster gating«**, that is gating of cells which form a distinctive cluster based on their antigen combination patterns, **is mandatory**.
  - **CD10$^{high/intermediate}$ BCP-ALL**: on **CD10+CD19+**(/-) cells (region 3; these cells will contain mostly leukemic blasts, and possibly some mature B-cells with low CD10 expression=CD20^{high}CD45^{high})
    - confine interest to the **CD10$^{++/+++}$** cells if existing
    - check for **CD19 over-/underexpression**
    - check for **CD20 under**-/**overexpression**
    - check for **CD45 underexpression**
    - check for **CD58 overexpression**
    - check for **CD34 (over)expression**
    - check for **CD11a under**-/(over)**expression**
    - check for **CD38 underexpression**
  - **CD10$^{low/negative}$ BCP-ALL**: on **CD20^{neg}CD19+** cells (region 3; these cells will contain leukemic blasts, and possibly plasma cells=CD38^{+++})
    - confine interest to the **CD38$^{+/++}$** population if existing
    - check for **CD10 underexpression**
    - check for **CD45 underexpression**
    - check for **CD34 (over)expression**
    - check for **CD38 underexpression**
    - check for **CD58 overexpression**
    - check for **CD11a under**-/(over)**expression**, if available

- **sCD3$^{low/negative}$ T-ALL**: on **sCD3**$^{low/neg}$**CD7+** cells (region 3; these cells will contain leukemic blasts, and normal NK cells=CD5^{neg})
  - confine interest to **cyCD3^{pos} and/or CD5^{pos}** cells if existing
  - check for **CD99 overexpression**
  - check for **TdT expression**
  - check for **CD45 underexpression**
  - check for **CD7 overexpression**
  - check for **CD5 low expression**

  Note: few cells cyCD3+sCD3$^{low/neg}$ can be seen in most BM samples (≤0.1%). These cells are also NK cells and do not express CD5 as opposed to T-ALL blasts!

  Note: cells CD99^{high}TdT^{pos}CD7^{low}sCD3^{neg} may be seen in (regenerating) BM samples. If cyCD3^{neg} these cells will rather be immature B cell precursors and should not simply be misinterpreted as T-ALL blasts! Due to residual hematopoietic function in some cases of T-ALL with incomplete marrow infiltration at diagnosis, such immature B cell precursors may be encountered even at day 15.

- **sCD3^{high} T-ALL**: on **sCD3$^{+/++}$CD7+** cells (region 3; these cells will contain leukemic blasts, and normal T cells=CD45^{++})
  - check for **sCD3 inhomogeneous expression**
  - confine interest to subfractions of sCD3$^{+/++}$population and
  - check for **CD99 overexpression**
  - check for **TdT expression**
  - check for **CD45 underexpression**
  - check for **CD7 under-/overexpression**
  - check for following aberrations **only in combination** with previous:
    - **CD5 low expression**
    - **CD4/CD8 double-positivity**
    - **CD4/CD8 double-negativity**
    - **CD4 weak expression**
    - **CD8 weak expression**

- In plot FSC/SSC (displaying region 2 only) set gate (region 5) on lymphoid cells to exclude distinct FSC$^{very\ low}$/SSC^{intermediate} events (**debris area prone to autofluorescence**) as necessary.

- In plot CD45/SSC (displaying region 1 cells) set gate (region 6) on $CD45^{neg}$ cells. This region 6 will contain normoblasts, as well as occasionally leukemic cells. To contain only **erythroblasts for quantification**, make sure that region 2 cells are then excluded from region 6.
- Make sure that population statistics is based on SYTO+ = 100%
- Display gating hierarchy and region statistics
- Save, export and print (as necessary)
- Report data of each individual tube to documentation file:
  - **Number of total acquired SYTO+ events** (report as primary value if SYTO+ acquisition is performed; can be calculated by using the following two parameters also)
  - **Number of total acquired events** (report if SYTO+ acquisition is NOT performed)
  - **% SYTO+ events among total events** (report if SYTO+ acquisition is NOT performed)
  - **MRD population in % of SYTO+** (region 4 or region 5, see above)
  - **Absolute number of MRD cells in the acquisition**
  - **MRD vote positive or negative**

    Mind that a MRD-cluster needs to contain:

    ≥30 cells with related characteristics for **positivity as per ALL IC 2009**
  - **Erythroblast population in % of SYTO+** (region 6 excluding leukemic blasts!).
- If more than one staining/tube is used, build final MRD-estimate of the sample
  - by that tube which shows the **clearest separation of the MRD cluster and usually the highest MRD-value** (**it is not intended to build average values**).
  - in **T-ALL**, the value of a **surface-only tube** should be used for MRD-quantification wherever possible, whereas the permeabilized stain should be used for validation only (permeabilization may lead to skewing of BM composition).

Reporting for clinical application

Aim: Warrant timely reporting of day15 MRD results **to the clinical study office or to clinical site as per national practice**.

Procedure: Report as quick as possible in order to allow clinical use of day15-MRD-results by day 22 (i.e. Mind a possible reduction of therapy for Low risk patients!!): this means reporting **at least within one week after sampling.**

Reporting for clinical application is legitimate only in the following situations:

- **reporting lab has acquired iBFM-FLOW-network maturation** (per ALL-lineage)
- **reporting lab has received approval by the iBFM twinning partner for the respective case which needs clinical reporting** (use appended result sheet for inter-laboratory communication)

The MRD data report should include items for unambiguous patient/sample identification and validation, as well as the Flow-MRD data including quality items. The **minimal required data set for the report includes**:

1. **Patient's name (first and family name) and/or code (study code, UPN)**
2. **Date of birth**
3. **Treatment protocol (e.g. ALL IC-BFM 2009)**
4. **Type of sample (e.g. bone marrow)**
5. **Time-point of assessment (e.g. day 15)**
6. **Date of sampling**
7. **Date of processing**
8. **Date of report**
9. **Signature of laboratory responsible**

10. **Sample votes** (according to the guidelines of this SOP):
    - »**MRD-Positive**«, or
    - »**MRD-Negative**«, or
    - »**Inadequate sample**«, in case, give explanation why:
        - Wrong sample (PB instead of BM, discrepancies of label to allotment)
        - **Too few cells for analysis** (<30 000 SYTO+ events per tube: i.e. FLR interpretation cannot be made)
        - Too poor quality to be analyzed, which may be due to
            - **very significant hemodilution**

              i.e. Erythroblasts (SYTO+CD45^{neg}lymphLIN^{neg}) <2% of NC
            - **protracted time from collection to processing**

              Note: <48 hours = favorable, <96 hours = usually acceptable
            - **loss of regular scattering properties** (e.g. massive cell death)

11. MRD-% among all nucleated cells (only if judged MRD-positive)

12. Interpretation of FLOW-based risk (only if NOT inadequate sample):

- »**FHR**« **(Flow high risk)**, i.e. MRD BM day15 ≥10% (of NC=SYTO+)
- »**FMR**« **(Flow medium risk)**, i.e. MRD BM day15 <10%, but ≥0.1% NC
- »**FLR**« **(Flow low risk)**, i.e. MRD BM day15 <0.1% NC
    - Note: in cases found potentially inadequate in terms of erythroblast count (e.g. <2%), blast persistence ≥10% allows giving FHR interpretation, but all other hemodiluted cases (having <10% blasts) should not be given a risk vote (because of hemodilution): rate as »**inadequate**«.

ALL IC-BFM FLOW-MRD result sheet

v2014

Center:

Patient data

Name or Code:			
Sample:	day 15:		day 33:
Date of diagnosis:			
Onset of treatment:			
Sampling date:			
Acquisition date:			

Results in detail

Number of total acquired events:			N= color stain:	
	Tube 1			
	Tube 2			
	Tube 3			
	Tube 4			
Number of acquired syto+ events:				
% syto+ events among total:				
Blast % among NC (recalculated):			MRD-vote (p/n)	
	Tube 1			
	Tube 2			
	Tube 3			
	Tube 4			
% of CD45 negative events, blasts/debris excluded:				

Total sample result (local center):

Quality	MRD p/n	MRD %	Risk

iBFM partner approval: **yes/no**

comments:

date: **name:**
