# Supplementary material for: An Extensive Quality Control and Quality Assurance (QC/QA) Program Significantly Improves Inter-Laboratory Concordance Rates of Flow-Cytometric Minimal Residual Disease Assessment in Acute Lymphoblastic Leukemia: An I-BFM-FLOW-Network Report
Source: Cancers (Basel). 2021 Dec 6;13(23):6148. doi: 10.3390/cancers13236148 (PMC8656726; doi:10.3390/cancers13236148)
Supplement: Supplementary file 1 [file cancers-13-06148-s001.zip › Supplemental File 4 iBFM FLOW Twinning Maturation.pdf]

iBFM FLOW *twinning* program

**ALL IC-BFM center:**	
**AIEOP-BFM partner:**	
**Start of co-operation:**	
**Training periods:**	

Track records ALL-MRD day 15

Guidelines

- "Maturation" to release MRD day15 results for clinical application without further control by the partner lab is granted to a center according to defined criteria.
- "Maturation" to release MRD day15 results for clinical application is separate by lineage.
- "Maturation" shall be granted upon completion of a series of 25 different patient sample pairs (dx and day15 MRD) per lineage jointly assessed and provided that NO GROSS failures to identify and quantify day 15 MRD at first-sight were recorded in the most recent half (i.e. 12 patients) of the series.
- Gross failures are defined as "recurrent (>2x) inadequacy of staining and analysis", "blast population missed", "gross *miss*-gating".
- Gross *miss*-gating is e.g. defined by ">= 5-fold discrepancy between trainee's and supervisor's MRD result".
- Simple differences at prognostic thresholds (e.g. 0.09% vs. 0.11%) are NOT regarded gross failures although leading to different stratification.
- Each gross failure necessitates further 3 day15 assessments (same lineage) without failure in order to grant "maturation".
- In case of uncertainty, partner labs will be open for consultation also after granting "maturation".
- Traceability (i.e n of cases, dates of analysis, n of failures…etc) of "maturation" has to be reported in an official track record form provided by the iBFM coordinator. The track record form is kept and completed at the AIEOP-BFM site based in parts on information provided by the ALL-IC center.
- "Maturation" accreditation must be communicated to and approved by the iBFM co-ordinator upon consensual suggestion by the twinning partners. Once consensus was found regarding "maturation", twinning partners should send the completed track record form to the iBFM FLOW co-ordinator. This can be accomplished at different time-points per lineage.
- The iBFM FLOW co-ordinator informs the ALL IC-BFM FLOW co-ordinator of granting "maturation" to an ALL IC-BFM FLOW center.

iBFM FLOW *twinning* program

**ALL IC-BFM center:**	
**AIEOP-BFM partner:**	
**Start of co-operation:**	
**Training periods:**	

Track record ALL-MRD day 15

B-lineage

([1]=ALL-IC center, [2]=AIEOP-BFM partner)

	patient code[1]	sampling date[1]	date of review[2]	failure[2] Y/N	comment (failure type)
**1**	SASOMO	12Nov2010	16Nov2010	N	/
**2**					
**3**					
**4**					
**5**					
**6**					
**7**					
**8**					
**9**					
**10**					
**11**					
**12**					
**13**					
**14**					
**15**					
**16**					
**17**					
**18**					
**19**					
**20**					
**21**					
**22**					
**23**					
**24**					
**25**					
26					
27					
28					

failure types: 1) >2 samples with poor quality staining, 2) blast population missed, 3) gross miss-gating (e.g. ≥5-fold discrepant MRD value from reviewer)

iBFM FLOW *twinning* program

**ALL IC-BFM center:**	
**AIEOP-BFM partner:**	
**Start of co-operation:**	
**Training periods:**	

Track record ALL-MRD day 15

B-lineage, continued

([1]=ALL-IC center, [2]=AIEOP-BFM partner)

	patient code[1]	sampling date[1]	date of review[2]	failure[2] Y/N	comment (failure type)
29					

failure types: 1) >2 samples with poor quality staining, 2) blast population missed, 3) gross miss-gating (e.g. ≥5-fold discrepant MRD value from reviewer)

iBFM FLOW *twinning* program

ALL IC-BFM center:	
AIEOP-BFM partner:	
Start of co-operation:	
Training periods:	

Track record ALL-MRD day 15

T-lineage

([1]=ALL-IC center, [2]=AIEOP-BFM partner)

	patient code[1]	sampling date[1]	date of review[2]	failure[2] Y/N	comment (failure type)
1					
2					
3					
4					
5					
6					
7					
8					
9					
10					
11					
12					
13					
14					
15					
16					
17					
18					
19					
20					
21					
22					
23					
24					
25					
26					
27					
28					

failure types: 1) >2 samples with poor quality staining, 2) blast population missed, 3) gross miss-gating (e.g. ≥5-fold discrepant MRD value from reviewer)

iBFM FLOW *twinning* program

**ALL IC-BFM center:**	
**AIEOP-BFM partner:**	
**Start of co-operation:**	
**Training periods:**	

Track record ALL-MRD day 15

T-lineage, continued

([1]=ALL-IC center, [2]=AIEOP-BFM partner)

	patient code[1]	sampling date[1]	date of review[2]	failure[2] Y/N	comment (failure type)
29					

failure types: 1) >2 samples with poor quality staining, 2) blast population missed,
3) gross miss-gating (e.g. ≥5-fold discrepant MRD value from reviewer)

iBFM FLOW *twinning* program

ALL IC-BFM center:	
AIEOP-BFM partner:	
Start of co-operation:	
Training periods:	
Maturation criteria fulfilled for B-Lin. ALL	YES/NO* Date:*
Maturation criteria fulfilled for T-Lin. ALL	YES/NO* Date:*

* to be filled by AIEOP-BFM partner

"Maturation" granted for B-lineage ALL

AIEOP-BFM supervisor*: ______________________________date:_____________________

iBFM FLOW network coordinator: __________________________date:_____________________

"Maturation" granted for T-lineage ALL

AIEOP-BFM supervisor*: ______________________________date:_____________________

iBFM FLOW network coordinator: __________________________date:_____________________

*to be signed by representative of the AIEOP-BFM partner center
