# Supplementary material for: An Extensive Quality Control and Quality Assurance (QC/QA) Program Significantly Improves Inter-Laboratory Concordance Rates of Flow-Cytometric Minimal Residual Disease Assessment in Acute Lymphoblastic Leukemia: An I-BFM-FLOW-Network Report
Source: Cancers (Basel). 2021 Dec 6;13(23):6148. doi: 10.3390/cancers13236148 (PMC8656726; doi:10.3390/cancers13236148)
Supplement: Supplementary file 1 [file cancers-13-06148-s001.zip › Supplemental File 5 Maturation Certificate.pdf]

# iBFM FLOW *twinning* program

| | |
|---|---|
| ALL IC-BFM center: | xxxx |
| AIEOP-BFM partner: | AUSTRIA Vienna |
| Start of co-operation: | 2002; renewed 15/10/2010 |
| Training periods: | 01/07/2002-31/07/2002 |
| Ring trials: | Successfully passed 2016/17/18 |
| Advanced maturation criteria fulfilled for B-ALL | YES Date: 23.05.2018 |

* to be filled by AIEOP-BFM partner

## "Maturation" granted for B-lineage ALL at regenerative time-points (day 78; after HR- and relapse cycles)

**AIEOP-BFM supervisor*:** Angela Schumich/Michael N. Dworzak date: 23.05.2018

**iBFM FLOW network coordinator:** Michael N. Dworzak date: 23.05.2018
