# Supplementary material for: An Extensive Quality Control and Quality Assurance (QC/QA) Program Significantly Improves Inter-Laboratory Concordance Rates of Flow-Cytometric Minimal Residual Disease Assessment in Acute Lymphoblastic Leukemia: An I-BFM-FLOW-Network Report
Source: Cancers (Basel). 2021 Dec 6;13(23):6148. doi: 10.3390/cancers13236148 (PMC8656726; doi:10.3390/cancers13236148)
Supplement: Supplementary file 1 [file cancers-13-06148-s001.zip › Supplemental File 7 Educational Slides.pdf]

Ring trials – LMD file send-arounds

Analysis of problematic cases

Educational Slides

BCP - REGENERATION LMD MRD trial 2020 (RT9)

Analysis of problematic cases

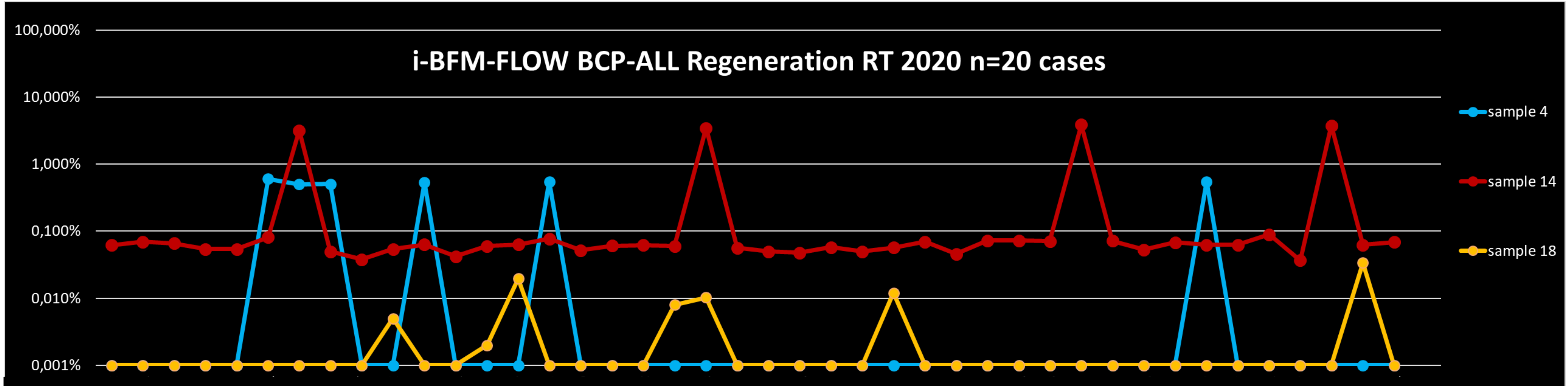

16 of all 28 failures came from 3 cases among 20; 12 other failures from 8 different cases
(820 samples analyzed in total; failure rate 28/820, i.e. 3.4%)

sample 4: PCR negative **sample 14: PCR 4x10-4** **sample 18: PCR negative**

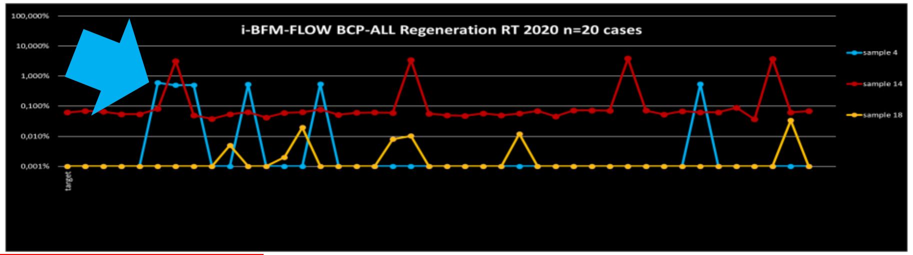

Case 4

RQ-PCR neg

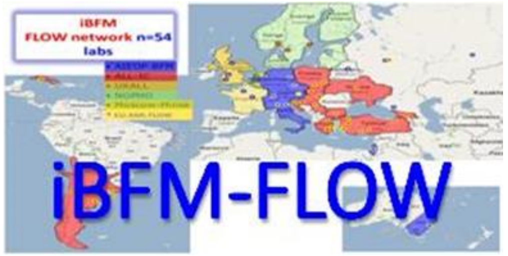

Target vote: negative

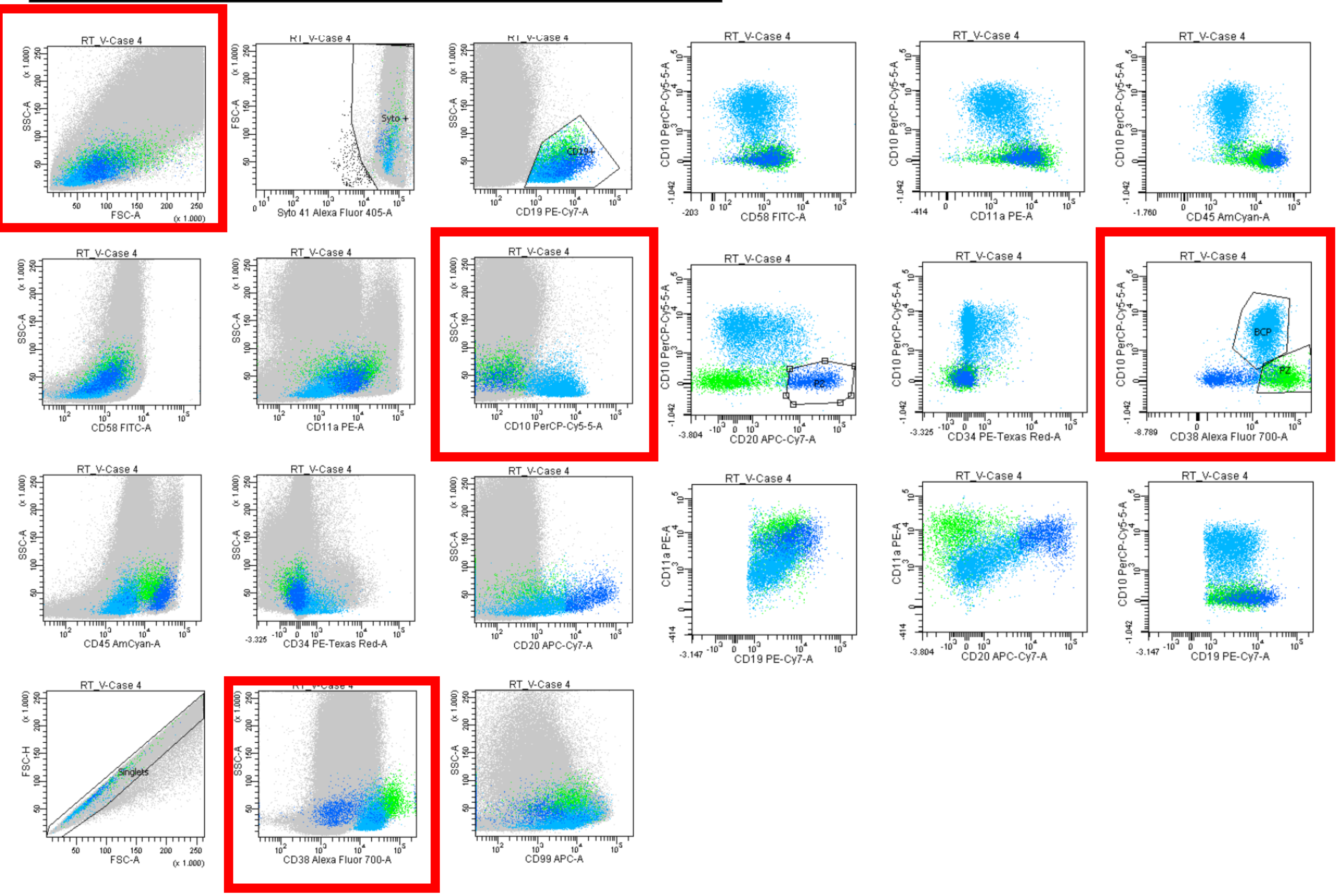

Problem:

Strong early BCP 1 & 2-regeneration; few mature BCP3

CD34 dim/negative

Solution:

mind very compact patterns e.g. in CD38, CD45, FSC

No aberration in any marker (except CD34)

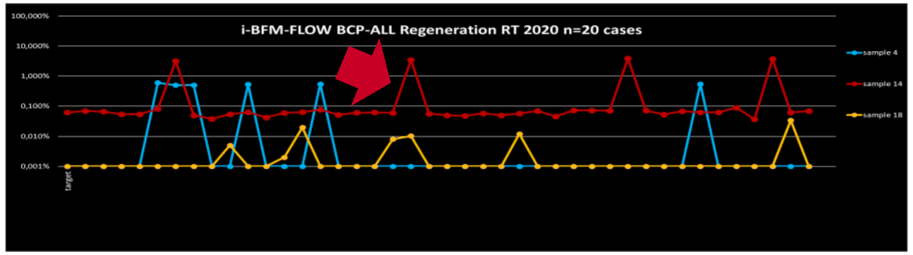

Case 14

RQ-PCR 4x10^{-4}

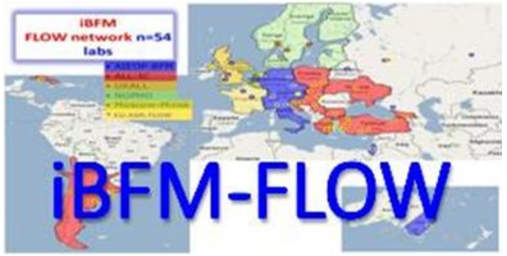

Target vote: positive 0.062%

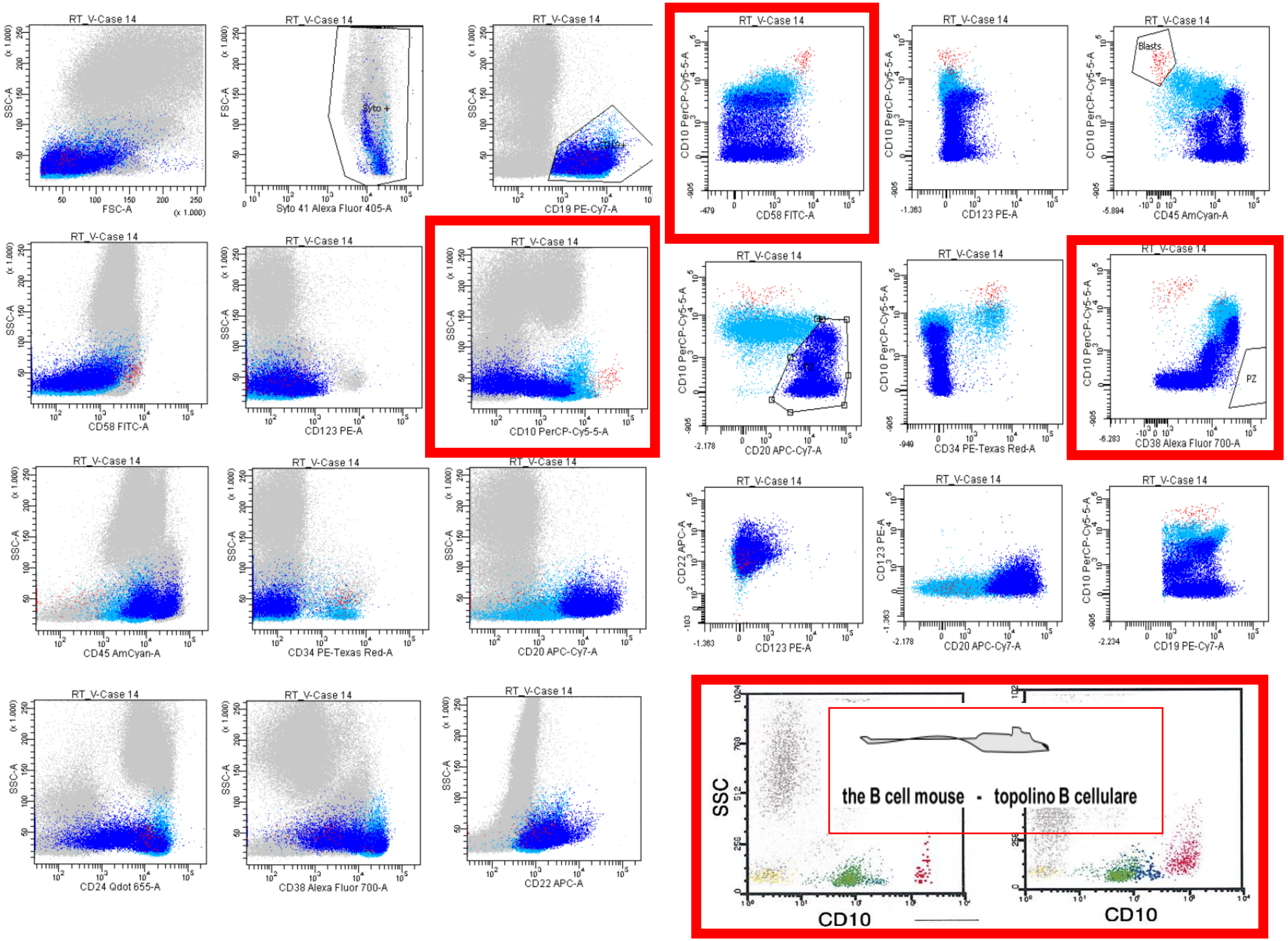

Problem:

Heavy regeneration

Coexistence of blasts & BCP
Mature BCP „echo“ population

Solutions: MANY
CD10 overexpression
SSC elevated
CD19 higher than BCP 1&2
CD38 & CD45 too low
CD58 overexpression

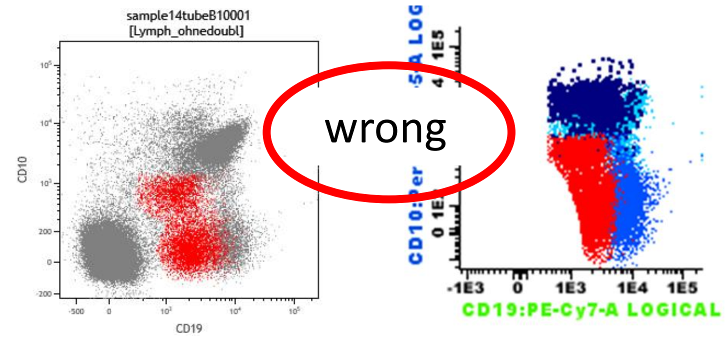

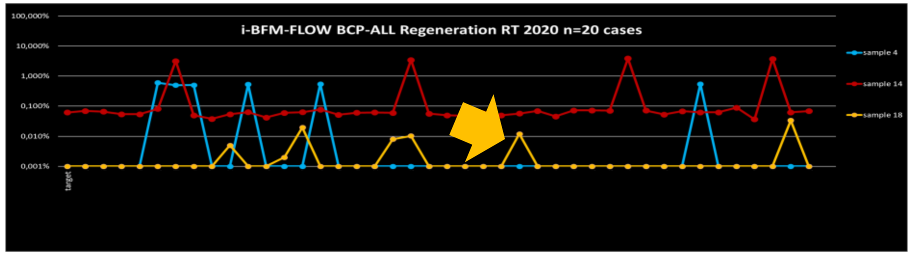

Case 18

RQ-PCR neg

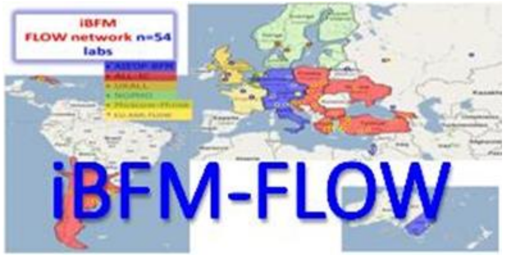

Target vote: negative

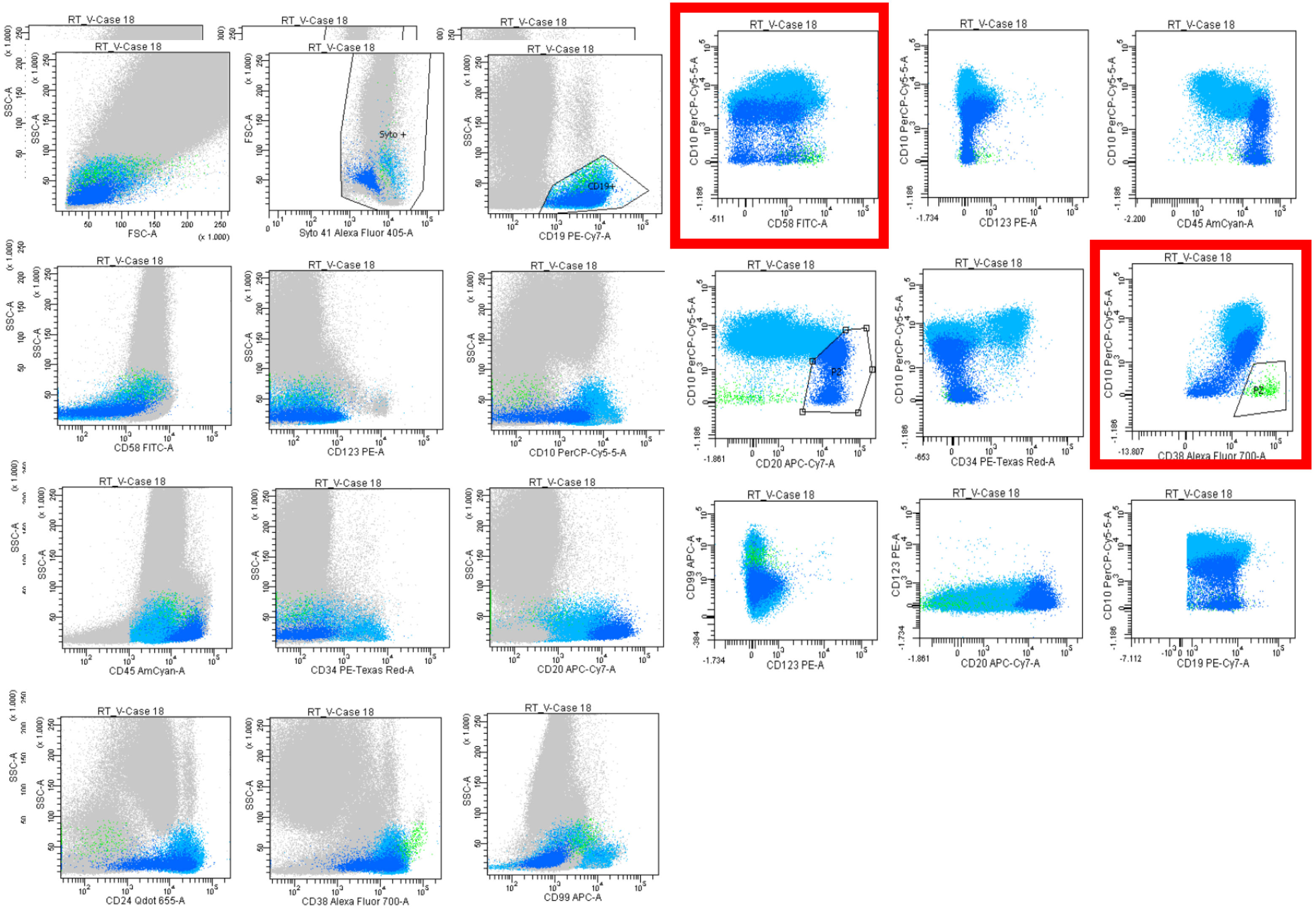

Problem:

Strong early BCP 1 & 2-regeneration

Solution:

mind very compact patterns e.g. in CD38, CD45

No aberration in any marker

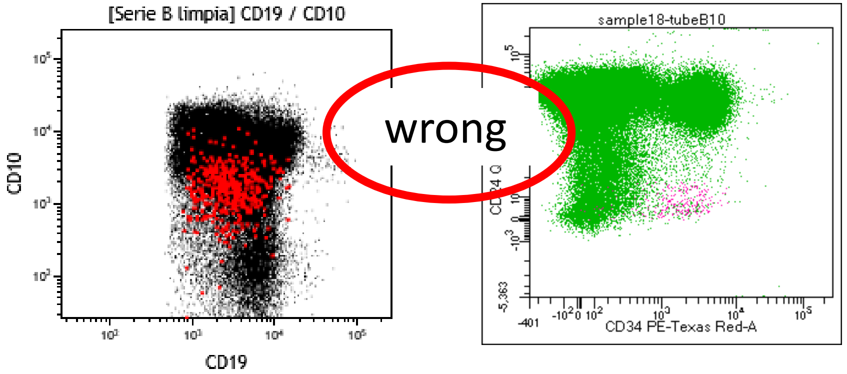

BCP - REGENERATION LMD MRD trial 2017 (RT6)

Analysis of problematic cases

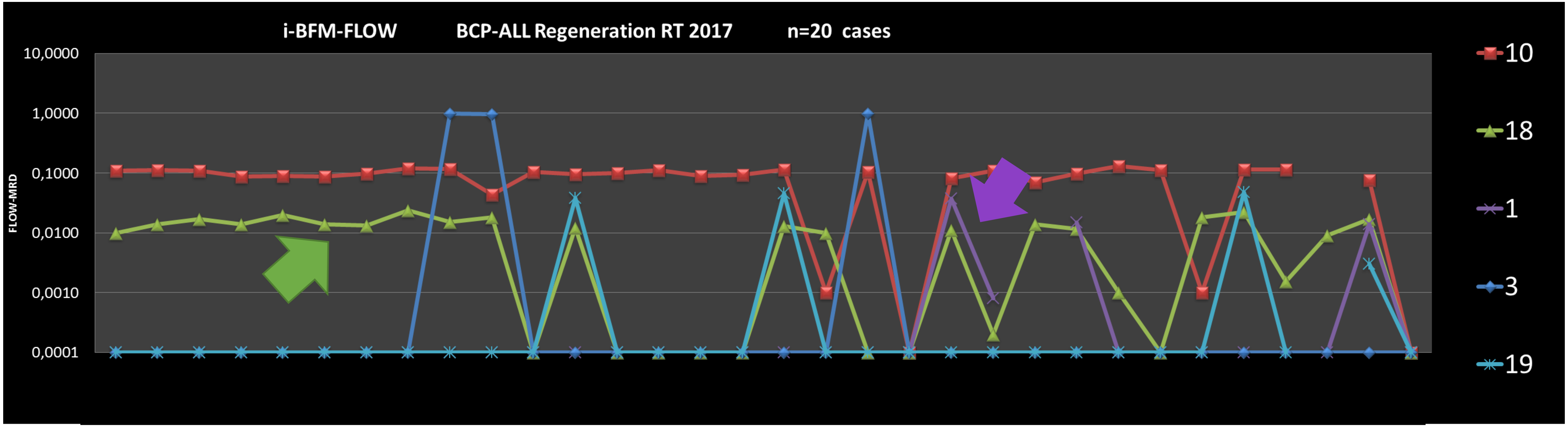

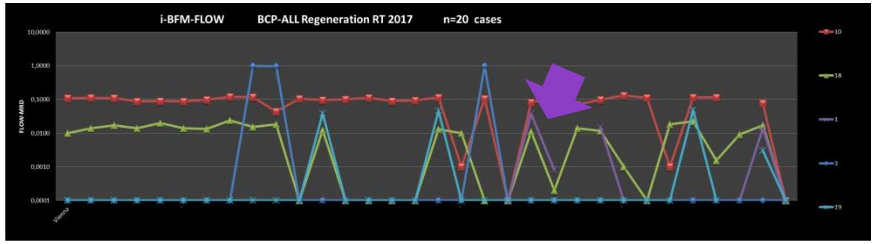

Case 1

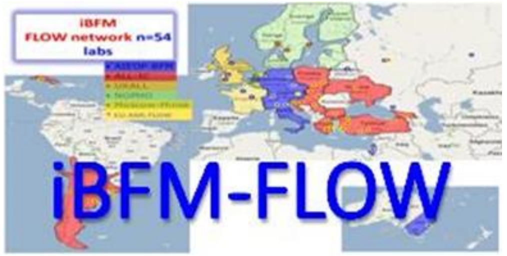

Target vote: negative

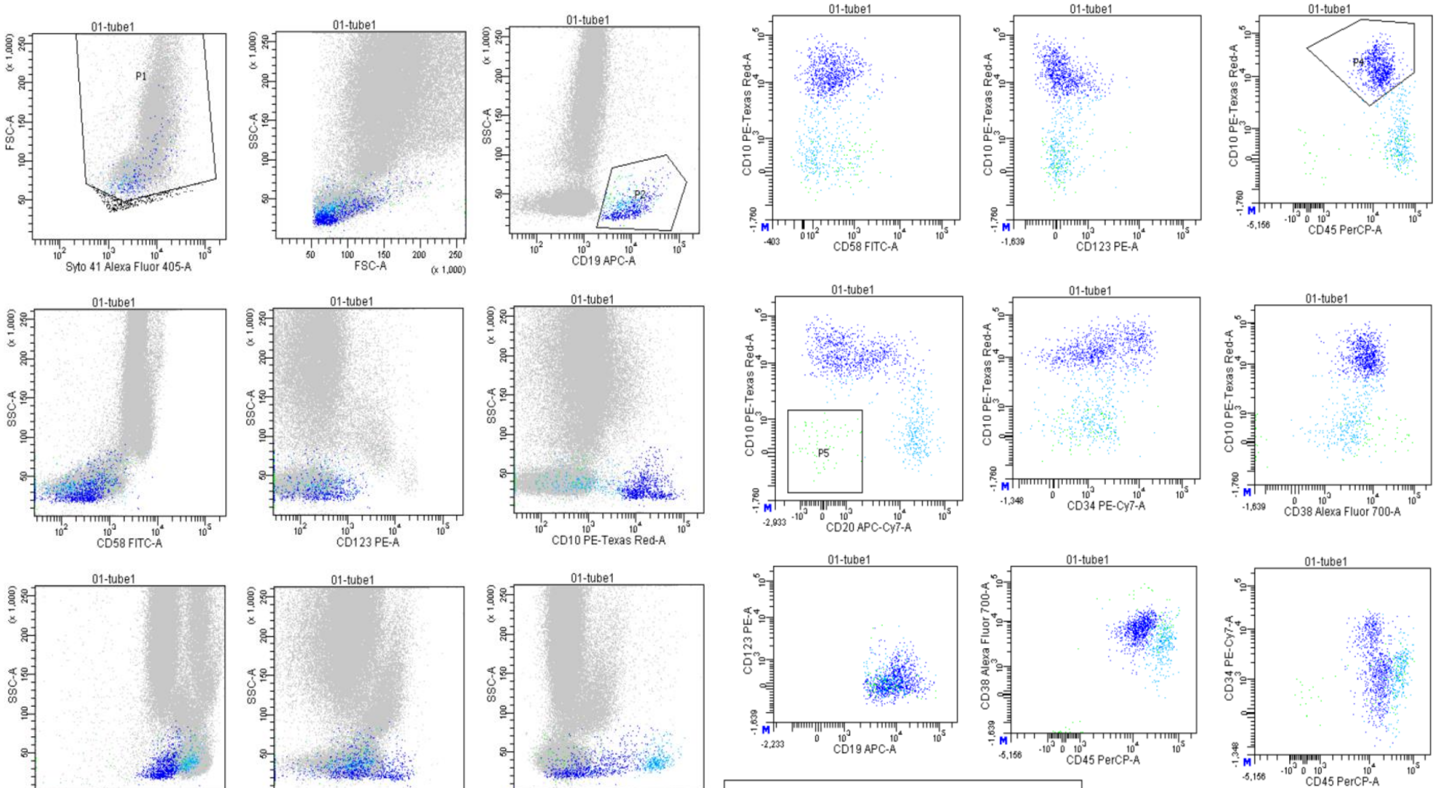

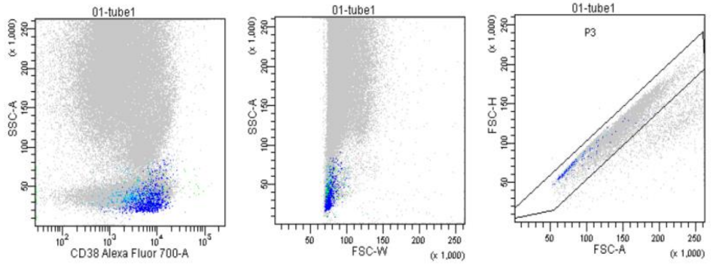

Tube: tube1

Population	#Events	%Parent	%Total
All Events	122,005	####	100.0
P1	120,361	98.7	98.7
P2	1,254	1.0	1.0
P3	1,219	97.2	1.0
P4	818	67.1	0.7
P5	66	5.4	0.1

Specimen Nam... 01 PATIENT:
Record Date: Apr 26, 2016 2:...

Population	#Events	%Parent	%Grand Par...	%Total
P1	120,361	98.6525	####	98.6525
P2	1,254	1.0419	1.0278	1.0278
P3	1,219	97.2089	1.0128	0.9991
P4	818	67.1042	65.2313	0.6705
P5	66	5.4143	5.2632	0.0541

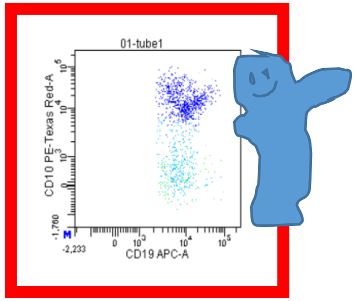

Problem:

early & intermediate BCP-regeneration

Mind „the funny ghost-view“ in the CD19 vs. CD10 plot

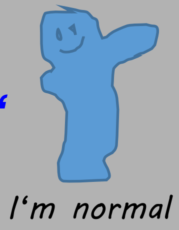

Solution:

mind cluster gating – do not construct populations (by Boolean connections

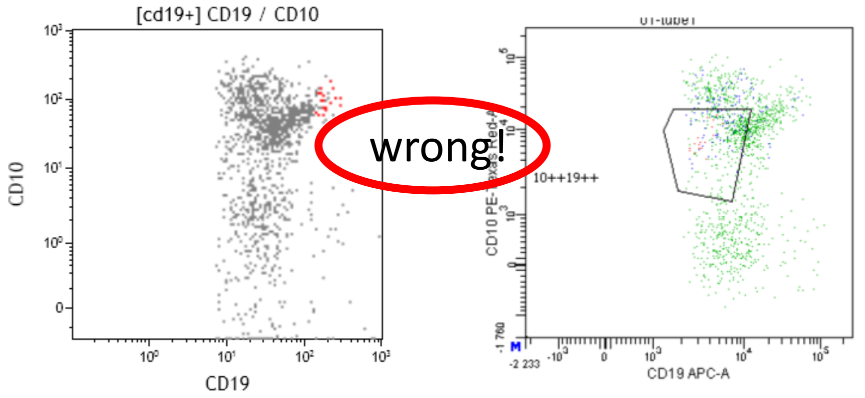

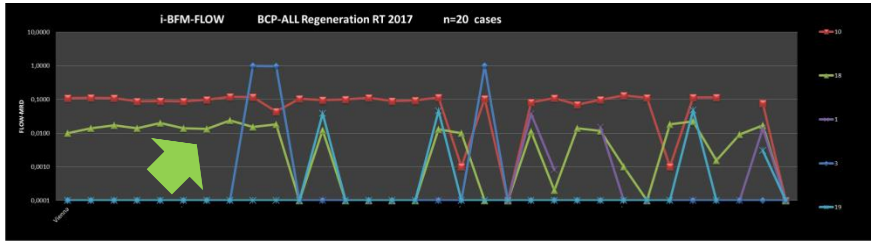

Case 18

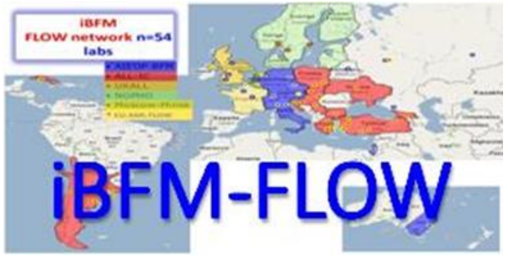

Target vote: positive ~0.01%

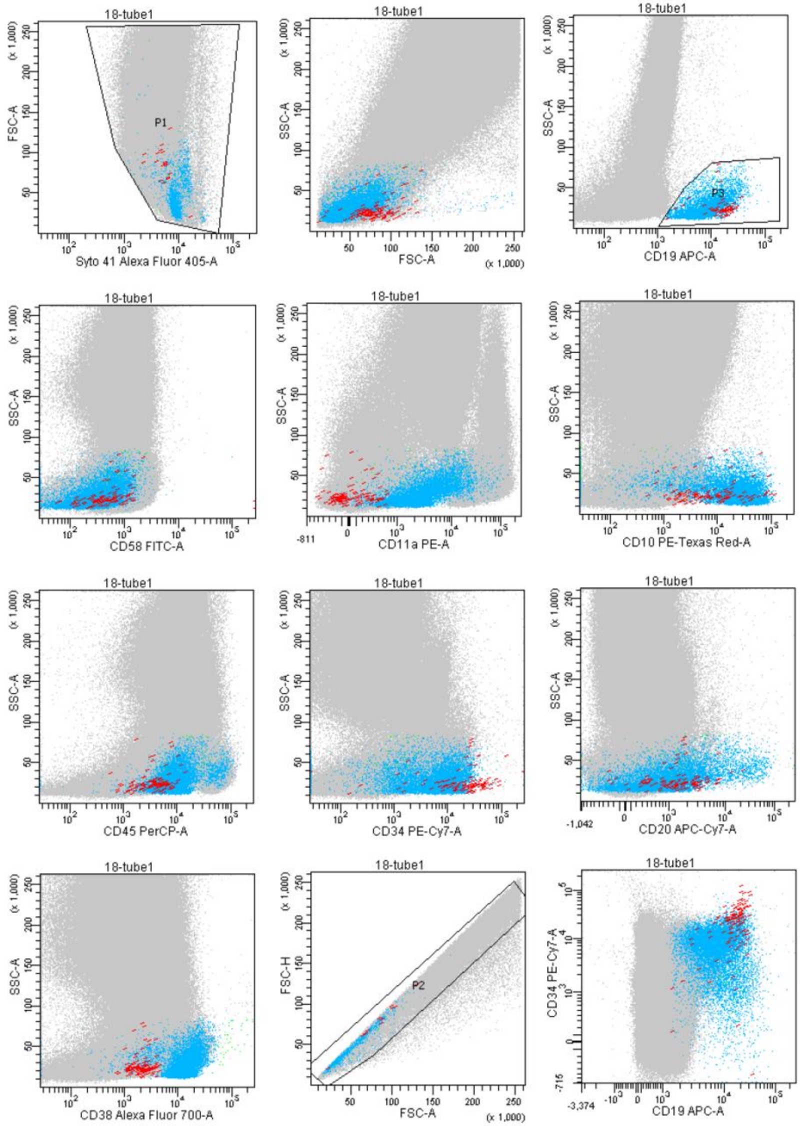

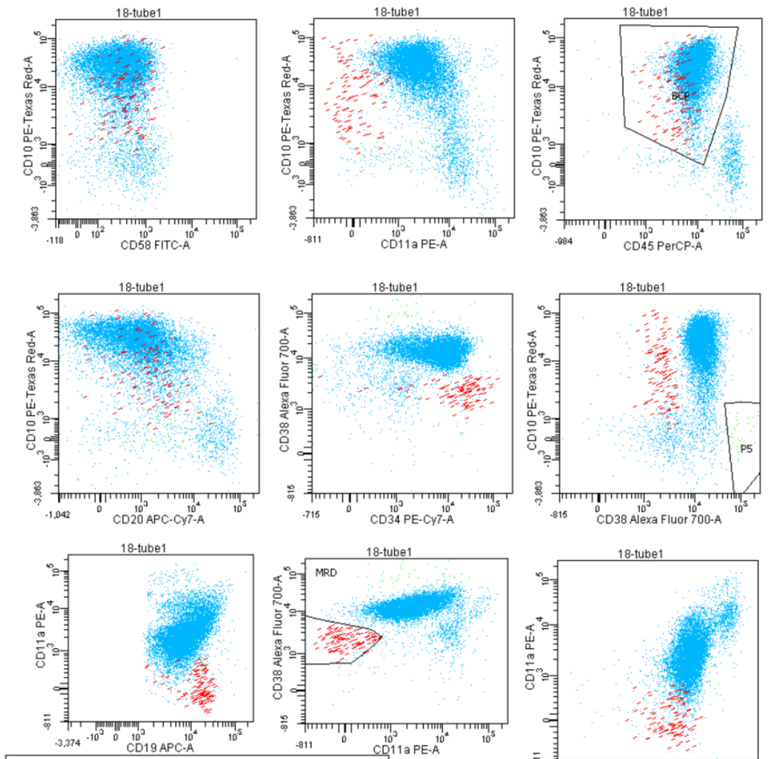

Tube: tube1

Population	#Events	%Parent	%Total
All Events	1,000,000	####	100.0
P1	1,000,000	100.0	100.0
P2	956,734	95.7	95.7
P3	10,814	1.1	1.1
BCP	10,013	92.6	1.0
MRD	107	1.1	0.0
P5	36	0.3	0.0

Specimen Nam... 18 PATIENT:
Record Date: Nov 30, 2016 1...

Population	#Events	%Parent	%Total
P1	1,000,000	100.0000	100.0000
P2	956,734	95.6734	95.6734
P3	10,814	1.1303	1.0814
BCP	10,013	92.5929	1.0013
P5	36	0.3329	0.0036
MRD	107	1.0686	0.0107

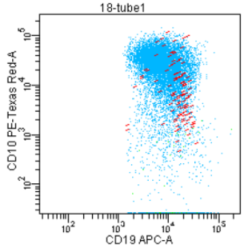

Problem:

strong EARLY BCP-regeneration

insufficient separation by classical markers like CD10

Solution:

mind CD11a (!), CD38

BCP - REGENERATION LMD MRD trial 2019 (RT8)

Analysis of problematic cases

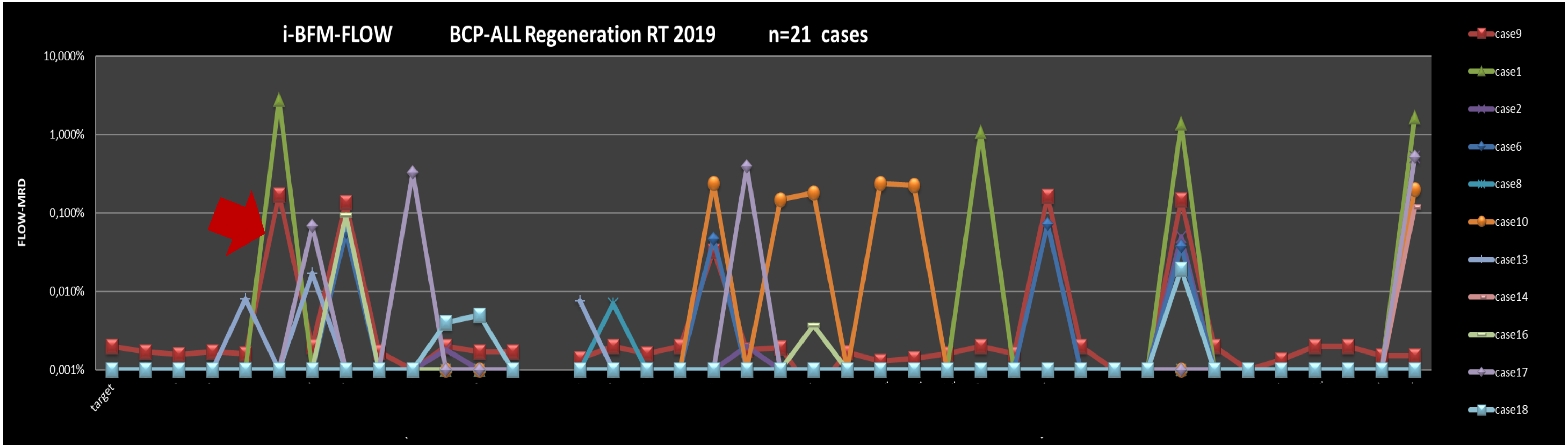

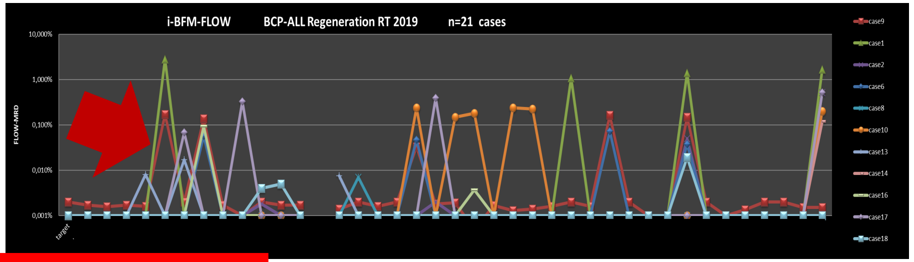

Case 9

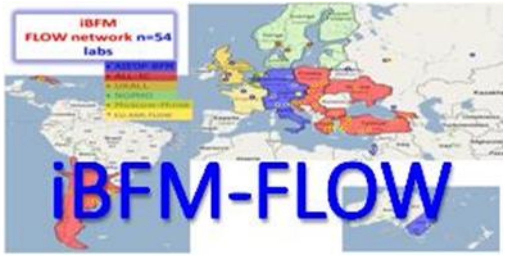

Target vote: positive ~0.002%

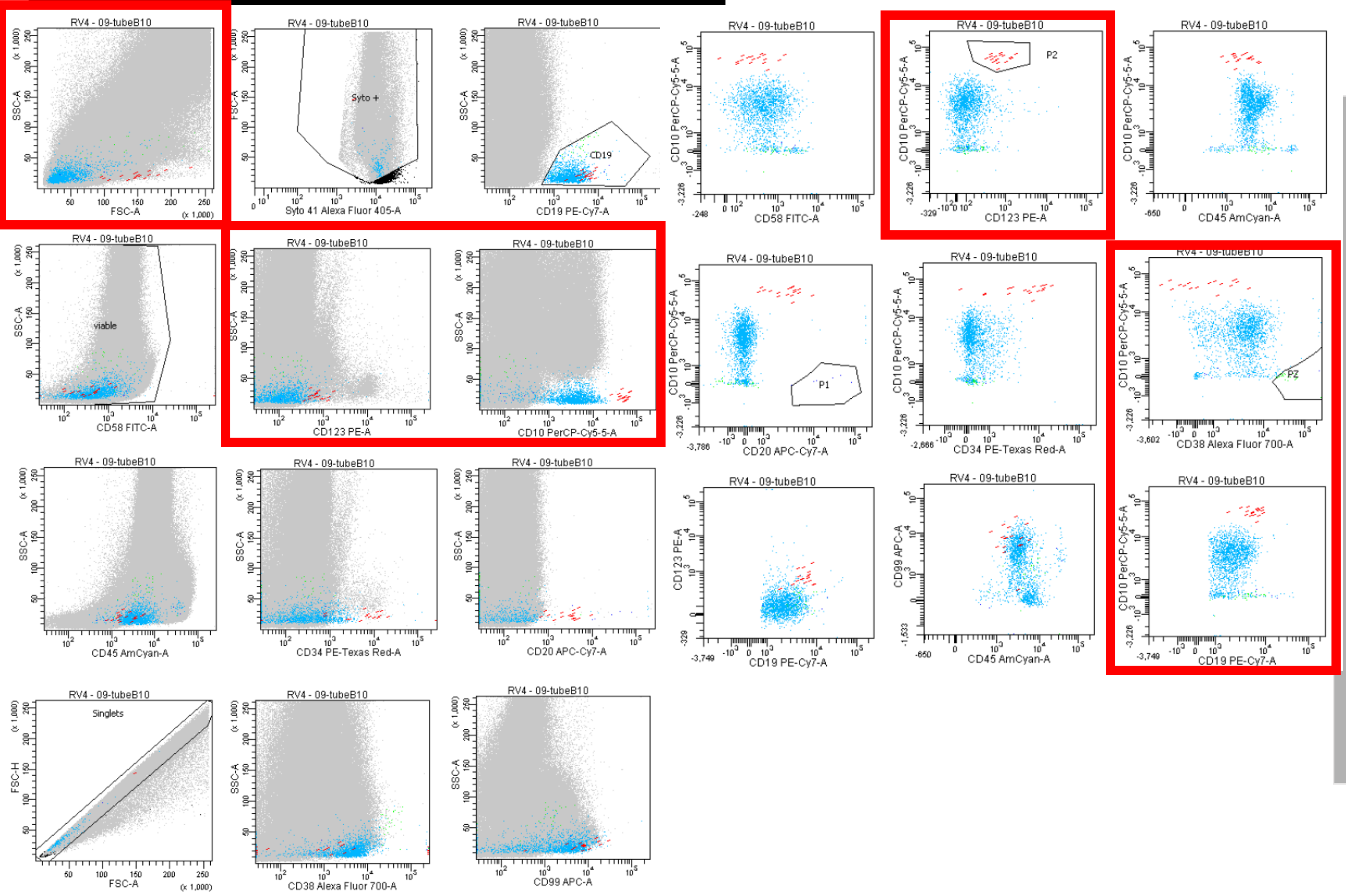

Problem:

Very tiny population

Coexistence of blasts & BCP 1&2

Dim/neg CD34 on BCP1

Solutions: MANY

mind FSC (higher than BCP 1&2)
CD10 overexpression
CD19 higher than BCP 1&2
CD38 (!! too low)
CD123 positive

Reverse population (BCP 1&2): no aberration (apart from CD34)

BCP - REGENERATION LMD MRD trial 2018 (RT7)

Analysis of problematic cases

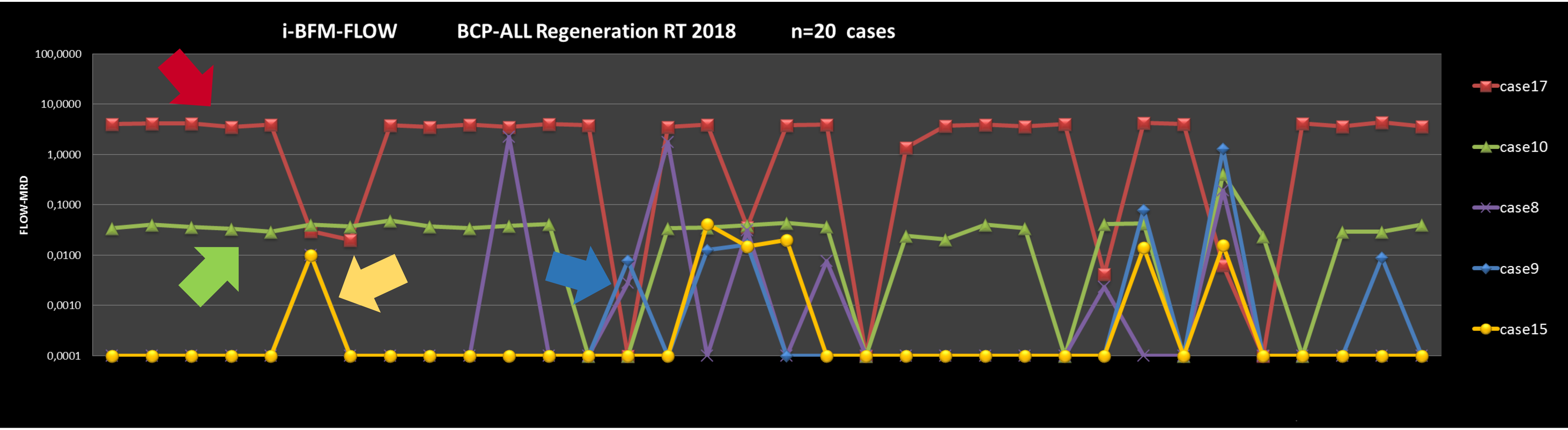

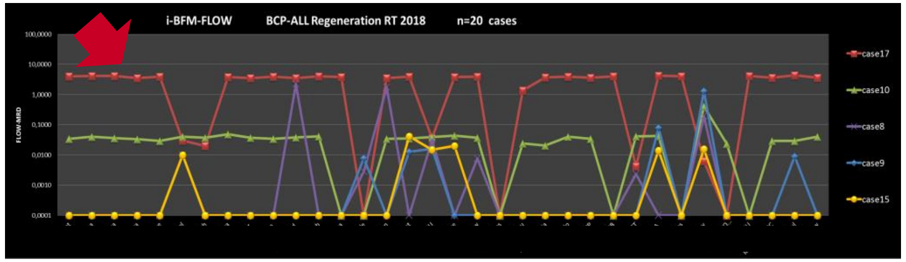

Case 17

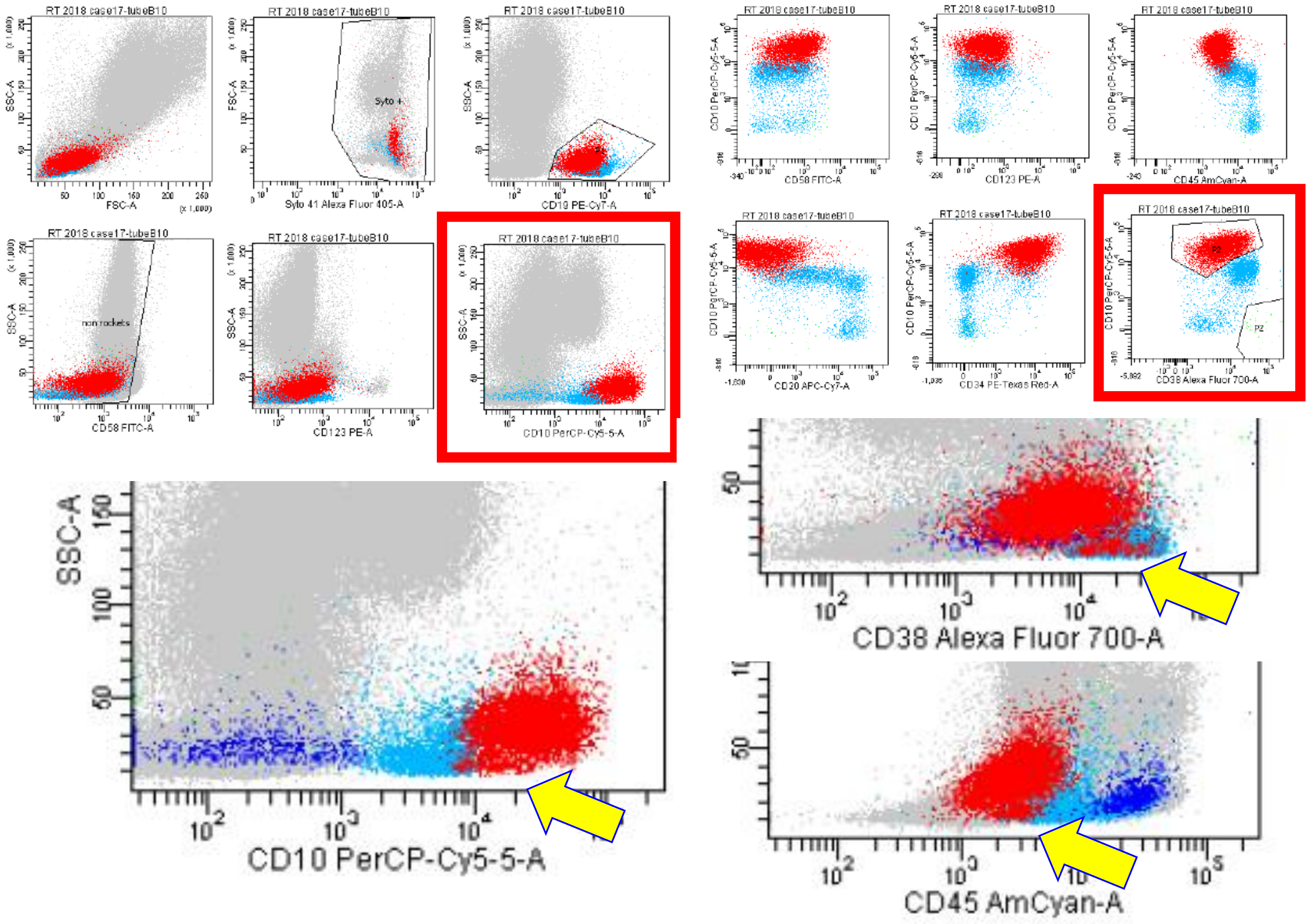

Problem:

Probable EARLY BCP-regeneration

Insufficient separation by classical markers like CD10

Solution:

Mind elevated SSC – should be very low in normal BCP1

Mind unusually heterogeneous CD38

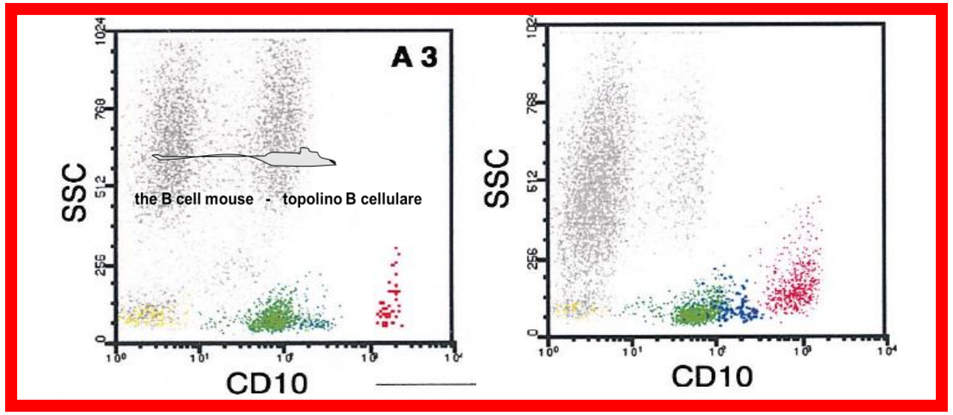

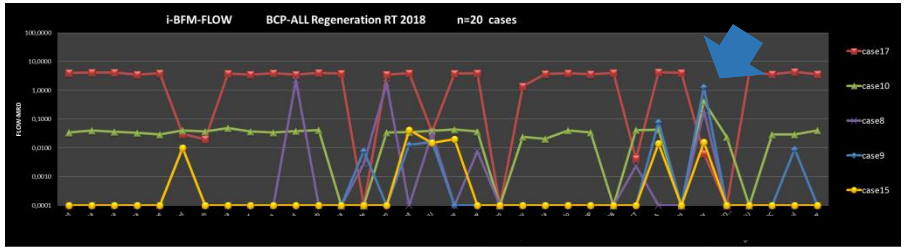

Case 9

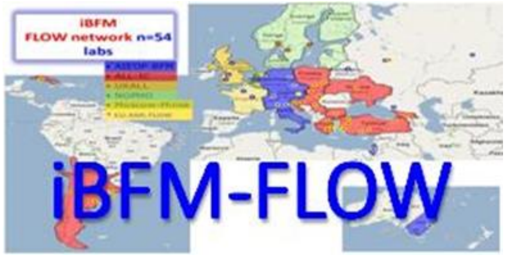

Target vote: negative

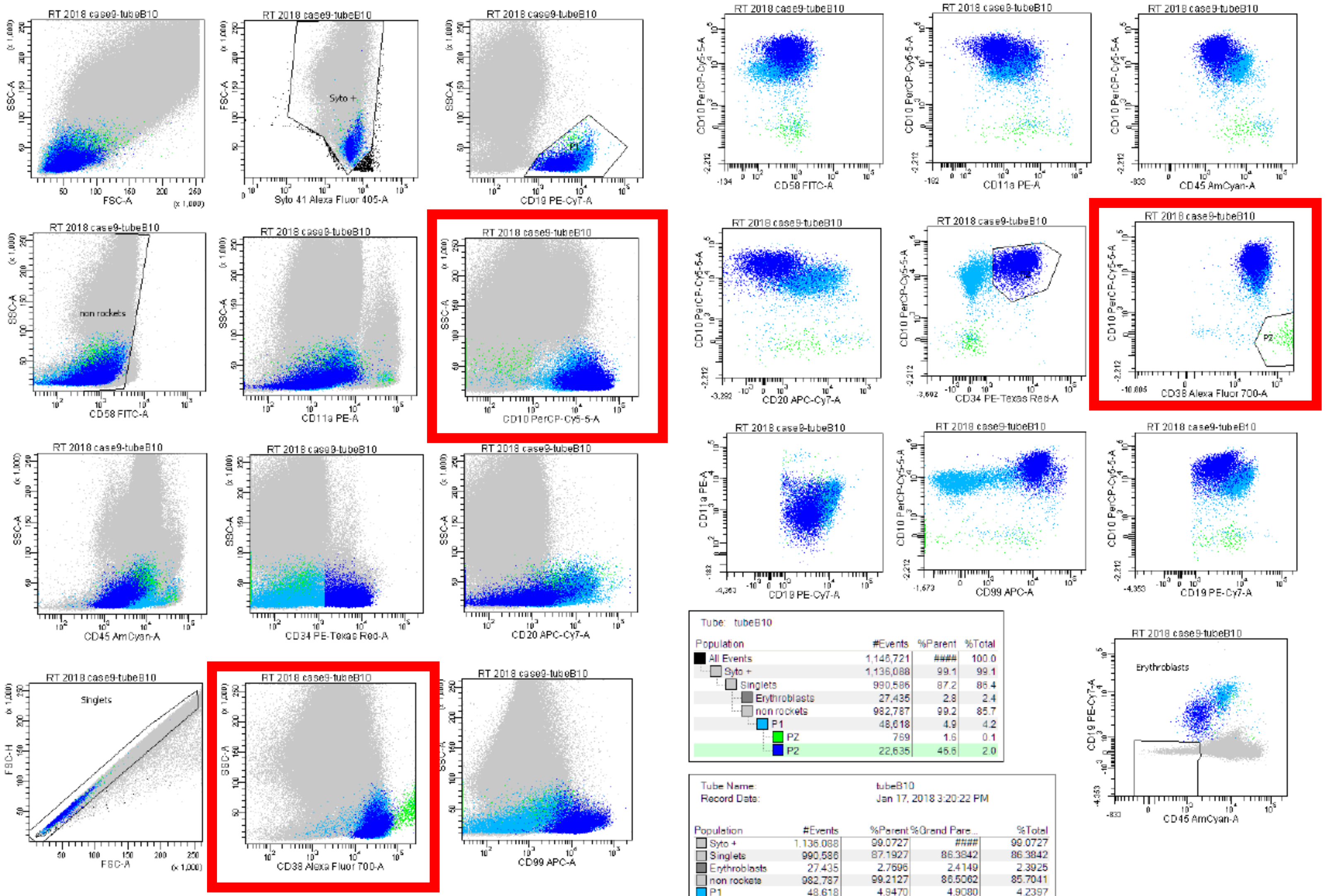

Tube: tubeB10

Population	#Events	%Parent	%Total
All Events	1,146,721	####	100.0
Syto +	1,136,088	99.1	99.1
Singlets	990,586	87.2	86.4
Erythroblasts	27,435	2.8	2.4
non rockets	982,787	99.2	85.7
P1	48,618	4.9	4.2
PZ	769	1.6	0.1
P2	22,635	46.6	2.0

Tube Name: tubeB10
Record Date: Jan 17, 2018 3:20:22 PM

Population	#Events	%Parent	%Grand Pare...	%Total
Syto +	1.136.088	99.0727	####	99.0727
Singlets	990,586	87.1927	86.3842	86.3842
Erythroblasts	27.435	2.7696	2.4149	2.3925
non rockets	982,787	99.2127	86.5062	85.7041
P1	48,618	4.9470	4.9080	4.2397
PZ	769	1.5817	0.0782	0.0671
P2	22,635	46.5568	2.3031	1.9739

Problem:

Strong early BCP 1 & 2-regeneration
Almost no mature BCP3 for comparison

Solution:

mind very compact patterns
e.g. in CD38, CD45

No aberration in any marker

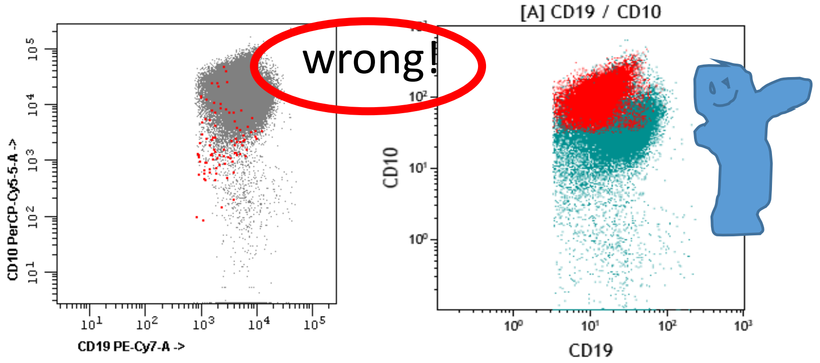

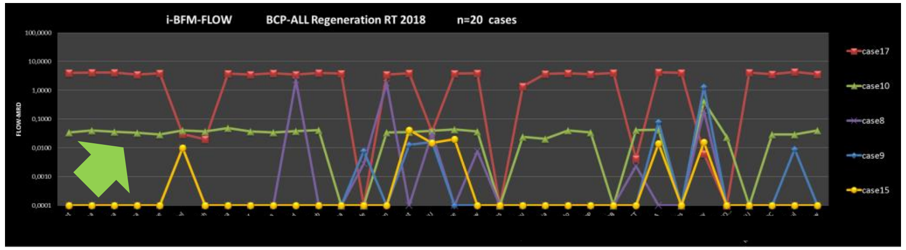

Case 10

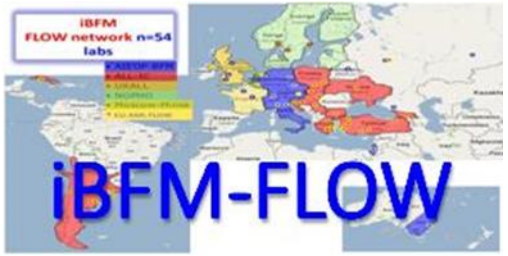

Target vote: positive ~0.035%

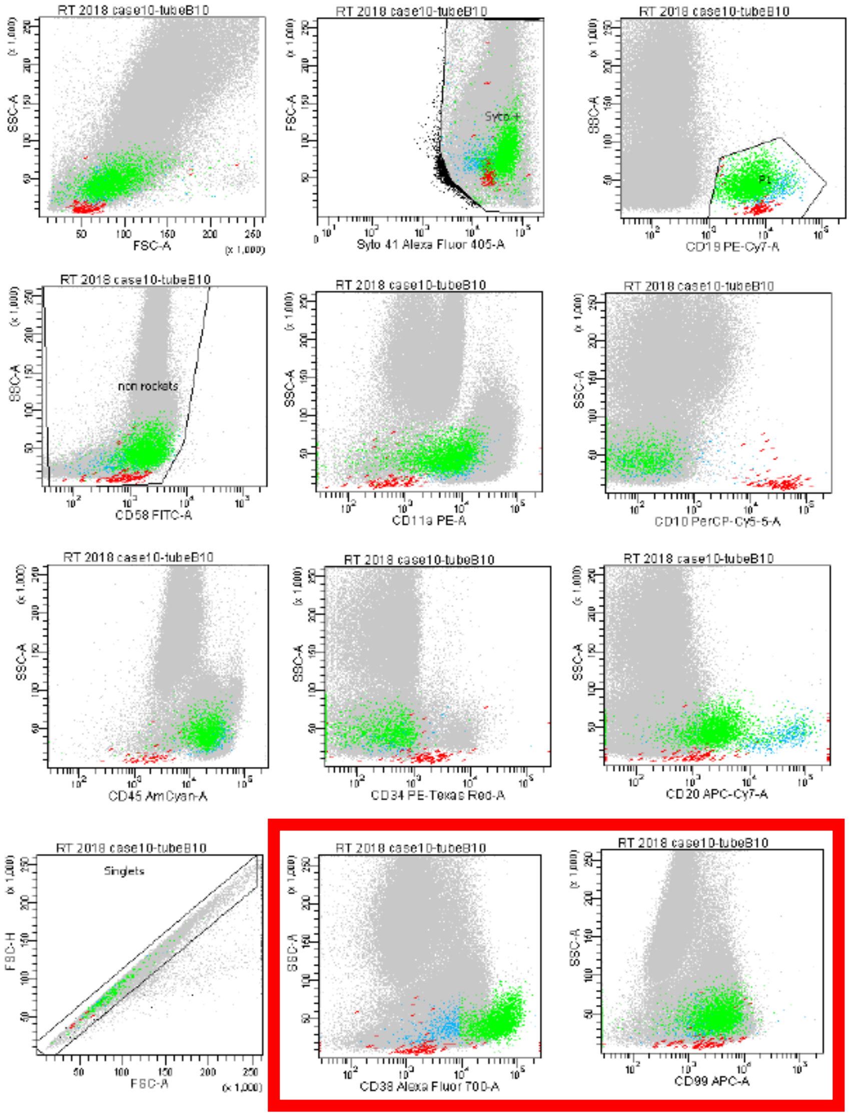

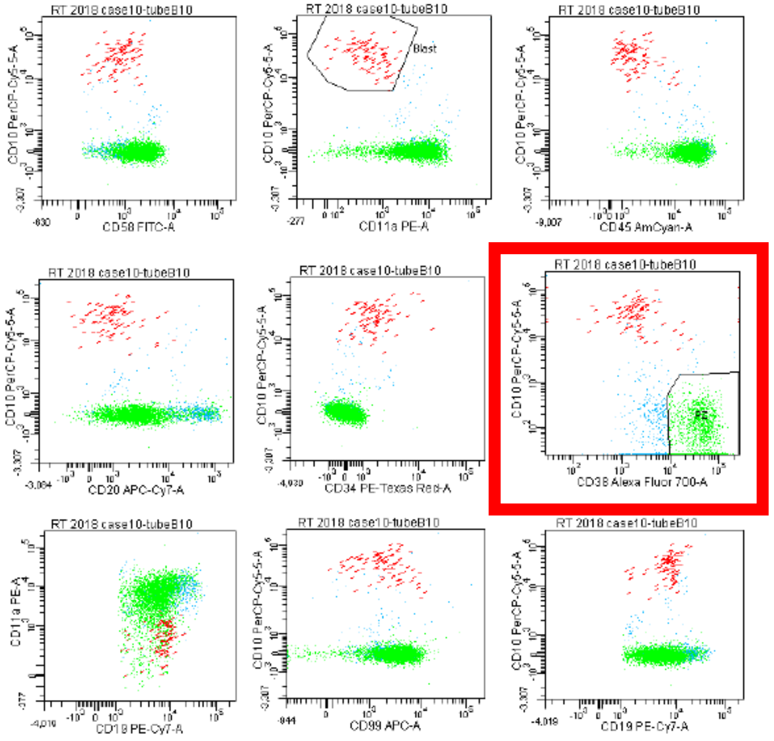

Tube: tubeB10

Population	#Events	%Parent	%Total
All Events	241,567	####	100.0
Syto +	237,516	98.3	98.3
Singlets	222,349	93.6	92.0
Erythroblasts	38,280	17.2	15.8
non rockets	214,405	96.4	88.8
P1	4,384	2.0	1.8
Blast	90	2.1	0.0
PZ	3,693	84.2	1.5

Tube Name: tubeB10
Record Date:

Population	#Events	%Parent	%Grand Pare...	%Total
Syto +	237,516	98.3230	####	98.3230
Singlets	222,349	93.6143	92.0444	92.0444
Erythroblasts	38,280	17.2162	16.1168	15.8465
non rockets	214,405	96.4272	90.2697	88.7559
P1	4,384	2.0447	1.9717	1.8148
Blast	90	2.0529	0.0420	0.0373
PZ	3,693	84.2381	1.7224	1.5288

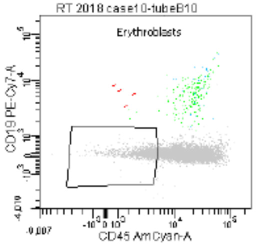

Problem:

Probable early BCP-regeneration, but very few maturing B-cells for comparison (mostly plasma cells)

Insufficient separation by classical markers like CD10

Very small cells with very low SSC – reminiscent of normal BCP1

Solution:

mind CD99 and CD38 (!! too low)

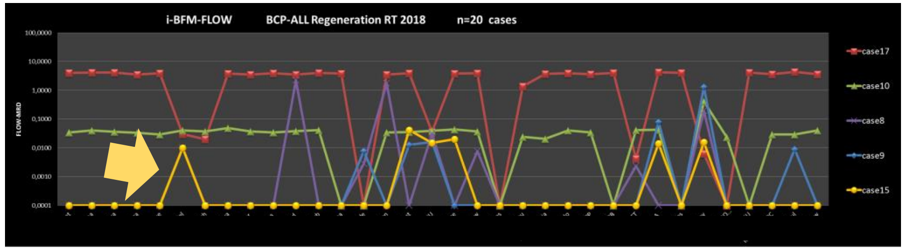

Case 15

Target vote: negative

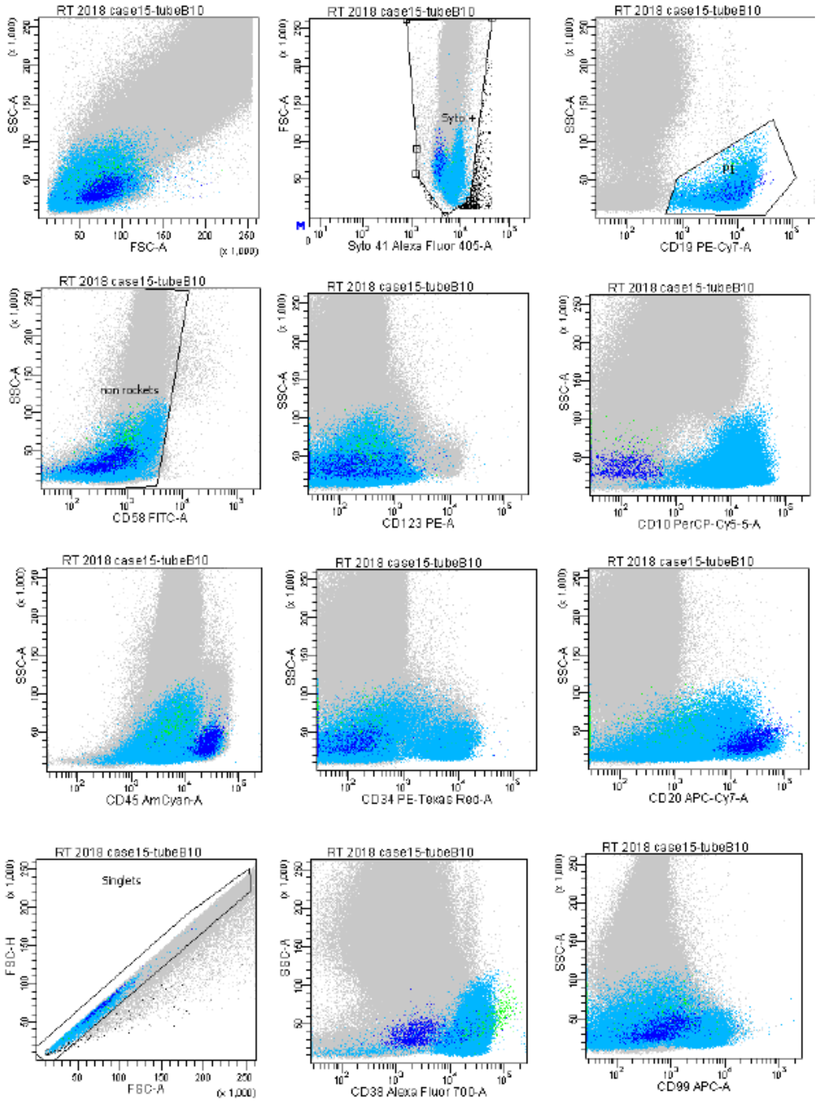

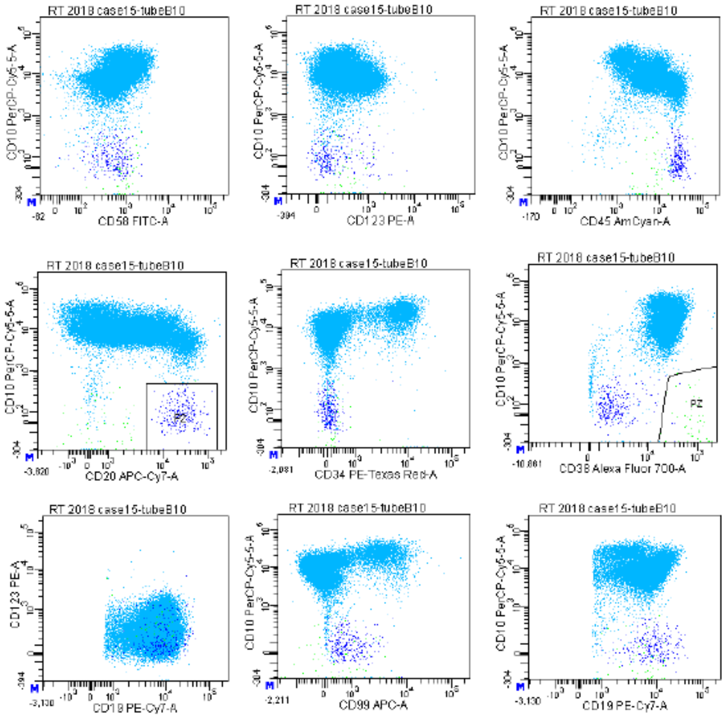

Tube: tubeB10

Population	#Events	%Parent	%Total
All Events	1,146,557	####	100.0
Syto +	1,138,628	99.3	99.3
Singlets	993,554	87.3	86.7
Erythroblasts	101,267	10.2	8.8
non rockets	990,306	99.7	86.4
P1	205,323	20.7	17.9
PZ	303	0.1	0.0
P2	1,279	0.6	0.1

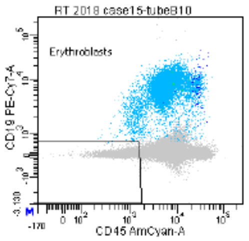

Tube Name: tubeB10
Record Date: Feb 2, 2018 3:12:28 PM

Population	#Events	%Parent	%Grand Pare...	%Total
Syto +	1.138.628	99.3085	####	99.3085
Singlets	993,554	87.2589	86.6554	86.6554
Erythroblasts	101.267	10.1924	8.8938	8.8323
non rockets	990,306	99.6731	86.9736	86.3722
P1	205,323	20.7333	20.6655	17.9078
PZ	303	0.1476	0.0306	0.0264
P2	1,279	0.6229	0.1292	0.1116

Problem:

Strong early BCP 1 & 2-regeneration

Solution:

mind very <u>compact patterns</u>
e.g. in CD38, CD45

No aberration in any marker
