# Supplementary figures and images for: An Extensive Quality Control and Quality Assurance (QC/QA) Program Significantly Improves Inter-Laboratory Concordance Rates of Flow-Cytometric Minimal Residual Disease Assessment in Acute Lymphoblastic Leukemia: An I-BFM-FLOW-Network Report
Source: Cancers (Basel). 2021 Dec 6;13(23):6148. doi: 10.3390/cancers13236148 (PMC8656726; doi:10.3390/cancers13236148)

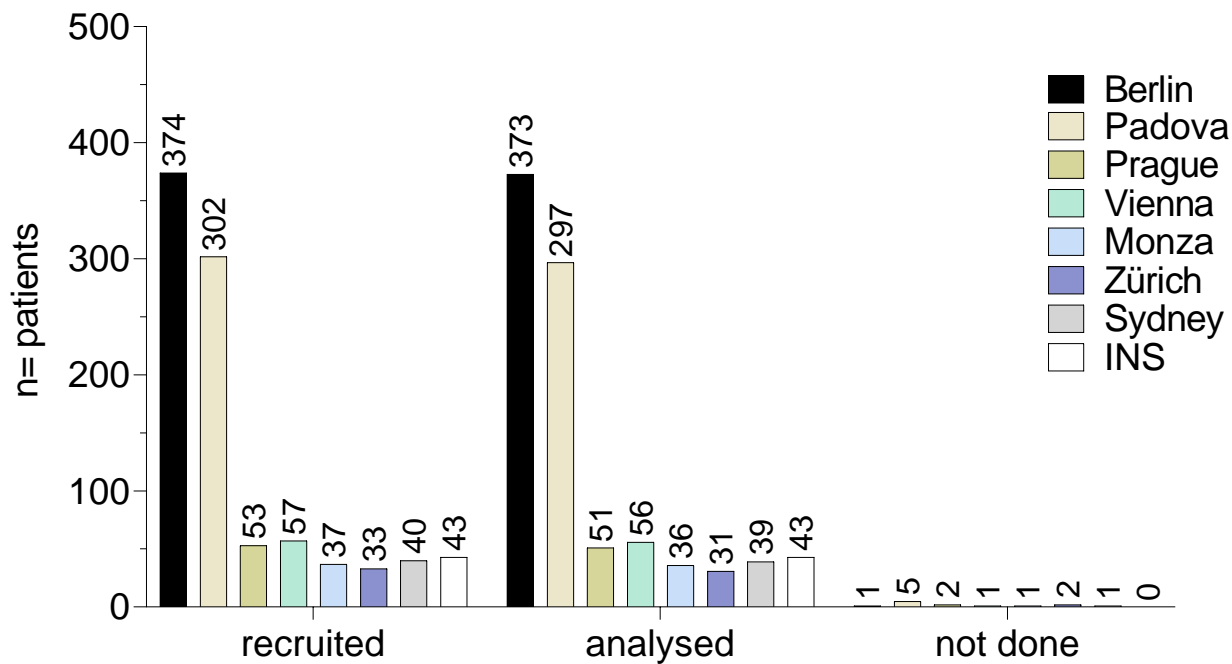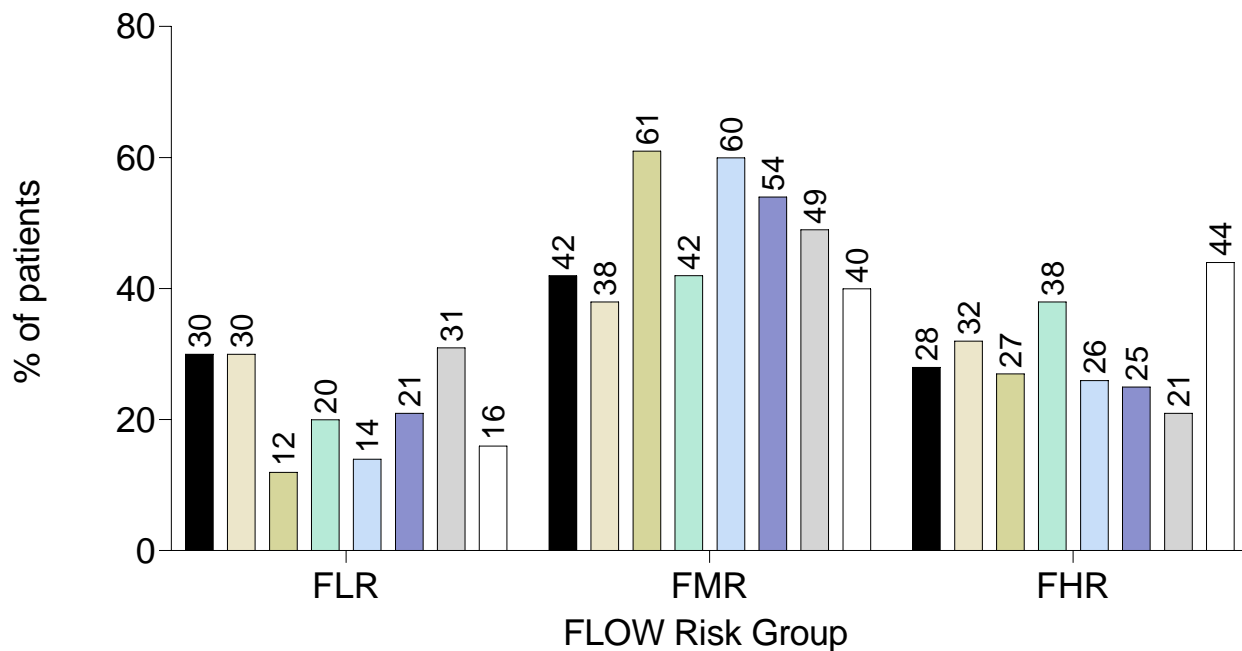

Supplement: Supplementary file 1 [file cancers-13-06148-s001.zip › Figure S2.pdf]
